# Recent Advances in the Application of ATRP in the Synthesis of Drug Delivery Systems

**DOI:** 10.3390/polym15051234

**Published:** 2023-02-28

**Authors:** Matylda Szewczyk-Łagodzińska, Andrzej Plichta, Maciej Dębowski, Sebastian Kowalczyk, Anna Iuliano, Zbigniew Florjańczyk

**Affiliations:** Faculty of Chemistry, Warsaw University of Technology, Noakowskiego 3, 00-664 Warsaw, Poland

**Keywords:** drug delivery system, drug nanocarrier, micelle, ATRP, block copolymer, polymer–drug conjugate, polymersome, polyplexes, self-assembly, branched copolymer

## Abstract

Advances in atom transfer radical polymerization (ATRP) have enabled the precise design and preparation of nanostructured polymeric materials for a variety of biomedical applications. This paper briefly summarizes recent developments in the synthesis of bio-therapeutics for drug delivery based on linear and branched block copolymers and bioconjugates using ATRP, which have been tested in drug delivery systems (DDSs) over the past decade. An important trend is the rapid development of a number of smart DDSs that can release bioactive materials in response to certain external stimuli, either physical (e.g., light, ultrasound, or temperature) or chemical factors (e.g., changes in pH values and/or environmental redox potential). The use of ATRPs in the synthesis of polymeric bioconjugates containing drugs, proteins, and nucleic acids, as well as systems applied in combination therapies, has also received considerable attention.

## 1. Introduction

Polymeric systems for controlled drug release have been the subject of intensive academic and industrial research for more than half a century, with the aim of extending the period of therapeutic action, enabling drug delivery to a specific site in the body, and reducing the negative side effects induced by bioactive substances. The mechanism of action of these systems is very diverse, ranging from the protection of active pharmaceutical ingredients from an aqueous living environment for a programmed time period to the targeting and formation of conjugates with polymers covalently attached to drugs to increase their stability and immunogenicity [1,2,3,4,5,6,7]. Most of the recent studies have focused on biocompatible nanocarriers, such as micelles, vesicles (polymersomes), nanogels, dendrimers, and hybrid nanoparticles with porous inorganic cores [8,9,10,11,12,13,14,15,16,17,18,19,20,21]. The reduction in polymeric containers to submicron sizes (typically 10 to 200 nm) allows for the delivery of drugs in the form of a stable colloidal dispersion, hence promoting more efficient absorption of therapeutic loads when compared with larger carriers. In addition, the large surface area of nanoparticles provides space for functionalization, targeting, and bioconjugation. Therefore, nanocarriers can also exhibit other desirable properties, such as biodegradability, the ability to change size and permeability under the influence of external stimuli (e.g., temperature, pH), or the formation of multicompartment containers, in which the simultaneous loading and release of different drugs are possible [10]. The simplest and most common method of assembling polymers into well-defined nanoparticles is the self-organization of amphiphilic block copolymers in water or water–oil systems. By adjusting the macromolecular properties of the copolymers and the self-organization conditions, various nanostructures can be formed, including separated micelles or polymersomes, and cluster aggregates. Amphiphilic block copolymers are also often used in resorbable implantable plates, containing a drug with targeted cytotoxicity that can be inserted directly into the area altered by pathological cells [7].

Modern polymer chemistry offers many synthetic strategies that can be used to prepare block copolymers with specific macromolecular architecture, composition, functionality, and low-molecular-weight dispersity. These include various types of living and controlled polymerization, often combined with post-polymerization selective chemical modification of the terminal functional groups [21]. In the past two decades, controlled radical polymerization (CRP) methods, particularly reversible addition–fragmentation chain transfer (RAFT) polymerization and atom transfer radical polymerization (ATRP) [22,23,24,25,26,27,28,29,30,31,32,33], have become some of the leading tools in the synthesis of block copolymers. They can be carried out in a variety of solvents, including water, and are tolerant of most functional groups. The CRP method is typically used to obtain segments derived from active vinyl monomers (e.g., (meth)acrylates, (meth)acrylamides, styrene derivatives), which are combined with other synthetic or natural polymers employing end groups that can act as CRP initiators or reactants in “click” reactions. This strategy has been successfully applied to build multi-block linear chains and to construct macromolecules with a more complex topology, such as star-like polymers, comb-like polymers, hyperbranched structures, networks, and hybrids, with a covalent bond attachment of well-defined functional polymers to therapeutics or other polymer-grafted materials. Many attempts have been made to use these materials in drug delivery systems and early-stage results have been described in comprehensive articles published in 2009 and 2012 [34,35]. Nowadays, the field is growing rapidly and many exciting new results have been published in the last decade.

In this paper, we would like to present some new developments in the ATRP-utilized design of novel polymeric structures for drug delivery systems (DDSs). For the purpose of this review, DDSs obtained via ATRP are divided into the four most recently studied types: micelles, polymersomes, and polyplexes formed by linear block copolymers; carriers formed by branched copolymers; hybrid nanoparticles; and bioconjugates.

*We dedicate this article to the late Professor Andrzej Dworak, who made tremendous contributions to the development of research on the self-organization processes of amphiphilic polymers and stimuli-responsive materials and their practical use in modern medicine and pharmacy. He was the author of many original papers as well as fundamental review papers, which provide valuable information and inspiration for future generations of chemists undertaking research in this fascinating area of science*.

## 2. The Principles of ATRP

ATRP is a robust polymerization method that was developed and presented by Professor Matyjaszewski’s group in 1995 [36,37]. It was inspired by atom transfer radical addition, which was successfully used in the synthesis of low-molecular-weight compounds [38]. ATRP, next to nitroxide-mediated polymerization or RAFT [39,40], is a method of CRP [41].

**Figure 1:**
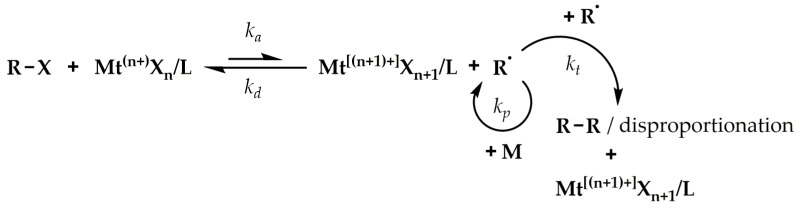
General scheme of an ATRP mechanism.

The idea of ATRP is based on reducing the concentration of radicals in the polymerization system through reversible reactions of the activation and deactivation of the active center as a result of halogen atom transfer, based on a dynamic equilibrium between working radical centers and dormant organic halides, with a relatively low homolytic dissociation activation energy of C-X bonds (Figure 1) [42,43]. Therefore, this equilibrium must be strongly shifted to the left (towards the organic halides R-X). The transfer of the halogen atom (X) from the initiator molecule or the growing polymer chain (R-X) takes place to the catalyst molecule, which is an inorganic salt, i.e., a transition metal halide with two oxidation states differing by one electron (Mt^(n+)^X_n_), complexed with a ligand (L). For accepting a halogen, the catalyst is in a reduced form and acts as an activator (Mt^n+^X_n_), increasing its degree of oxidation, and the process itself is an activation reaction (*k_a_*). As a result of activation, a radical (R^•^) is formed capable of attaching to monomer (M) molecules (initiation or propagation (*k_p_*) stage) or other reactions typical of radical polymerization. However, the participation of termination (reaction with the other radical, *k_t_*) is drastically diminished by reducing the concentration of radicals to a level several orders of magnitude lower than that of typical radical polymerization. During the operation of the radical, an appropriate number of monomer molecules are attached to the growing chain. In contrast, the number of attached monomers per one act of activation depends on many rate constants, including those that affect the ATRP equilibrium (*k_a_*, *k_d_*). The latter, in turn, depends on the structure of the organic halide and the type of catalyst, and in particular on the type of ligand whose task is to create a complex with an inorganic salt that is soluble in organic media and to give the catalyst an appropriate reduction potential [44]. It is worth noting that most often the role of ATRP ligands is played by polydentate nitrogen compounds, including aliphatic, cycloaliphatic, or aromatic amines [45,46]. However, other systems, for example based on phosphorus compounds, are also used [47,48]. In the next stage, the oxidized catalyst (deactivator, Mt^[(n+1)+]^X_n+1_/L), having an additional halogen atom, reacts with one of the radicals present in the system, transferring a halide to it, which results in the formation of a dormant form of the polymerization center and the reduction of the catalyst. Due to the statistical nature of the activation and deactivation reactions, random organic halides and radicals undergo it, respectively, which, with a sufficiently fast deactivation, ensures a relatively uniform growth in all the polymer chains present in the system (linear increase in average molar mass with the degree of monomer conversion and a small dispersity in the molar masses of the obtained polymer). Copper salts were one of the first and the best-known catalytic systems used in ATRP; numerous studies have confirmed the possibility of using the halides of other metals, i.e., Fe [47,48,49,50], Ru [51,52], Ga, or Ir [53,54]. The required amount of catalyst in a normal ATRP mechanism is relatively large, as, typically, one molecule of catalyst is used per one initiation site. This has been a huge disadvantage of the method and constitutes a limitation for implementing it into industrial practice.

In the last 15 years, numerous related methods have been developed that use much smaller amounts of catalysts. Such systems, however, require more active ligands and additional agents whose task is to regenerate the activator molecules that were irreversibly formed in the system due to some termination processes and/or the contamination of the system with oxygen. Such agents might be glucose, ascorbic acid, hydrazine, tin(II) compounds (in activators regenerated by electron transfer variant, ARGET) [55], typical radical initiators as sources of radicals (in initiators for continuous activator regeneration variant, ICAR) [29,51,56,57], zero covalent metals, e.g., Cu^0^ (in supplemental activation reducing agent variant, SARA) [58], or external stimuli such as electrical current/potential (in eATRP) [59], and ultrasound in the presence or absence of piezoelectric materials (in mechano/sonoATRP) [60,61]. The other and “greener” type of ATRP is photoinduced organocatalyzed polymerization (O-ATRP), which does not involve any metal catalyst [22,62]. These developments changed ATRP into a technique well suited to the principles of green chemistry [63].

**Figure 2:**
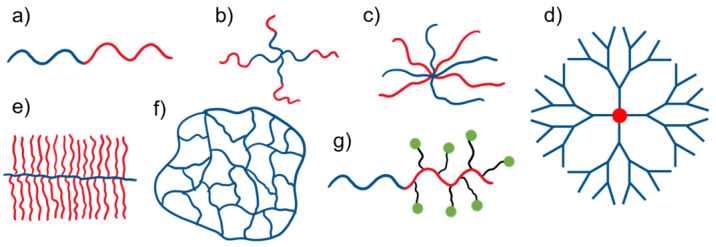
Representative molecular architectures synthesized via ATRP and applied in the formation of polymeric DDSs: (**a**) linear block copolymers, (**b**) regular star copolymers, (**c**) miktoarm star (co)polymers, (**d**) polymer brushes, (**e**) dendrimers, (**f**) nanogels, and (**g**) polymer–drug conjugate.

Activated organic halides are used as initiators of ATRP. They can be simple compounds, such as the commonly used 2-bromobutyric acid ethyl ester, or functional initiators, i.e., those that allow the introduction of an end-group capable of chemical reactions or interactions, e.g., dye moiety [64,65], drug or proteins molecules [66,67,68], stimuli-sensitive molecules [69] or groups able to chemically react with other molecules or chains [70], etc. In some cases, the initiator can be covalently bonded to some surface or is able to influence the topology of macromolecules [71,72]. Therefore, ATRP allows the synthesis of linear, cyclic, and branched polymers, including star-shaped, comb, bottle-brush, and hyperbranched structures, nanogels, and polymer–drug conjugates (Figure 2).

In the case of polymer stars, there are several synthetic strategies possible; they can be obtained via “core-first”, “arm-first”, and “coupling-onto” methods utilizing ATRP. While the “core-first” method uses a multifunctional initiator with a specified number of initiating species, which defines the number of arms, the “arm-first” method relies on ready, halogen functionalized polymer chains that are activated in the presence of the crosslinking agent, so core formation takes place in situ. It does not allow us to control the number of arms in a very good manner, however; it enables the synthesis of stars with structurally different arms (miktoarm). Polymer grafts (combs and bottle-brushes) might be synthesized by the polymerization of macromonomers (obtained by ATRP or in the other way), by the initiation of ATRP with a formed polymer chain comprising initiating species along it, or by coupling the side chains to the main chain consisting of appropriate chemical groups in monomeric units, when the side and main chains may be obtained via ATRP as well [73]. There have been successful attempts at star-brush molecule (“hairy” stars) synthesis as well [74].

It is essential that the halogen atom is formally present at the other end of the polymer chain after polymerization. This provides a vast opportunity to exchange the atom into the other, more reactive group, enabling further reactions, i.e., linking specific molecules to that chain-end or coupling the macromolecule with another one. The example of a halogen-to-azide group exchange best demonstrates this, which then opens the way to a variety of coupling possibilities with alkyne moieties, called “click” chemistry [75].

On the other hand, the residual halogen atom at the end of the macromolecule can be used as a macroinitiator for the synthesis of block copolymers, thus depriving ionic polymerizations of the monopoly for obtaining well-defined segment systems [76,77]. This, in turn, enables the design of materials with specific morphology obtained due to the self-assembly of block copolymers [78]. These include hydrophobic copolymers capable of forming ordered nanostructures from a polymer melt, e.g., lamellae, gyroids, hexagonally packed cylinders, or spheres. It is worth noting that the type of nanostructure can be influenced by the basic structural parameters included in the phase diagrams, i.e., the interaction parameter, degree of polymerization, and volume fractions of components as a predominance factor. However, ATRP allows us to control other subtle parameters, e.g., the topology or dispersion of blocks [79,80], which can change phase boundaries and stabilize phases considered to be thermodynamically metastable, e.g., hexagonal perforated lamellae [81].

**Figure 3:**
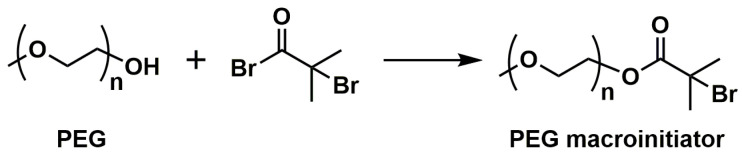
A typical synthesis of PEG macroinitiator of ATRP for amphiphilic block copolymers.

ATRP also enables the synthesis of hydrophobic segments for amphiphilic block copolymers or complete amphiphilic block copolymers and double hydrophilic block copolymers [82,83,84,85]. Amphiphilic materials can self-assemble in aqueous systems to form vesicles (liposomes) or micelles (spherical, cylindrical, hexagonally packed, or lamellar ‘‘neat’’ micelles in parallel arrangement) [86], which are often used in DDSs, for instance. In some cases of doubly hydrophilic copolymers, one of the segments is a polyelectrolyte (often a polyanion), which can interact with particles of inorganic salts, being the basis for artificial biomineralization processes. Among the monomers giving hydrophilic segments in ATRP, acrylamide, 2-hydroxyethyl methacrylate (HEMA), or poly(ethylene glycol) methacrylates should be mentioned [87]. However, polyelectrolytes usually have to be created using hydrophobic precursors, e.g., tert-butyl acrylate (tBuA), which, after polymerization, are hydrolyzed to poly(acrylic acid) (PAA) [88]. The direct use of acrylic acid in polymerization causes its reaction with a deactivator, i.e., copper(II) salts. This shows that although ATRP is quite robust to reaction conditions, this method has some limitations. The main challenge of this method is the limited range of monomers that can be used compared to that in simple radical polymerization. It is required that these monomers have high resonance stabilization. Hence styrenes, acrylates, methacrylates, or acrylonitrile are most often used. At the same time, the well-controlled ATRP of ethylene, butadiene, or vinyl acetate is practically impossible. It should be noted, however, that ATRP allows us to obtain the large family of amphiphilic copolymers by using hydrophilic macroinitiators, such as poly(ethylene glycol) (PEG), and naturally occurring polysaccharides. The typical synthetic approach involves the transformation of the hydroxyl end-group of the hydrophilic reagent with 2-bromoisobutyryl bromide (BIBB) to form an ATRP macroinitiator (Figure 3) [82].

## 3. DDSs Based on Linear Block Copolymers

**Figure 4:**
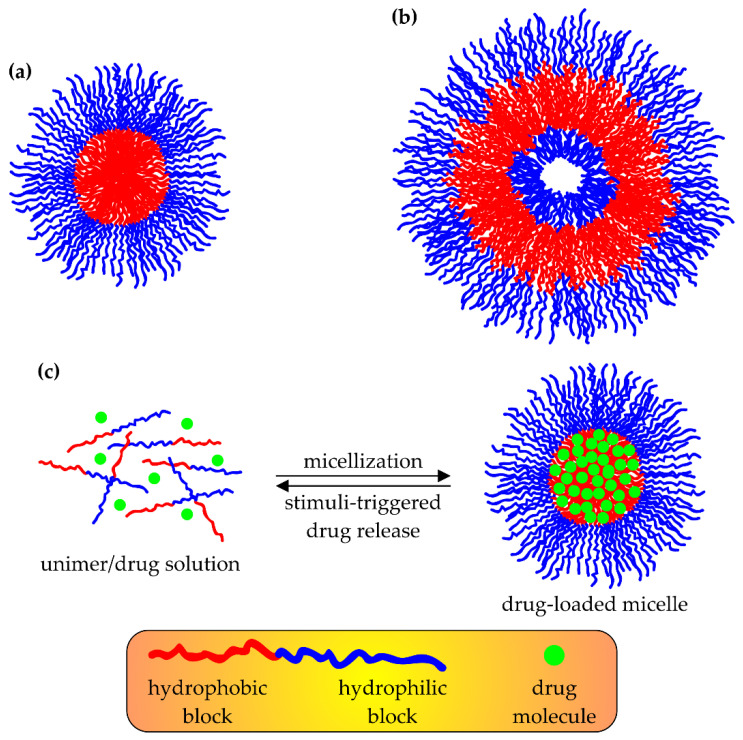
Schematic representations of (**a**) micelle and (**b**) polymersome formed by LBCPs, as well as (**c**) general synthetic strategy applied for the formulation of micelle-based DDSs.

Due to their ability to self-assemble into nanostructures in aqueous media, linear block copolymers bearing both hydrophilic and hydrophobic blocks have gained much attention as potential drug nanocarriers. Although there are several factors that impede the behavior of linear block copolymers (LBCPs) in aqueous dispersions (e.g., the molecular weight of polymeric chains, as well as the number, chemical composition, and length of their constituting macroblocks, temperature, pH, and ionic strength of the environment) [78,89], one can categorize the resulting nano-sized particles (usually with diameters up to 200 nm) into two main groups: micelles and polymersomes (polymeric vesicles) [78,89,90]. The former are small aggregates exhibiting a core-shell structure, in which the hydrophobic blocks of amphiphilic macromolecules are stacked together inside, whereas the hydrophilic parts of LBCPs are directed outside (Figure 4a). On the other hand, the structure of polymersomes resembles that of liposomes: they exist as hollow spheroids, in which the LBCPs’ chains are located at the surface and form an amphiphilic double layer with their hydrophilic blocks sticking out on both sides (Figure 4b). It should be noted that each part of such nanoassemblies makes a distinctive and significant contribution to the overall successful performance of a DDS: the hydrophobic core of the micelle, or the interior of the amphiphilic double layer in a polymersome, is responsible mainly for the drug loading capacity (LC) and its controlled release, whereas hydrophilic corona increase the biocompatibility of the whole system and protect drug molecules from any unfavorable destructive interaction with enzymes, serum proteins, and other constituents present in the bloodstream before a DDS reaches its target site [91,92].

Since the loading of polymeric micelles with drugs is performed via a physical entrapment of the latter within the hydrophobic core of the micelle, one can accomplish it by self-assembling LBCPs in the presence of drug molecules (a bottom-up approach, Figure 4c). This strategy is characterized by simplicity and ease of performance, although several important processing conditions (e.g., temperature, pH, the concentration of reagents) must be carefully controlled. It involves the dissolving of amphiphilic LBCPs and hydrophobic drugs in an appropriate organic solvent, followed by a drop-wise mixing of the resulting solution with the aqueous phase. Usually, water-miscible organic solvents are utilized in this method (e.g., THF [93,94], DMF [95], or DMSO [92,96,97]), and they have to be subsequently separated from the drug-containing micelles, together with the unentrapped drug molecules, by means of dialysis [94,95,96,97,98].

Micelles must be biocompatible and sufficiently resistant to the internal environment of the living organism to transport the chosen drug to its destined location (e.g., tumor cells or pathologically altered tissues). Thus, there has been continuous interest in the development of methodologies for the stabilization, cross-linking, and functionalization of polymer micelles. However, their stability cannot be too high since at the targeted site a DDS has to liberate its therapeutic payload in a strictly controlled manner, thus solely affecting the targeted cells as well as maintaining the concentration of the delivered drug within the optimal therapeutic limits for an appropriate amount of time [91,99]. Moreover, in order to minimize the negative side effects occurring during the application of many drugs (especially those used in cancer treatment), or to protect the therapeutic agents that are otherwise easily destroyed in the bloodstream (e.g., nucleic acids used in gene therapy), efforts have recently been made for the synthesis of DDSs that change their physicochemical and/or structural properties in response to some external stimuli, either physical (e.g., light, ultrasound, or temperature) or chemical factors (e.g., changes in pH values and/or redox potential of the environment) [100].

### 3.1. Smart DDSs Based on Micelles

Generally, there are two types of endogenous stimuli (factors independent of human influence and related to the functioning of a living organism) that have been used to trigger a drug release from smart DDSs in cancer cells or tissues—both of them are connected to the altered metabolism of the target site. For instance, it is well known that the increased glycolytic metabolism occurring in tumor cells (the Warburg effect) produces a larger amount of lactic acid and CO_2_ than that observed during the standard metabolism of healthy cells. Since both compounds are expelled from the cells, their increased concentration leads to the acidification of the extracellular microenvironment of the tumor cells, which exhibits a slightly lower pH value (usually below 6.5) than that observed in blood or healthy tissues (ca. 7.4) [100,101]. Moreover, some organelles inside cells (e.g., endosomes or lysosomes) exhibit even lower pH values (usually 4.5–5.5) [100,102]. Taking this into account, pH is one of the most intensively exploited natural triggers for drug liberation from DDSs.

On the other hand, all living cells combat destructive reactive oxygen species (radicals) by utilizing several natural antioxidants, among which glutathione (GSH) is the most abundant [103,104]. Because of this, the concentration of GSH inside the healthy cells (up to 10 mM) is about 1000 times higher than that in the bloodstream, and in the case of tumor cells it is even higher (at least a few times higher). The obvious increase in the reductive conditions of the intracellular environment can be a trigger for safe drug release directly inside a tumor cell [104].

In order to introduce pH responsiveness into micelle-based DDSs, two main strategies can be applied, both of which are focused on the introduction of a physicochemical imbalance into drug-loaded LBCPs’ micelles leading to their destruction and the release of their therapeutic content in the acidic environment of the tumor cells. This can be performed either via the protonation of functional groups incorporated into LBCP chains, or the acid-catalyzed hydrolytic cleavage of the chemical bonds present in the polymer backbone or pendant groups.

#### 3.1.1. Drug Release Induced by Protonation

This approach has been exploited by several research groups; however, a common feature of their works has been the utilization of tertiary amine groups as proton acceptors and PEG as a hydrophilic block of LBCPs. Wang and Zhang synthesized a series of double hydrophobic triblock copolymers, poly(2-(N,N′-diethylamino)ethyl methacrylate)-b-poly(ethylene glycol)-b-poly(2-(N,N′-diethylamino)ethyl methacrylate) (PDEAEMA-*b*-PEG-*b*-PDEAMA) [93], by conducting the ATRP of 2-(N,N′-diethylamino)ethyl methacrylate (DEAEMA) in the presence of 2-bromoisobutyrate-terminated PEG (Br–PEG–Br, a product of PEG esterification with BIBB) as an ATRP macroinitiator. From this work, it is evident that the elongation of hydrophobic blocks does not increase the cytotoxicity of the blank micelles (even at their highest concentration, in which the viability of the tested cell cultures was above 80%), whereas the critical micelle concentration (CMC) decreases. Moreover, by increasing the number of DEAEMA units, one can increase both the hydrodynamic diameter of the blank or drug-loaded micelles (within the 40–180 nm or 50–220 nm ranges, at the physiological pH of 7.4, respectively), as well as the doxorubicin (DOX) loading contents and entrapment efficiencies (up to ca. 8.1% and 89%, respectively, for the copolymer with the highest molar weight). The pH responsiveness of the investigated DDSs was proven by their faster DOX release observed at the endo-/lysosomal pH conditions (between 65 and 90% at pH = 5.4 in comparison to 25–35% at pH = 7.4). In another study [94], Wang, Zhang, and coworkers showed that the length of the hydrophilic block in PDEAEMA also impedes its micellar properties and DOX uptake: the increase in the PEG block length leads to smaller micelles that exhibit lower DOX loading contents and efficiencies (8.0–6.4% and 86–68%, respectively), as well as cumulative drug release (a decrease from ca. 90% to 70% at the endo-/lysosomal pH).

In order to make PDEAEMA micelles more thermodynamically stable, Chen and coworkers proposed a symmetrical elongation of the hydrophobic block by copolymerizing it with the second hydrophobic monomer. For that purpose, they conducted a sequential ARGET ATRP of DEAEMA with either methyl methacrylate (MMA) [105] or HEMA [106]. This method resulted in a symmetrical, double hydrophobic pentablock copolymer, although in the case of the HEMA-based system, its hydrophilic 2-hydroxyethyl groups required hydrophobization via an amidation reaction with the amine groups of folic acid [106]. The obtained micelles showed comparatively low CMC values (especially the system containing MMA units, 2.4–2.8 mg/L) indicative of their potentially better stability in the bloodstream [105], as well as a larger encapsulation efficiency (e.g., 20–35% for the MMA-based system [105] and 45–48% in the HEMA-based copolymer) [106], although the DOX loading contents were substantially lower (below 25%) in comparison to the previously investigated triblock copolymer PDEAEMA. It should be noted that, at the same length of the second monomer, the copolymers with longer pH-sensitive PDEAEMA blocks exhibited higher values of the DOX loading content and efficiency and formed slightly larger micelles while their CMC values decreased. More importantly, in both cases, the DOX-loaded micelles rapidly released their content at an acidic pH and were characterized by cytotoxicity against tumor cells (a tumor-suppressing effect) close to that of free DOX—for example, after 48 h of incubation with DOX-loaded HEMA-based micelles, the viability of HepG2 tumor cells was reduced to ca. 20%.

An interesting option for the synthesis of DDSs based on PDEAEMA-containing micelles is the process of co-micellization, in which drug-encapsulating nano-assemblies are formed due to the entanglement of two different copolymers. It is especially helpful if the desired building blocks of LBCPs have to be synthesized according to different polymerization mechanisms—in many cases, the separation of such polymerization procedures makes laboratory work less tedious and quicker. An exemplification of this strategy is the research work carried out by Yang and coworkers [98,107], in which the authors described mixed micelles formulated from amphiphilic copolymers obtained via the ARGET ATRP of DEAEMA and ring-opening polymerization (ROP) of ε-caprolactone (ε-CL). In reference [98], DOX-loaded mixed micelles consisting of the mixture of amphiphilic diblock copolymers poly(2-(N,N′-diethylamino)ethyl methacrylate)-*b*-poly(poly(ethylene glycol) methyl ether methacrylate) (PDEAEMA-*b*-PPEGMA) and poly(ε-caprolactone)-*b*-poly (poly(ethylene glycol) methyl ether methacrylate) (PCL-*b*-PPEGMA) were investigated. Both the experimental studies on drug release and its computational simulations employing dissipative particle dynamics (DPD) indicated that mixed micelles exhibited good pH-responsivity: they were very stable at the physiological pH, showing very limited leakage of DOX, whereas, at pH = 5.0, they were characterized by an accelerated release of DOX. Moreover, these co-micelles were completely biocompatible, with no cytotoxicity detected during in vitro tests, and their capability for DOX up-take was high: the estimated DOX loading contents and DOX encapsulation efficiencies were in the 23–31% and 60–91% ranges, respectively, depending on their composition [98]. Another study simplified the topology of the copolymers by utilizing PEG monomethyl ether (MPEG) as a macroinitiator in ε-CL ROP and ARGET ATRP of DEAEMA (after prior esterification of MPEG hydroxyl terminal with BIBB) [107]. The DOX loading contents of these nanosystems (hydrodynamic diameters in the 200–300 nm range) were on a comparable level to their poly(poly(ethylene glycol) methyl ether methacrylate (PPEGMA)-based analogs, whereas they exhibited slightly lower DOX encapsulating efficiencies (values between 21% and 63%). By combining experimental work with DPD simulations, the authors showed that DOX tended to distribute in the mixed inner core formed by poly(ε-caprolactone) (PCL) and pH-sensitive PDEAEMA chains, owing to the hydrophobic interactions; however, as the PCL/PDEAEMA chains of the polymers increased in length, the ability of the micelles for DOX loading decreased, suggesting that protective effect of the hydrophilic corona (shell) also plays a role in ensuring an appropriate LC of this type of DDSs [107].

#### 3.1.2. Drug Release Induced by Acid Bond Cleavage

**Figure 5:**
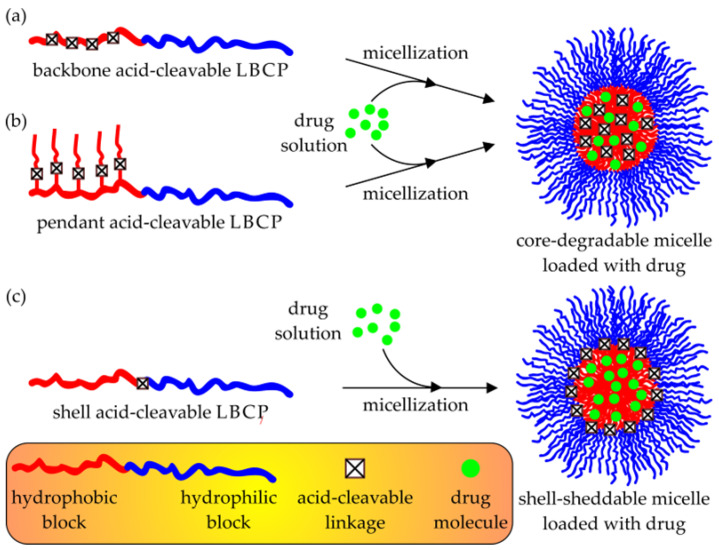
Possible locations of acid-labile linkages within amphiphilic LBCPs before and after micellization: (**a**) backbone acid-cleavable, (**b**) pendant acid-cleavable, and (**c**) shell acid-cleavable nanoassemblies.

Alternatively to the protonation/deprotonation mechanism, pH-responsive polymeric micelles can be disintegrated via the acid-promoted cleavage of the chemical bonds incorporated into their structure with ester, ortho ester, β-thiopropionate ester, hydrazone, imine (e.g., benzoic imine), acetal, ketal, oxime, vinyl ether, or amide groups [108,109,110,111]. Based on the location of these acid-labile linkages within block copolymer chains, the latter (as well as nano-assemblies formed by them in aqueous solutions) can be categorized into three main groups (see Figure 5): backbone acid-cleavable (i.e., those characterized by bond breaking between monomeric units of the hydrophobic block), pendant acid-cleavable (i.e., LBCPs whose hydrophobic blocks are fabricated from monomers bearing acid-labile substituents), and shell acid-cleavable (shell-sheddable, i.e., those characterized by bond breaking at the junction between their hydrophilic and hydrophobic blocks) [109].

Since all ATRP techniques produce new hydrolytically stable C–C bonds between monomeric units, they have no use in the formulation of backbone acid-cleavable micelles. Only an extension of ATRP, namely atom transfer radical polyaddition (ATRPA), has been able to show some results in this field. Li and coworkers proved that biodegradable pH-responsive polyester showing a low critical solution temperature (around 37 °C) could be obtained via the ATRPA of bis(styrenic)- and bis(bromoisobutyrate)-type monomers. As expected, the synthesized copolymer underwent depolymerization at a pH of around 5.5 (due to the acidolysis of ketal bonds incorporated via bis(bromoisobutyrate)-type monomer); however, no drug release tests have been conducted for this system [112].

The concept of a controlled drug release in the case of block copolymers having pendant groups prone to acid-triggered bond breaking is based on the fact that, by changing their chemical composition (due to the cleavage of the side groups), one can disrupt a delicate balance in the hydrophilic/hydrophobic interactions responsible for keeping the micelles intact, thus leading to the destabilization and disassembling of the latter. Such a process is especially promoted if bond breaking increases hydrophilicity or results in the formation of electrically charged moieties within the hydrophobic core of the micelle. Two main synthetic strategies can be applied for the synthesis of this type of copolymers: a direct polymerization or a chemical modification of the reactive side groups present in the already synthesized polymeric chains [109,110]. The latter strategy is mostly used for the attachment of drugs to polymeric carriers having different molecular structures, resulting in the formation of polymer–drug conjugates; thus, it will be discussed in detail in a separate section of this review. The direct polymerization approach usually requires the design and subsequent synthesis of the appropriate monomers bearing the acid-labile functional groups and exhibiting a sufficiently high reactivity in the chosen type of polymerization reactions. For that purpose, as far as ATRP techniques are considered, three synthetic pathways can be utilized: (a) the polymerization of a functionalized, hydrophobic, unsaturated monomer in the presence of a hydrophilic ATRP macroinitiator; (b) the polymerization of a functionalized, hydrophilic, unsaturated monomer in the presence of a hydrophobic ATRP macroinitiator, or the copolymerization of the appropriately functionalized hydrophilic and hydrophobic unsaturated monomers. It should be noted that only the first of these leads to micelles being formed by amphiphilic LBCPs, as shown by the results obtained by Li and coworkers, who used 2-chloropropionate-ended MPEG chains to initiate the ATRP of N-substituted acrylamide (trans-N-(2-ethoxy-1,3-dioxan-5-yl) acrylamide, tNEA) and synthesized a diblock copolymer-containing ortho ester moiety in the pendant substituents [113]. The authors proved that by changing the degree of polymerization within the hydrophobic poly(trans-N-(2-ethoxy-1,3-dioxan-5-yl) acrylamide) (PtNEA) block, one can easily alter the self-assembling properties of the resulting copolymer and obtain nanostructures with morphologies ranging from spherical micelles (for the shortest PtNEA blocks) and rod-like clusters to polymersomes (for the longest PtNEA blocks). Interestingly, regardless of their morphological features, these nanoassemblies were stable at physiological pH but underwent disintegration at the endo-/lysosomal mildly acidic environment (pH 4.6–5.0), with micelles being the most prone to hydrolysis. Moreover, they all were capable of DOX loading and displayed pH-dependent drug release profiles, as well as concentration-dependent cytotoxicity against HepG2 tumor cells [113].

The other two ATRP-based strategies leading to copolymers with labile pendant groups were explored by Wei and coworkers [114], as well as Oh and Khorsand [115]; however, they both resulted in graft copolymers, which subsequently self-assembled into drug-loading micelles. The first group polymerized a newly synthesized hydrophilic methacrylate monomer (a derivative of HEMA acetalized with MPEG oligomers) using a hydrophobic bromine-terminated PCL, a product of the ring-opening polymerization of ε-CL initiated with 2-hydroxyethyl 2′-bromoisobutyrate as the ATRP macroinitiator [114], whereas the second group sequentially copolymerized hydrophilic poly(ethylene glycol) methyl ether methacrylate (PEGMA) with pH-responsive hydrophobic tert-butyl methacrylate using a small-molecule ATRP initiator (2-hydroxyethyl 2′-bromoisobutyrate) [115].

In the last decade, new concepts regarding the utilization of ATRP in the synthesis of copolymers with pH-cleavable pendant substituents have been proposed. Although some of them do not relate to amphiphilic LBCPs (e.g., the usage of deactivation-enhanced ATRP conditions and the acetal-containing diacrylate monomer for the in situ synthesis of an amphiphilic hyperbranched copolymer capable of DOX loading and subsequent releasing at the endosomal pH) [116], the others are directly concerned with such copolymers. For example, Li and coworkers observed that ATRP of a mixture of HEMA and DEAEMA, followed by the reaction between the hydroxyl groups of poly(2-hydroxyethyl methacrylate) (PHEMA) blocks with 2-ethylidene-4-methyl-1,3-dioxolane, resulted in a triblock LBCP, in which at a pH of ca. 5, the protonation of the tertiary amine groups from the PDEAEMA units promoted the hydrolysis of the surrounding pendant ortho ester moieties. Depending on the content of PDEAEMA, their hydrolysis half-times ranged from hundreds of minutes at a pH of 5.4 to several days at physiological pH, but the copolymer with the highest amount of the tertiary amine units was characterized by the most accelerated loss of ortho ester groups [117]. It should be noted that a similar synergistic behavior, including an accelerated DOX release at an acidic medium, was also observed by Dong and coworkers in the case of the related copolymer exhibiting a more complicated, multi-grafted molecular architecture, which was obtained via ATRP of a mixture of 2-(N,N′-dimethylamino)ethyl methacrylate and (2,2′-dimethyl-1,3-dioxolane-4-yl)methyl methacrylate carried out in the presence of a polymeric macroinitiator bearing pendant 2-bromoisobutyrate groups (a triblock ATRP macroinitiator obtained in the course of the ROP of ε-CL and γ-(2-bromo-2-methylpropionate)-ε-caprolactone initiated with MPEG) [118].

Shell-sheddable micelles formulated by a third group of the amphiphilic LBCPs prone to pH-triggered bond cleaving have been developed in response to the so-called “PEG dilemma” encountered during the in vivo application of PEGylated drug nanocarriers: their low cellular uptake and an increased possibility of the production of anti-PEG antibodies in the case of the prolonged presence of PEG in the bloodstream [119,120]. In the mildly acidic microenvironment of the tumor cells and tissues, these micelles lose their PEG coronas due to the cleavage of pH-labile bonds, linking hydrophobic and hydrophilic blocks of the amphiphilic LBCPs, whereas their hydrophobic cores undergo aggregation at the cellular walls. Although many pH-breakable bonds can be utilized for that purpose, in the case of the ATRP-derived shell-sheddable amphiphilic LBCPs, only two types have been recently used: acetal and imine linkages [121,122]. They can be incorporated into the copolymer chain structure by the chemical conjugation of two different homopolymers bearing reactive chain-ends [121,122] or the direct polymerization of the unsaturated monomer started with the appropriately functionalized ATRP macroinitiator [123]. A good example of the first approach is the work of Patil and Wandgaonkar [121], who reported the synthesis and self-assembling properties of an acetal-linked diblock copolymer of ε-CL and N-isopropylacrylamide (NIPAM) PCL-*b*-PNIPAM. This copolymer was formed through the alkyne-azide click reaction between the propargyl-terminated PCL and azide-ended poly(N-isopropylacrylamide) (PNIPAM) chains. The acetal linkers were introduced to the PNIPAM blocks via the ATRP of NIPAM carried out with the usage of a newly designed ATRP initiator, namely 2-(1-(2-azidoethoxy)ethoxy)ethyl 2-bromo-2-methylpropanoate. At room temperature, the resulting copolymer self-assembled in an aqueous solution into micelles (ca. 74 nm in diameter) that could encapsulate rhodamine B (as a model drug) at a pH of 7.4 and subsequently release it at the endosomal pH or upon heating up to 40 °C. A very similar approach was tested by Ni and coworkers [124], who conjugated the acetal-containing coumarin- and azide-terminated hydrophobic chains of PCL, with the hydrophilic monoalykynyl-terminated graft block copolymer of PEGMA and dimethylaminoethyl methacrylate (DMAEMA), synthesized via ATRP in the presence of a propargyl 2-bromoisobutyrate initiator. This linear-graft triblock copolymer showed good DOX loading content (5.3%), DOX loading efficiency (28.5%), and increased DOX release at a pH of 5.0; additionally, it was capable of simultaneous nucleic acid loading and subsequent delivery into HeLa tumor cells.

An interesting option for the application of click chemistry in the synthesis of the shell-sheddable amphiphilic LBCPs is the in situ formation of the pH-labile junction during the final step of conjugation of the copolymer building blocks. However, this approach requires the presence of the appropriately selected reactive groups at the terminals of (co)polymers subjected to conjugation. Dimitrov and coworkers tested it on the shell-sheddable micelles formulated by a copolymer containing a benzyl imine moiety, as well as PEG, PDMAEMA, and polylactide (PLA) blocks [122]. The simultaneous formation of both the imine linker and final copolymer chains proceeded in the mixture of monoamine-terminated DMAEMA/lactide (LA) diblock copolymer and PEG homopolymer terminated with benzaldehyde group, without the use of any catalyst. In this study, the ATRP of DMAEMA was conducted on the monoalkynyl-terminated brominated polyester macroinitiator (A-PLA-PDMAEMA) resulting from the ROP of LA (initiated with propargyl alcohol) and subsequent esterification with BIBB. Interestingly, in earlier work, Dimitrov and coworkers also showed that A-PLA-PDMAEMA itself formed micelles in aqueous media that were capable of the delivery and controlled release of curcumin inside acute promyelocyte leukemia-derived HL-60 cells [125]. It is worth noting that the research works of Dimitrov’s group cited above are indicative of a new trend emerging in the field of the micelle-based DDSs [122,125], namely, the incorporation of some precisely designed subcellular targeting ligands into amphiphilic LBCP chains. Dimitrov and coworkers explored this by introducing (via a post-polymerization reaction with (4-bromobutyl)triphenylphosphonium bromide) pendant triphenylphosphonium and quaternary ammonium cations, which facilitated the transportation of the dePEGylated copolymer through the phospholipid barrier of the cellular and lysosomal walls.

**Figure 6:**
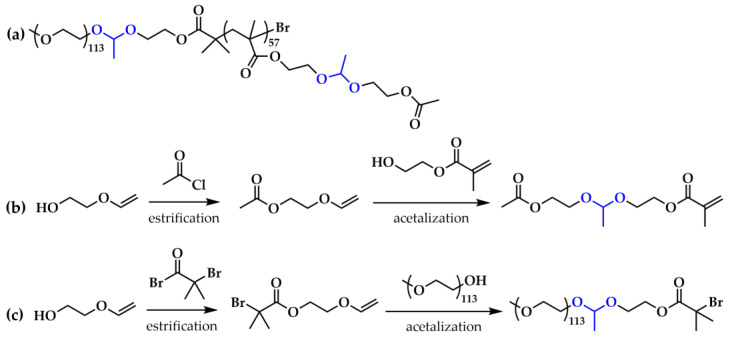
(**a**) Structure of a dual location acid-cleavable amphiphilic LBCP containing pH-labile acetal groups, as well as synthetic pathways, resulting in the preparation of (**b**) ATRP monomer and (**c**) ATRP macroinitiator used for its synthesis [123].

Recently, several research groups have investigated an interesting option of combining in one DDS two different mechanisms of pH-dependent micelle destabilization and/or drug release. Oh and coworkers utilized a newly designed ATRP macroinitiator (a product of ethylene glycol vinyl ether (EGVE) esterification with BIBB followed by acetalization with a hydroxyl-terminated MPEG) and a methacrylate monomer bearing a pendant acetaldehyde acetal linkage (a product of EGVE esterification with acetyl chloride followed by acetalization with HEMA) for the synthesis of a dual location acid-cleavable amphiphilic LBCP (Figure 6) [123]. This copolymer was characterized by the presence of pH-labile acetal linkers both in the pendant groups of the hydrophobic block, as well as at the hydrophobic/hydrophilic block junction. Therefore, its nano-assemblies in aqueous media were prone to both corona detachment (upon cleavage of the acetal block junction) and core destruction (via the cleavage of the pendant acetal moieties). It is believed that such a combination of these two micelle destabilization mechanisms may allow us to overcome some of their limitations, e.g., the sluggish degradation of the core-degradable systems and undesired aggregation of dePEGylated cores for shell-sheddable systems. Moreover, the authors further enhanced the DOX loading capability of this system as well as the acid-catalyzed hydrolysis of the acetal moieties by the copolymerization of additional monomer-bearing acid-ionizable imidazole groups [123].

Zhang and coworkers explored the concept of a dual mechanism of drug encapsulation in the case of micelles formulated from a double hydrophilic triblock glycopolymer containing MPEG and poly(2-gluconamidoethyl methacrylate) (PGAMA) as the hydrophilic end-blocks, separated by a hydrophobic PDEAEMA block [97]. For that purpose, a sequential ATRP of DEAEMA (first step) and 2-gluconamidoethyl methacrylate (GAMA) (second step) was initiated with the BIBB-modified MPEG macroinitiator. For micellization carried out in the presence of boron-containing anticancer drug bortezomib (BTZ), they obtained BTZ-loaded micelles with hydrodynamic diameters of ca. 80 nm and a rather high value of CMC (30 mg/L), in which BTZ entrapment was achieved by both hydrophobic interactions with the PDEAEMA core and the covalent complexation (conjugation) of BTZ boronic acid functionality with glucose groups of PGAMA. The achieved BTZ loading content and entrapment efficiency were estimated to be 7.6% and 72%, respectively. At physiological pH, the BTZ release from these micelles was very slow (ca. 20% after 10 h), whereas it increased substantially (up to ca. 60% after 10 h) when the endo-/lysosomal pH conditions were applied. In addition, this DDS showed an appreciable prolonged release profile, since even after 60 h, the cumulative release of BTZ did not exceed 70%.

Zeng and coworkers verified the applicability of the pH-dependent double-triggered drug release strategy during combination anticancer therapy [96]. Starting from the Br-containing ATRP macroinitiator (synthesized from MPEG, 4-formylbenzoic acid, and BIBB), they polymerized the conjugate of HEMA and ibuprofen into a hydrophobic block of amphiphilic LBCP. In an aqueous environment and at physiological pH, the obtained diblock copolymer easily formed spherical micelles, with mean hydrodynamic diameters around 200 nm and a small CMC value of 2.5 mg/L, which could be filled with DOX molecules and exhibited a LC and encapsulation efficiency of ca. 10% and 33%, respectively. Under endo-/lyposomal acidic conditions, these micelles disintegrated due to the cleavage of the benzoic imine bonds linking their hydrophobic cores with MPEG hydrophilic shells (thus they belong to the group of shell-sheddable nano-assemblies), which was accompanied by the hydrolysis of the ester bonds connecting the ibuprofen to the HEMA-derived monomeric units. Although both drugs (DOX and ibuprofen) were released from micelles at sufficiently high rates, each process was controlled by different factors: the DOX release depended solely on micelle collapse, while the ibuprofen release was additionally controlled by ester bond hydrolysis. It should be noted that the synthesized DDS exhibited an anti-tumor behavior against B16 murine melanoma cells similar to the free DOX hydrochloride, as evidenced by both in vitro and in vivo tests.

#### 3.1.3. Redox and Dual Redox/pH-Sensitive Systems

Changes in the redox conditions in the microenvironment of tumor cells are another important factor that is used as endogenous stimuli for targeted drug delivery. Up to now, several types of oxidation- and/or reduction-sensitive chemical functionalities have been proposed for that purpose [126,127,128], among which two have gained particular popularity in the case of ATRP-derived polymeric micelles: arylboronate moiety (for an oxidation-responsive DDS) and disulfide (SS) bond (for a reduction-responsive DDS).

##### Boronate-Bearing Oxidation-Responsive Systems

**Figure 7:**
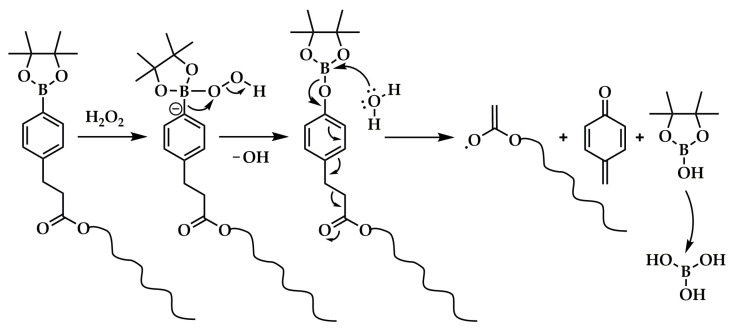
Mechanism of arylboronate oxidation under the influence of hydrogen peroxide.

Li and coworkers utilized an ATRP macroinitiator, MPEG esterified with a proper acyl halide (2-chloropropionate chloride or BIBB) [129,130], to copolymerize a phenylboronic pinacol ester-containing acrylate monomer (4-(4,4,5,5-tetramethyl-1,3,2-dioxaborolan-2-yl)benzyl acrylate) with NIPAM [129], or fluorescent acrylic acid ester bearing 1,8-naphthalimide groups [130]. In both cases, an amphiphilic LBCP was produced, which at 37 °C easily formed micellar systems in aqueous media (a double hydrophilic copolymer containing NIPAM exhibited a critical aggregation temperature between ca. 10 °C and 20 °C, which depended on its molecular weight) [129,130]. The subsequent loading with a hydrophobic DOX resulted in the micellar DDSs, which were susceptible to a well-known arylboronate oxidation mechanism (Figure 7) and underwent destabilization under the influence of H_2_O_2_ or the intracellular reactive oxygen species (ROS) [131].

In another study, Li’s group showed that arylboronate oxidation chemistry could be effectively combined with the pH-induced destruction of LBCP micelles. Starting with the BIBB-esterified MPEG, they carried out a simultaneous ATRP, obtaining an amphiphilic triblock copolymer, in which the hydrophobic block contained monomeric units of the aforementioned oxidation-sensitive arylboronate monomer and another acrylate bearing the pH-labile ortho ester substituents [132]. The results of the pH- and/or H_2_O_2_-triggered degradation of the Nile red (NR)-loaded micelles showed that the oxidation of the phenylboronic ester moieties promoted the subsequent hydrolysis of the ortho ester pendant groups (due to a catalytic effect of the newly formed pendant carboxylic groups). Moreover, the kinetics of both these reactions could be easily tuned by changing the copolymer composition and oxidant concentration, as well as the pH of the environment.

##### Disulfide-Bearing Reduction-Responsive Systems

**Figure 8:**
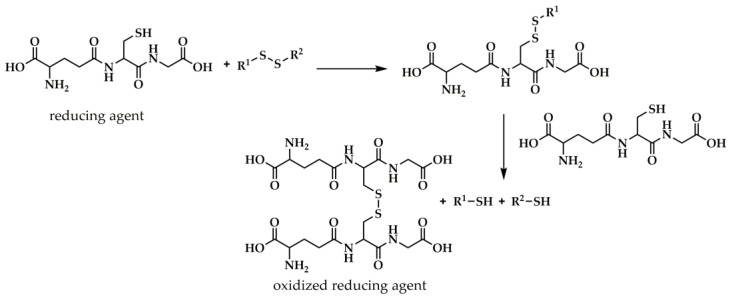
Mechanism of the reductive cleavage of disulfide bonds under the impact of the thiol-containing glutathione.

The principle of operation of the SS-containing reduction-responsive LBCPs is based on a well-known two-step thiol-disulfide exchange reaction proceeding under the influence of a suitable reducing agent bearing thiol groups (e.g., intracellular glutathione). First, one molecule of the reducing agent cleaves the SS bond, resulting in the formation of a mixed SS moiety and the liberation of a free sulfhydryl group. In the next step, another molecule of reductant breaks the mixed SS bond, releasing the second sulfhydryl group, while dimerizing to the oxidized form of the reducing agent (Figure 8) [133].

Similarly to the acid-cleavable groups of the pH-responsive amphiphilic copolymers, the reduction-cleavable SS linkages can be incorporated into three different locations within LBCP macromolecules, i.e., in the hydrophobic block backbone (resulting in the core-cleavable micelles), at the junction between the hydrophobic and hydrophilic blocks (leading to the shell-sheddable micelles), or as components of the pendant groups attached to the hydrophobic block [134]. However, in the case of the ATRP-derived LBCP micellar DDSs, only the last two of these have any significance. In fact, the backbone SS multi-cleavable drug-delivering nanoassemblies are usually produced via step-growth (e.g., hydroxyl-carboxylic or disulfide-thiol polycondensation, and a combination of Michael-type polyaddition with the “click” chemistry-based post-polymerization modification) or chain-growth polymerization techniques other than ATRP (e.g., ROP and RAFT) [134], whereas the usage of ATRP is limited to the processes in which the SS-containing ATRP macroinitiator is utilized for the polymerization of PEGMA, thus resulting in a copolymer with a non-linear topology [134,135,136].

LBCPs with SS linkage between their blocks can be synthesized in the course of ATRP started by a properly designed SS-containing hydrophobic or hydrophilic ATRP macroinitiator. Wang and coworkers utilized bis(2-hydroxyethyl) disulfide (HO-SS-OH) as an initiator for the ROP of ε-CL and esterified the resulting SS-containing hydroxyl-terminated PCL with BIBB, thus obtaining an SS-containing hydrophobic macroinitiator [137]. The latter was applied in the ATRP of *tert*-butyl methacrylate (tBuMA), producing a symmetrical hydrophobic tetrablock copolymer, which, upon the subsequent hydrolysis of the *tert*-butyl ester groups with trifluoroacetic acid, formed a desired amphiphilic LBCP composed of two symmetrical PCL-*b*-poly(methacrylic acid) blocks separated via a single SS linkage. Interestingly, Wang’s group also demonstrated that a simple change in the order of the ROP and BIBB esterification steps could cause LBCP to exhibit a different topology (i.e., a classic amphiphilic diblock copolymer), even when using the same reagents (HO-SS-OH, ε-CL, and tBuMA). Via a reaction of HO-SS-OH with an equimolar amount of BIBB, they synthesized a bifunctional initiator active both in the ATRP and ROP of cyclic ester, namely 2-hydroxyethyl-2′-(bromoisobutyryl)ethyl disulfide (HO-SS-Br), and then separately polymerized ε-CL and tBuMA [138]. The micelles formulated from both these copolymers showed very good paclitaxel-loading properties (exhibited by the hydrophobic PCL cores) [137,138], whereas diblock copolymer-containing micelles could also encapsulate amine-containing cisplatin via electrostatic interactions with its carboxylic groups located in the hydrophilic shell [138]. Drug release profiles proved that in a reductive microenvironment, these DDSs showed an accelerated release of drug molecules, while in vitro tests also indicated their increased cellular up-take and cytotoxicity to a non-small-cell lung cancer CRL-5802 cell line, even when compared to the free drugs [137,138].

Separately, Huang’s group and Oh’s group synthesized a PLA-based hydrophobic ATRP macroinitiator (PLA-Br) via the ROP of racemic D,L-LA initiated by HO-SS-Br [139,140]. Subsequently, PLA-Br was utilized in the ATRP of methacrylate monomers bearing pH-sensitive amine groups: 2-aminoethyl methacrylate (AEMA) [139] or DMAEMA [140]. In both cases, the final step of amphiphilic LBCP synthesis included a post-polymerization chemical modification of the pendant amine groups aimed at the formation of cations (via the quaternization of DMAEMA units with methyl iodide) [140] or anions (via the amidation of AEMA units with dicarboxylic acid cyclic anhydrides) [139]. On one hand, the incorporation of ionic moieties within the micelles’ shells made them more hydrophilic, but also endowed them with some additional features: increased stability and resistance to protein-fouling during circulation in the bloodstream (a case of nano-assemblies with polyanionic shells) [139] or the capability to form polyplexes with oligonucleotides used in the gene therapy (a case of the polycationic nanocarriers) [140], without compromising the ability of the micelles’ hydrophobic cores to DOX encapsulation. It is worth noting that a shell charge of the micelles studied by Huang and coworkers showed an interesting dynamic dependence on the pH of the microenvironment: though negative at physiological pH, it became positive at the more acidic tumor site and thus improved the up-take of the drug-loaded micelles by tumor cells (cationic species are more prone to endocytosis) [139].

The use of the PEG-based hydrophilic ATRP macroinitiator in the synthesis of reduction-susceptible shell-sheddable LBCP micelles was reported by Oh’s group [141,142]. MPEG activated with 1,1′-carbonyldiimidazole was esterified with HO-SS-Br via a carbonate linkage and then served as a macroinitiator in the ATRP of hydrophobic unsaturated monomers [141]. Model studies carried out for styrene ATRP showed that the resulting amphiphilic LBCPs self-assembled in aqueous media into nanostructures with different morphologies (micelles, vesicles, or rod-like particles), which depended on both the structure of copolymer chains and processing conditions [141]. In another study, the same general procedure was applied for the preparation of DOX-loaded micelles, albeit styrene was replaced with a more hydrophobic methacrylate monomer derived from rosin, namely dehydroabietic ethyl methacrylate [142]. In vitro tests showed that this type of DDS was characterized by a good stability toward proteins, increased cellular uptake, and promptly released DOX in contact with reductive GSH upon internalization into HeLa tumor cells (a cumulative DOX release after 24 h of incubation changed from ca. 10% to almost 50% when the concentration of GSH changed from 0% to 10 mM) [142].

The third class of SS-cleavable LBCP micellar DDSs comprises copolymers, in which hydrophobic polymeric blocks contain pendant substituents bearing SS bonds. Similarly to their pH-sensitive analogs, this type of drug-loaded micelles delivers their therapeutic cargo as a result of the disturbance of their hydrophilic/hydrophobic balance occurring upon a reductive breaking of SS bonds at the target site. This type of LBCPs is easily produced by a direct ATRP approach using MPEG-Br, as a hydrophilic macroinitiator, and the SS-containing methacrylate monomer. One of the most commonly used monomers for that purpose is the product of a two-step Steglich esterification of 3,3′-dithiodipropionic acid with HEMA and ethanol (HMSSEt) [143,144]. Oh and coworkers used this approach for the preparation of the micelle-forming diblock copolymer MPEG-*b*-PHMSSEt and reported its good encapsulation and drug-releasing properties toward NR (as a simple drug-modeling molecule) [143], as well as antitumor DOX [144]. A DOX-loaded system also exhibited desirable cytotoxicity toward HeLa cancer cells, comparable with free DOX molecules [144].

**Figure 9:**
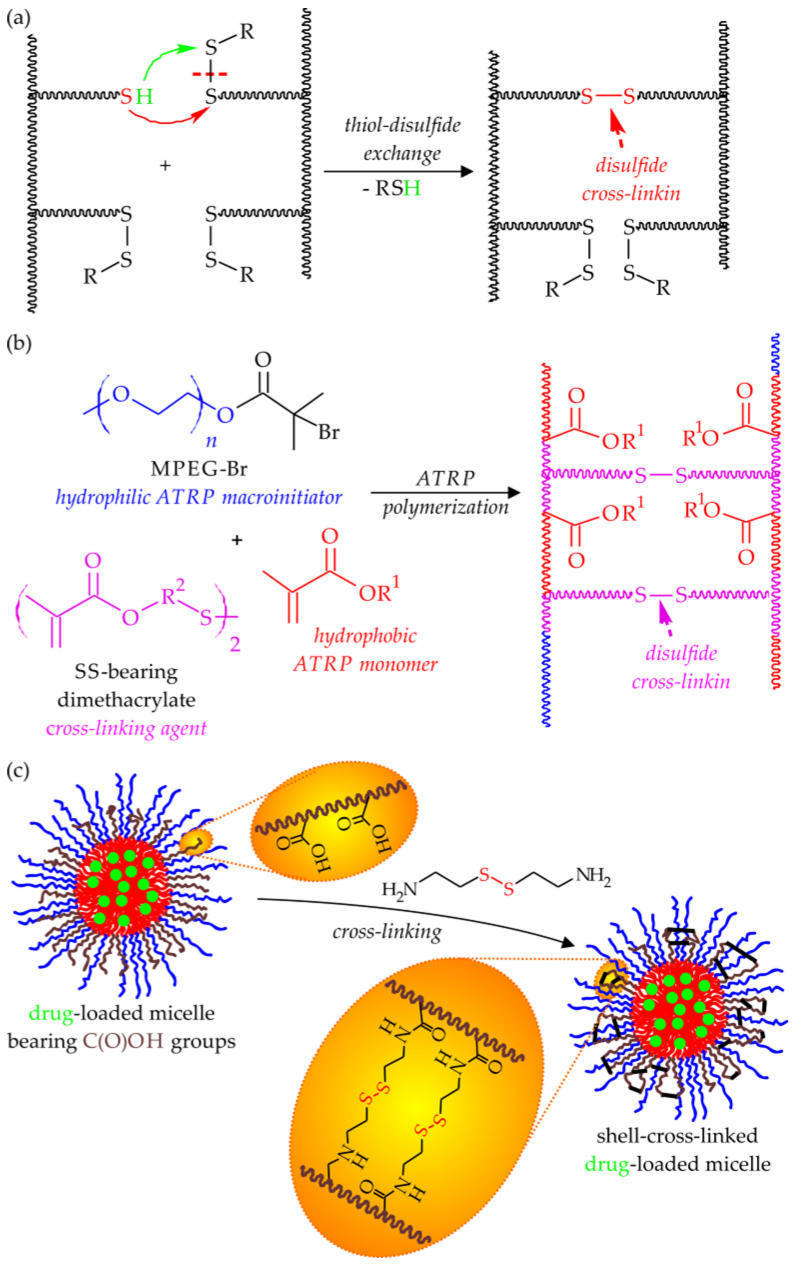
Different synthetic strategies of the SS-based chemical cross-linking of the micellar DDSs: (**a**) reductive cross-linking of the pendant SS-bearing groups, (**b**) cross-linking via a direct ATRP (co)polymerization, (**c**) cross-linking via a post-polymerization modification.

Although LBCPs with SS-containing pendant groups can themselves be used in the formulation of the micellar DDSs, another attractive possibility of their application has been also investigated in recent years—the synthesis of micelles with cross-linked cores. Such systems have been proposed as a way to circumvent the problems associated with CMC values that are too high, exhibited by many classical (non-cross-linked) micellar DDSs, which experience instability upon dilution (i.e., during circulation in the bloodstream). Oh and coworkers explored this idea in the case of a symmetrical double hydrophobic triblock copolymer consisting of the PEG hydrophilic central block and two terminal hydrophobic PHMSSEt blocks [95]. The authors observed that by subjecting PHMSSEt-*b*-PEG-*b*-PHMSSEt micelles to a catalytic amount of a reductant (e.g., D,L-dithiothreitol, DTT), one could cleave only a small number of SS moieties—too few to lead to the disassembling of the micelles. Instead, the newly formed sulfhydryl groups attached to one copolymer chain acted as reductants in the thiol–disulfide exchange reaction with the SS groups of the adjacent chains, thereby resulting in the formation of the covalent cross-links between those chains (see Figure 9a). Differential light scattering (DLS) measurements showed that when subjected to an excess of solvent, these cross-linked micellar systems increased their sizes (an effect of core swelling), contrary to the non-cross-linked analogs experiencing disassembling. It should be noted that both in vitro and in vivo tests proved the complete degradation of the SS-cross-linked cores of the DOX-loaded micelles upon contact with intracellular concentrations of GSH, although they released their payloads a little bit slower than their non-cross-linked counterparts [95].

Interestingly, the cross-linking of the ATRP-derived LBCP micelles via reduction-degradable SS bonds can be achieved by methods other than the one described above. For example, Chhikara and coworkers utilized the inverse miniemulsion AGET ATRP technique to copolymerize DMAEMA with a newly synthesized SS cross-linker (a symmetrical dimethacrylate-containing short PEG blocks linked via SS bonds), in the presence of an MPEG-Br initiator (Figure 9b). After loading with DOX, they obtained nanoassemblies responding to both the changes in the concentration of GSH and the pH of tumor tissues, which were also cytotoxic toward HeLa cells [145]. A similar strategy, utilizing direct polymerization for core-cross-linking of the micelles, was described by Liu and coworkers [146]. First, they synthesized an amphiphilic, bromine-terminated diblock copolymer (via ATRP of tBuA initiated with MPEG-Br) and then used it as an ATRP macroinitiator to copolymerize tBuA and SS-bearing diacrylate cross-linking agent, namely *N*,*N*’-bis(acryloyl)cystamine. The resulting core-cross-linked micelles, after additional hydrophilization (i.e., acidolysis of *tert*-butyl side groups), could easily encapsulate hydrophobic DOX molecules and subsequently release them in a controlled manner when triggered by the acidification of the environment and an increase in the concentration of GSH. Their cytotoxicity toward HeLa tumor cells was on a comparable level (e.g., ca. 40% cell viability at a dosage of 10 μg/mL) with that of a free DOX [146].

A different approach was proposed by Petrov and coworkers, who investigated the post-polymerization cross-linking of mixed co-micelles composed of two double hydrophilic symmetrical triblock copolymers containing hydrophobic PCL as their central block [126]. One of these copolymers contained long hydrophilic PEG blocks (PEG-*b*-PCL-*b*-PEG), whereas the other one, PAA-*b*-PCL-*b*-PAA, contained much shorter hydrophilic segments of PAA and was obtained via the ATRP of tBuA initiated on both chain-ends of the brominated PCL, followed by the acidolysis of the ester moieties to free carboxylic groups. The co-micellization of these two copolymers resulted in nanoassemblies composed of a common PCL core and a mixed, bilayer, hydrophilic shell containing PEG and PAA blocks of different lengths. After loading with caffeic acid phenethyl ester (CAPE), the hydrophilic coronas of these micelles were cross-linked via the introduction of cystamine dichloride and its reaction (i.e., amidation) with the carboxylic groups of PAA segments (Figure 9c). Because of the difference in the length of PEG and PAA segments, the cross-linking reaction took place in the inner layer of the micelles’ hydrophilic coronas and stabilized the hydrophobic core without comprising its ability to encapsulate CAPE. In fact, under simulated physiological conditions, no CAPE leakage was detected; however, at mildly acidic pH or in a reductive environment, these micellar DDSs quickly released their therapeutic cargo [126].

Since the quick and efficient release of drug molecules from the drug-loaded nanoassemblies internalized by tumor cells is a key factor in the case of smart micellar DDSs, the design and synthesis of micelles that can be disassembled via two (or more) different mechanisms operating simultaneously have seen growing interest in the last decade. This concept was also investigated in the case of reduction-susceptible DDSs. For example, Oh and coworkers reported dual-site redox-responsive micelles formed by a linear diblock copolymer containing MPEG and PHMSSEt blocks [147]. These micelles were susceptible to disintegration not only via the changes in hydrophobicity of the PHMSSEt core (resulting from a GSH-induced cleavage of SS bonds in its pendant groups), but also due to a detachment of the MPEG hydrophilic corona caused by the breaking of the SS bond at the block junction. In order to synthesize this new type of shell-sheddable/core-degradable LBCP, the authors utilized the ARGET ATRP technique and the SS-containing bromine-ended MPEG macroinitiator (a product of MPEG esterification with HO-SS-Br) to copolymerize HMSSEt. The authors also showed that these micelles loaded earlier with DOX experienced no drug leakage at the non-reductive conditions, whereas in the presence of any reductant, they very quickly released their payload (e.g., up to 70% of the encapsulated NR indicator after ca. 5 h of incubation in the reductive environment). During in vitro tests, they also exhibited a similar profile of cytotoxicity toward HeLa tumor cells as the free DOX [147]. Recently, the same research group has optimized the processing of the abovementioned micellar DDS by introducing an interesting concept: the so-called “lab-on-chip” flow synthesis of micelles in a two-phase microfluidic reactor [148,149].

The concept of an enhanced drug release through the synergistic effect of two micelle destabilization mechanisms was further extended to the joint action of two endogenous stimuli. The correctness of this strategy is proven in the study which Liu’s group reported, that in the presence of GSH, the cystamine-cross-linked micelles containing pH-sensitive PAA segments showed two to three times more accelerated DOX release at a pH of 5.0 compared to non-reducing conditions [146]. Several other research groups have applied a direct polymerization approach in the synthesis of this type of pH/redox dual stimuli-responsive micellar systems. Oh and Jazani obtained acid shell-sheddable pendant SS-cleavable micelles via a multistep synthetic procedure: starting from MPEG and ethanolamine, they synthesized ketal-containing amine-capped MPEG, which, upon amidization with BIBB, resulted in the macroinitiator utilized in a subsequent step of the ATRP of HMSSEt. Thus, a diblock copolymer was obtained, consisting of a hydrophilic MPEG segment linked through a pH-labile ketal moiety to a hydrophobic polymethacrylate block having multiple redox-responsive SS pendant groups [150]. Oh’s research group also reported on a more sophisticated redox-responsive amphiphilic LBCP additionally equipped with two types of pH-sensitive sites: an acid-cleavable acetal group located at the junction of their hydrophilic and hydrophobic blocks as well as the DMAEMA units, which are easily protonated in acidic media [151]. This copolymer was produced via the ARGET ATRP of HMSSEt, starting from an acetal- and bromine-bearing bifunctional MPEG macroinitiator, and formed colloidally stable nano-sized micelles (ca. 83 nm in diameter), whose hydrophobic cores were additionally cross-linked under reducing conditions. Interestingly, due to the presence of dimethylamine moieties, the obtained dual-location dual pH/reduction-degradable micelles were capable of pH-reversible nucleic acid complexing, potentially useful in a gene therapy [151]. This type of gene-delivering system, namely polyplexes, will be discussed in a separate section of this review.

#### 3.1.4. Micelles Responsive to External Stimuli

In order to increase the efficiency of drug release and to accelerate polymer degradation, dual- and multi-responsive DDSs, utilizing external stimuli, have been repeatedly highlighted. Of the possible stimuli, thermosensitive systems are advantageous for clinical applications, as several spatial heating systems, such as focused high-intensity ultrasound, are already used to treat tumors. The temperature-induced change in micellar function can be designed using hydrophilic blocks that become hydrophobic above the lower critical solution temperature (LCST), tuned to the local body temperature. Below the LCST, these blocks are located in the micelle corona, while above this temperature, they move to the hydrophobic core, which promotes the release of the drug molecules stored there. NIPAM copolymers are most commonly used to prepare thermosensitive blocks. Still, the additional hydrophilic segments must be introduced into the micelle corona to suppress the formation of large intercellular aggregates above the LSCT. For the synthesis of such micelles, the RAFT method is mainly used [152], but in the following section, we will show examples of carriers obtained using ATRP. For example, it was proven that micelles formed by the copolymer poly(ethylene glycol)-*ss*-(poly(dimethylaminoethyl methacrylate)-*co*-poly(2-nitrobenzyl methacrylate)) (PEG-*ss*-(PDMAEMA-*co*-PNBM)) could respond to various stimuli, such as pH, dithiothreitol (DTT), temperature, and UV light irradiation [153]. In the presented study, NR was used as a hydrophobic model drug. Using a single stimuli trigger, the NR release was 28% when the temperature was increased to 50 °C, 40% after 10 mM of DTT addition, 80% at basic conditions (pH = 11), and 89% in 30 min when the UV light irradiation was applied. However, the highest cumulative release was achieved when the combination of triggers was used, that is, UV irradiation under pH = 11. In that case, the NR release increased up to 93% in 60 min. Other combinations of the triggers, that is, UV irradiation under pH = 11 with a reductant and pH trigger with a reductant, did not result in an increase in the release rate. The combination of UV irradiation with 10 mM DDT was suitable for core-crosslinked micelles prepared from amphiphilic block copolymer methoxy poly(ethylene glycol)-*b*-poly(3-azido-2-hydroxy-propyl methacrylate-*co*-*ο*-nitrobenzyl methacrylate) (mPEG-*b*-P(GMA-N_3_-*co*-NBM)) and alkyne-functionalized crosslinking agent containing a disulfide bond in the structure [154]. In that case, the UV irradiation was accelerating the cleavage of disulfide crosslinkers, increasing the release rate after 360 min from 56.9%, which was achieved in the reductive environment, to 73.8%. Similar results were obtained when light irradiation was combined with oxidation. The amount of NR released increased from 53.3% to 76.7%. An interesting example of UV light-breakable and thermosensitive block copolymer poly(2-nitrobenzyl methacrylate)-*b*-poly(2-(2-methoxyethoxy)ethyl methacrylate-*co*-oligo(ethylene glycol) methacrylate) (PNBM-*b*-P(MEO_2_MA-*co*-OEGMA)) was proposed by Yuan and Guo [155]. Under UV irradiation, hydrophobic poly(2-nitrobenzyl methacrylate) (PNBM) was converted into hydrophilic poly(methacrylic acid) (PMA) and the micelles were dissociated. When the solutions were heated, poly(methacrylic acid)-*b*-poly(2-(2-methoxyethoxy)ethyl methacrylate-*co*-oligo(ethylene glycol) methacrylate) (PMA-*b*-P(MEO_2_MA-*co*-OEGMA)) copolymers re-self-assembled into micelles with poly(2-(2-methoxyethoxy)ethyl methacrylate-*co*-oligo(ethylene glycol) methacrylate) (P(MEO_2_MA-*co*-OEGMA)) core and PMA shell. Smart block copolymers have also been synthesized by Jazani and coworkers [156]. A triple stimuli-responsive copolymer exhibited responses to acid, reduction, and light. The preparation of block copolymers included the ATRP of carbonyl imidazole methacrylate in the presence of a difunctional initiator with disulfide bonds and two acetal linkages, followed by the postpolymerization reaction of carbonyl imidazole with an *o*-nitrobenzyl amine. It was proven that UV irradiation caused NR release up to 70% in 10 h; meanwhile, the diffusion of NR was enhanced when both stimuli, 10 mM GSH and pH = 4.2, were simultaneously applied. In that case, the NR release rose from 20% to 90%, compared with that in the experiment when the single stimuli was used.

### 3.2. Smart DDSs Based on Polymersomes

Polymersomes are artificial vesicles made from amphiphilic copolymers, which are more stable than liposomes and show less toxicity in vivo [157,158]. If polymersomes are assembled from polymers capable of carrying and releasing drugs, then they can be used as DDSs. Recent studies have demonstrated that synthesizing polymers and copolymers using ATRP techniques results in better drug delivery performance, such as enhanced colloidal dispersion stability, raised swelling ratios, and responsiveness to a pH change, when compared to polymers synthesized by traditional radical polymerization techniques [159,160]. Polymersomes have many advantages, such as the ability to encapsulate both hydrophobic and hydrophilic substances, good biocompatibility, physical and chemical robustness, high LC, and high colloidal stability, so they are commonly researched as potential DDSs [157,161]. Moreover, they can release drugs in different target sites, depending on environmental conditions.

#### 3.2.1. pH-Triggered Drug Release

**Figure 10:**
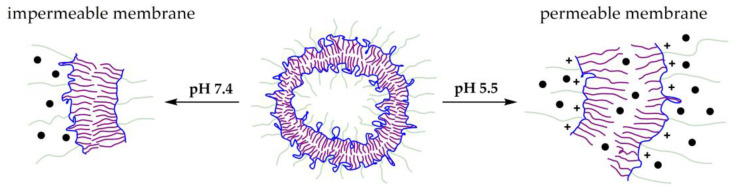
Schematic overview of pH-responsive polymersomes’ membrane behavior in neutral and acidic environments.

Polymersomes release drugs while subjected to external stimuli or a change in the environment, such as a pH change (Figure 10) [162]. Lorella Izzo et al. researched pH-responsive polymersomes that could swell without disaggregation, which significantly lowered their cytotoxicity [163]. They synthesized a three-component amphiphilic copolymer utilizing Br-terminated MPEG as a copolymerization macroinitiator and MMA and DMAEMA as ATRP monomers. The MPEG formed a hydrophilic block, MMA provided hydrophobicity to the poly(methyl methacrylate)-*ran*-poly(dimethylaminoethyl methacrylate) (PMMA-*ran*-PDMAEMA) block (*ran* stands for random distribution of monomers within this block), and DMAEMA was used to trigger the pH-dependent size-change of the polymersomes. Moreover, DMAEMA can form strong hydrogen bonds, hence acting as a non-covalent cross-linker between different polymers forming the vesicle. Both linear and branched copolymers were synthesized, differing in mol% of DMAEMA (22 to 62 mol%) and *M*_n_ of the product (7–51 kDa). Linear polymers with 22–28 mol% of DMAEMA provided the best results. At a pH of 7.4, they were able to form polymersomes with a monomodal size distribution, which suggests that at this pH, no release of copolymers took place, while after the pH was reduced to 4.4, the vesicles increased 10 times in size. These vehicles were loaded with paclitaxel (PTX), releasing only 5–7% of the drug in 48 h under neutral and slightly acidic conditions and 52–41% under acidic conditions in just 2 h. Therefore, it was stated that the systems developed are able to release PTX at lysosomal pH.

#### 3.2.2. Miscellaneous Systems

Polymersomes do not have to be only pH-sensitive. Jianzhong Du et al. developed both a pH- and ultrasound-responsive system, utilizing Br-terminated poly(ethylene oxide) PEO as a macroinitiator and DEAEMA and methoxyethyl methacrylate (MEMA) as ATRP monomers [164]. PEO was chosen as a hydrophilic block due to its biocompatibility and prolonged in vivo circulation time, MEMA to provide hydrophobicity to the poly(2-(N, N’-diethylamino)ethyl methacrylate)-*stat*-methoxyethyl methacrylate) (P(DEAEMA-*stat*-MEMA)) block (*stat* stands for the statistical distribution of monomers within this block) and ultrasound responsiveness, while DEAEMA was chosen for its pH responsiveness. Polymersomes, assembled from those polymers, decrease by 40% in size when sonicated with 40 W power and disassemble at a pH of 5.83. Polymersomes loaded with DOX hydrochloride (DOX·HCl), a chemotherapeutic agent, were tested both in vitro and in vivo. The results demonstrated that ultrasound, a non-invasive stimulus, is a valid drug-release switch, that polymersomes can successfully escape endo-/lysosomes, and that this polymersome drug system can significantly inhibit tumor growth (95% reduction in tumor mass in mice).

Other promising ATRP-synthesized polymersomes, which could serve as stimuli-responsive drug-releasing systems, are being researched. ARGET ATRP was utilized to synthesize giant, hybrid lipid vesicles from a MPEG-based macroinitiator as well as MMA and DMAEMA (monomers) [165]. 1-palmitoyl-2-oleoyl-*sn*-glycero-3-phosphocholine was used as a lipid fraction. Alan B. Gamble et al. developed polymersome systems by the ATRP reaction of 4-azidobenzyloxycarbonylaminoethyl methacrylate (ABOC) or 4-fluorobenzyloxycarbonylaminoethyl methacrylate (FBOC) (monomers), utilizing an MPEG-based macroinitiator [166]. This system proved to be pH-sensitive: it did not show particle distribution at neutral conditions, while at a pH of 4.5, the vesicles were distributed. However, to the best of the authors’ knowledge, both of these systems have not yet been tested as DDSs. Table 1 shows a literature review of polymersome-forming polymers, which were synthesized utilizing ATRP methods, which includes both the already tested DDSs and those which are not fully developed yet.

### 3.3. Polyplexes

Polyplexes are artificial vesicles composed of interpolyelectrolyte complexes, which are typically made from two oppositely charged polyelectrolytes: positively charged polymers and negatively charged nucleic acids [167,168]. These opposite charges are responsible for the self-assembly of polyplexes through electrostatic condensation [169]. They can encapsulate drug molecules without chemically binding them, thus delivering them to target sites without any chemical modification and with unaffected intermolecular drug activity [170]. Moreover, positively charged polyplexes can destabilize the endosomal membrane of targeted cells as they cause the inflow of anionic molecules, creating osmotic pressure, which causes the disruption of the cellular membrane, thus leading to the internalization of the polyplexes [171,172].

Since polyplexes are capable of high-density payload condensation, they can penetrate the cell membrane, protect its contents (nucleic acids) from enzymatic degradation, and release it at the target site (for example, the tumor site). Therefore, they are widely researched as pDNA and mRNA delivery systems, especially as the injection of naked nucleic acids provides efficient protein expression in only very limited cases [173]. Moreover, polyplexes can be designed in such a way as to specifically recognize target cells. To achieve this, the polymers constituting polyplexes need to have well-defined properties, and controlled polymerization methods allow for such polymer synthesis [174,175]. ATRP is one of these methods, as it allows for great control of the polymerization or copolymerization of several monomers with low *Ð*_M_ and it allows for the incorporation of functionalized side-chains in the construction of block, alternate, and grafted copolymers. Polyplexes can be divided into vesicles serving solely nucleic acid-delivery functions and both drug- and nucleic acid-delivery functions.

#### 3.3.1. Nucleic Acid Delivery

T. Vermonden et al. recently designed a polyplex system utilizing two polymerization techniques. First, an NIPAM-based thermosensitive copolymer, PNIPAM-PEG-PEG-PNIPAM, was synthesized through traditional ATRP, using a hydrophilic PEG-based macroinitiator [176]. Then, it was polymerized with DMAEMA through free radical polymerization, leading to cationic block formation and a cloud point of 34 °C. The copolymers obtained formed polyplexes with pDNA under physiologically relevant conditions. The group compared this polyplex system with non-thermoresponsive polyplexes, assembled from PEG-based macroinitiators and DMAEMA only. They showed that the chain length of the copolymer determines the polyplex stability and that the NIPAM introduction to the polymer backbone through ATRP enables the formation of polyplexes with improved cytocompatibility, which could be caused by higher surface charge shielding. Transfection experiments revealed that thermosensitive polyplex systems could deliver nucleic acids to HeLa cancer cells, even in the presence of serum proteins. In a later study, the group showed that the thermosensitive polyplex systems obtained could be anchored in a thermosensitive hydrogel, which allowed for more controlled and sustained siRNA delivery when compared to free siRNA-hydrogel systems, leading to potential localized tumor treatment applications [177].

#### 3.3.2. Simultaneous Nucleic Acid and Drug Delivery

Polyplexes can be multifunctional—they can be designed to simultaneously deliver nucleic acids to a target site and additionally release drugs. Such a system was proposed by P. Ni et al. [178]. They reported a reduction- and pH-triggered dual-responsive triblock copolymer galactosamine-poly(ethylethylene phosphate)-*a*-poly(ε-caprolactone)-*ss*-poly(2-(dimethylamino)ethyl methacrylate) (Gal-PEEP-*a*-PCL-*ss*-PDMAEMA), prepared via the multi-step synthetic pathway, which included the ATRP of DMAEMA. These triblock copolymers could self-assemble into micelles and form polyplexes with green fluorescence protein-encoded DNA, were biodegradable, possessed low cytotoxicity, had a decent drug (DOX) loading capability, and could release the drug load in cancer cells in a fast manner. Moreover, they targeted HepG2 cells over HeLa cells as the former were overexpressing asialoglycoprotein receptors, which interacted with the galactosamine (Gal) ligand of the copolymers. This group’s research showed that their system is a promising dual-responsive DDS for simultaneous nucleic acid delivery and drug release.

Another simultaneous drug and nucleic acid delivery system was proposed by Y. Wu et al. [179]. Their biodegradable copolymer was based on a poly(3-hydroxybutyrate) (PHB)-based macroinitiator, with PHB obtained from renewable resources, and an ATRP-synthesized PDMAEMA block. The PHB block was introduced to counteract the cationic poly(dimethylaminoethyl methacrylate) (PDMAEMA) toxicity. The copolymer could form polyplexes with pRL-Relina plasmid DNA (pDNA). Tests showed that the proposed system had a better transfection efficiency than PEI (gold standard gene carrier) and that the nucleic acid and PTX drug co-delivery resulted in the death of increased drug-resistant cancer cells with a high expression of antiapoptosis Bcl-2 protein. Therefore, the PHB-PDMAEMA copolymers could be used for chemotherapy to effectively inhibit drug-resistant cancer cell growth. Table 2 shows a literature review of polyplex-forming polymers utilizing ATRP as their synthesis method.

## 4. Branched Copolymers in DDSs

### 4.1. Polymer Stars

The main advantage of using star copolymers in DDSs is their small hydrodynamic radius, which makes them easy to clear from in vivo systems. Compared with their linear analogs, amphiphilic star block copolymers can form aggregates in an aqueous solution with a high thermodynamic stability, leading to a relatively low critical aggregation concentration, which is very important for drug delivery carriers [180]. Additionally, if the controlled degradation of the star is applied, the system can be used to control the drug release rate. Star copolymers can be synthesized by core-first, arm-first, or coupling-onto methods [181]; however, the majority of the examples found in the literature in the last decade describe the use of the core-first strategy. This process relies on controlled polymerization in the presence of a well-defined initiator with a known number of initiating groups. The star copolymer is created in a one-step process; however, the number of arms is rather limited due to the small core molecules which are usually applied [182]. Star copolymers used for pH-responsive, thermo-sensitive drug delivery, and the delivery of nucleic acid-based drugs have been prepared by several research groups. Chmielarz et al. described the synthesis of six-armed copolymers with meso-inositol as the core and hydrophilic poly(di(ethylene glycol) methyl ether methacrylate) (PDEGMA) and amphiphilic poly(di(ethylene glycol) methyl ether methacrylate)-*b*-poly(methyl methacrylate) (PDEGMA-PMMA) as the arms [183]. These vitamin-based star polymers, produced by low-ppm ATRP, potentially can work as thermo-sensitive DDSs. In another study, a six-armed star triblock copolymer poly(2-(diethylamino)ethyl methacrylate)-*b*-poly(methyl methacrylate)-*b*-poly(poly(ethylene glycol) methyl ether methacrylate) (*s*-(PDEA_62_-*b*-PMMA_195_-*b*-PPEGMA_47_)_6_) was tested as a potential pH-responsive delivery carrier [184]. The results showed that the LC of the star copolymer was 33–35 wt% (relative to the polymer) at a pH of 7.4, 26–28 wt% at a pH of 10.5, and 10–15 wt% at a pH of 2.0. A bit lower LC was exhibited by the miktoarm star block copolymer, MPEG-*b*-P(MMA-*co*-MAA)_2_ [185]. The maximum values of the LC and drug encapsulation efficiency were 10.3% and 48.7% for MPEG-*b*-P(MMA_9_-*co*-MAA_35_)_2_ micelles and 16.5% and 82.3% for MPEG-*b*-P(MMA_24_-*co*-MAA_25_)_2_ micelles, respectively. Between 50% and 90% efficiencies of indomethacin encapsulation were also obtained for the four-arm star copolymers containing methyl (meth)acrylate and (meth)acrylic acid units [186]. The largest amount of drug (85%) was released within 96 h from micelles based on MA/MAA stars containing 24% of the hydrophobic fraction. An example of UV-cleavable unimolecular micelles was described by Liu [187]. Star-PMMA-PPEGMA, synthesized with photolabile *o*-nitrobenzyl groups at the cyclotriphosphazene core, turned out to have a great tendency to dissociate and release an encapsulated drug on dilution under physiological conditions.

In recent decades, cationic polymers have shown great competence in medical applications, including drug delivery. One of the most popular cationic polymers synthesized by ATRP is water-soluble pH-sensitive PDMAEMA. Due to the tertiary amine groups at the surface of the polymer, which become partially protonated at the physiological solution, DMAEMA possesses cationic charges [188]. An interesting example of an eight-armed star, positively charged copolymer was proposed by Zheng et al. [189]. In this case, star PDMAEMA was synthesized using a calix [4]-resorcinarene initiator and in the next step, hydrophobic blocks of poly(methyl methacrylate) or poly(butyl acrylate) were incorporated via the “one-pot” method. Star polymers with a narrow molecular weight distribution and particle size in the range of 20.3–36.6 nm were successfully obtained. A similar copolymer structure was proposed by Dworak [190]. In that case, star block copolymers were created from 28-arm poly(arylene oxindole) core, cationic DMAEMA, and nonionic (ethylene glycol) methyl ether methacrylate (DEGMA). The introduction of DEGMA segments into the star arms allowed for lower cytotoxicity in comparison to homopolymer PDMAEMA. These systems are dedicated to the delivery of plasmid DNA in gene therapy. In another study, Cho et al. designed PEG-based star polymers with a cationic core and evaluated their feasibility for nucleic acid delivery [191]. The star polymers were synthesized by the ATRP of DMAEMA and ethylene glycol dimethacrylate (EGDMA). The obtained polyplexes exhibited a high efficiency in nucleic acid delivery, particularly at relatively low star polymer weights or molar ratios.

Degradation is important in drug delivery to reduce the accumulation of polymeric materials in the body. Smaller fragments can be easily metabolized and subsequently excreted out of the body [192]. A combination of ATRP and ROP can bring interesting star copolymers with a well-defined molecular weight, architecture, functionality, and biodegradability [193]. PCL is used as a biodegradable block in the majority of cases; however, some examples of PLA and polyglycolide also can be found [194]. Biodegradable polyesters, synthesized by the ring-opening polymerization of cyclic esters in the presence of tris(hydroxymethyl)ethane [195,196], pentaerythritol (redox and lower–upper critical solution temperature (LCST-UCST) thermoresponsive transition) [197,198], 2-azidoethyl D-gluconamide [199], hexakis[p-(hydroxymethyl)phenoxy]cyclotriphosphazen [200], and β-cyclodextrin core, can act as hydrophobic macroinitiators in ATRP reactions [180]. For example, the PCL-based core was modified to yield halogen-terminated, three-arm or six-arm star-shaped PCL-*b*-PHEMA macroinitiators for ATRP, from which self-assembling noncytotoxic micelles were formed [196]. The LC and drug encapsulation efficiency were higher for the six-arm structure than for the three-arm structure and reached 9.16 and 69.8%, respectively. The highest drug release cumulant of 6sPCL-*b*-PHEMA micelles could reach a high level of 75%. Thermosensitive and highly drug-loaded micelles were also prepared using the three-arm PLA macroinitiator (3-arm PLA-*b*-PNIPAM) [195]. The obtained system offers a stable and effective platform for cancer chemotherapy with camptothecin (CPT). In some cases, not only the macroinitiator was degradable. Recently, Teng and co-workers prepared a biobased miktoarm star copolymer from soybean oil, isosorbide, and caprolactone [201]. In their studies, they used 1,4: 3,6-Dianhydro-D-glucitol 2-acrylate 5-acetate monomer as the substitute for styrene.

### 4.2. Polymer Combs and Brushes

Polymer brushes and combs are long-chain polymers (backbones) or surfaces, to which linear polymers (side-chains) are attached [202,203]. In brushes, the distance between grafting points is smaller than the side-chains’ end-to-end distance, while in combs this distance is larger [204]. Both of these polymer classes can be stimuli-responsive, form vesicles, and be tailored to target specific cells; hence, they can be effectively utilized as DDSs. They can be synthesized by “grafting-to” (a chemical reaction between reactive groups of side-chains and backbone) and “grafting-from” (monomer polymerization from backbone active sites) approaches. The technique, which is nowadays most commonly used to synthesize polymer brushes by the “grafting from” approach, is ATRP [205].

To encapsulate drugs, polymer brushes and combs can form micelles. F. Cellesi et al. synthesized a series of comb and brush block PCL and PEG copolymers, with PEGMA being utilized as an ATRP monomer [206]. The copolymers’ self-assembly and dexamethasone (DEX) drug encapsulation capabilities were based on the PCL/PEG ratio and molecular weight. The best copolymer for drug delivery application was a brush grafted from a four-arm star-shaped backbone. Another interesting system was proposed by H. Wei et al. [207]. They synthesized a reduction-sensitive amphiphilic cyclic brush PHEMA-*g*-PCL-disulfide link-poly(oligo(ethyleneglycol) methacrylate) with an ATRP monomer oligo(ethyleneglycol) methacrylate (OEGMA). The copolymer self-assembles into micelles with enhanced stability, which could be destabilized by the reducing environment, such as the one in tumor cells. It can also encapsulate DOX, an anti-tumor drug. Therefore, this system could be useful in chemotherapy.

Polymer brushes can also form stimuli-responsive polymersomes. As cancer cells have different redox potentials than normal cells and the extracellular matrix, redox-sensitive polymersomes could be suitable for cancer therapy [208]. Veena Koul et al. tested this idea by utilizing a PLA-based macroinitiator for PEGMA ATRP [209]. The polymers formed were biocompatible, biodegradable, hemocompatible, and conjugated with folic acid, hence they were also redox-sensitive, pH-sensitive, easily self-assembled into polymersomes, and possessed a disulfide bridge in their polymer backbone, which prevents rapid drug release in cancer cells. Moreover, hydrophilic, polymeric chains of PEGMA monomeric units prevent proteins from being adsorbed on the vesicle surface, thus preventing the immune system response. Polymersomes loaded with DOX have shown different drug-releasing behavior in different pH and GSH concentrations (the substance responsible for different redox potential of cancer cells). In vivo studies have shown that polymersomes loaded with DOX lead to a 96% decrease in tumor volume in mice; hence, they are greatly superior to the free drug (25% decrease in tumor volume), but also to the marketed drug DOXIL (PEG-modified liposomal DOX, 70% decrease in tumor volume). Moreover, they did not display significant toxicity to the organism [210]. Yue Zhang et al. also proposed a stimuli-responsive polymersome system composed of brush copolymers. They polymerized 2-((adamantan-1-yl)amino)-1-(4-((2-bromo-2-methylpropanoyl)oxy)phenyl)-2-oxoethyl methacrylate (ABMA) by ATRP, which, after further reactions, formed P(OEGMA-*co*-ABMA)-*g*-PDEGMA graft copolymer and P(OEGMA-*co*-ABMA)-*g*-PDEGMA/β-CD-SG [211]. Both copolymers could self-assemble into polymersomes and proved to be thermo-responsive, as the PDEGMA chains collapse at 37 °C. Hence, these copolymers could be utilized as thermos-responsive DDSs; however, they have not yet been tested as such.

Polymer brushes and combs have also been widely utilized to form polyplexes. R. P. Vieira et al. in 2023 presented a deactivation-enhanced atom transfer radical polymerization (DE-ATRP)-synthesized copolymer, which could form polyplexes and be used for targeted nucleic acid delivery [212]. The DE-ATRP method was used as it allows for greater kinetic control compared to traditional ATRP [213]. The monomers used were EGDMA, DMAEMA, and plant-based β-pinene. PDMAEMA was chosen for its high gene compatibility and buffering capacity, EGDMA for its cross-linking ability and vinyl groups, allowing for post-polymerization functionalization reactions, and β-pinene to provide solution stability to polyplexes, as well as for its antibiotic resistance modulation and anticoagulant, antitumor, antimicrobial, antioxidant, anti-inflammatory, and cytoprotective properties [214,215]. The copolymer formed in a one-pot DE-ATRP reaction was a nanometric, hyperbranched amphiphilic material, which formed polyplexes with gWiz-GFP plasmid DNA (pDNA), with encapsulation values up to 75.1%. The β-pinene monomeric unit proved to provide the material with an excellent solution stability and high positive charge, allowing for smooth cellular membrane penetration. Polyplexes showed different transfection efficiency with different cell lines; hence, after further research, they can be potentially used as organ-targeted cell vectors for gene therapy. Another nucleic acid delivery polyplex system synthesized with the aid of ATRP was proposed by S. Averick et al. in 2017 [216]. They prepared fentanyl-chain-ended polymers for targeted delivery to neurons, or more specifically, to Mu opioid receptor (MOR) expressing cells. They used glycidyl methacrylate (GMA) and oligo((ethylene oxide)methacrylate) (OEOMA) as ATRP monomers and fentanyl species (Fen-Acry-EtBPA) as an ATRP initiator to produce diblock copolymers, which had a high affinity to MOR. The OEOMA was chosen for its hydrophilic properties and biocompatibility, while the GMA was chosen to provide post-polymerization functionalization opportunities [217,218]. At this step, the fentanyl conjugate was obtained; therefore, it will be discussed from this point of view in Section 6.2. The chain-end fentanyl polymers formed were fully biocompatible, formed polyplexes with siRNA, and could be bound and internalized by SH-SY5Y cells, which express MOR endogenously. The siRNA binding properties were proven to be correlated to polymer length and charge, with longer polymer chains with a higher cationic charge binding siRNA more efficiently. This allows for tuning the copolymer and hence the polyplex properties; however, further studies are required to improve and optimize nucleic acid delivery. In 2017, an acid-sensitive polyplex gene vector system was proposed by X. Jiang et al., which was achieved through ATRP and ring-opening reactions [219]. GMA was used as an ATRP monomer and modified poly(β-cyclodextrin) as an initiator. The copolymer formed was later modified with ethanolamine to form brush-shaped, pH-sensitive, cationic host modules. The polyplexes were assembled with pcDNA3-Luc pDNA and then modified with adamantly based guest molecules. This not only provided the polyplexes with a stealth effect, which improved nanoparticle stability, but also allowed for targeted nucleic acid delivery, as the polyplexes targeted cells that were over-expressing folate receptors, just like cancer cells. This system also possessed a high gene condensation capability, low cytotoxicity, and high transfection efficiency. V. Koul et al. also proposed a polyplex DDS in 2017 [220]. They used ATRP to synthesize redox-sensitive polymer PPEGMA-*s*-*s*-PCL, with PEGMA as an ATRP monomer. The PEGMA side chains were short, which led to a negligible chance of evoking an immune system response and a stealth effect. The copolymers could self-assemble with pololike kinase 1 siRNA to form polyplexes, which could be loaded with DOX. The drug release could be regulated by low pH and redox conditions and the simultaneous nucleic acid and drug release has led to tumor growth inhibition during tests, making these polyplex systems suitable for tumor-specific delivery.

Table 3 shows a literature review of polymer combs and brushes synthesized utilizing ATRP, which includes both the already tested drug-releasing systems and those which are promising but not fully developed yet.

## 5. Smart DDSs Based on Nanoparticles Coated with Polymers Obtained via ATRP

Recently, several research groups proposed a novel strategy for utilizing ATRP in smart DDS synthesis. It envisages drug delivery via hybrid nanoparticles (HNPs) exhibiting a core-shell structure—these hybrid nanocarriers consist of an inorganic core made of metal or metal oxide nanoparticles, whose surface is modified with polymeric chains made by ATRP. A substantial advantage of this type of DDS is its obvious resistance to the destruction and premature release of a therapeutic payload (due to the dilution in the bloodstream) in comparison to micelle-based analogs, which are always characterized by the specific values of CMC.

### 5.1. Metal Oxide-Based Nanocarriers

Ensafi and coworkers utilized 2-bromopropionyl bromide for the modification of the hydroxyl-containing surface of the hydrothermally synthesized nanoparticles of ZnO or TiO_2_, thus obtaining macroinitiator nanoparticles from which the ATRP of DEAEMA was started [221]. Blank HNPs grafted with PDEAEMA chains had hydrodynamic diameters of ca. 55 nm, which were increased to 75–85 nm after loading with the anticancer drug flutamide. The drug was loaded into the PDEAEMA shell in situ (during ATRP in the presence of the dissolved flutamide) and kept there via hydrogen bonding between its amine groups and carbonyl functionalities of the grafted polymer. HNPs with flutamide percentages 2–10% (in relation to the content of PDEAEMA) were tested showing an accelerated drug release at acidic conditions (pH = 5) [221].

**Figure 11:**
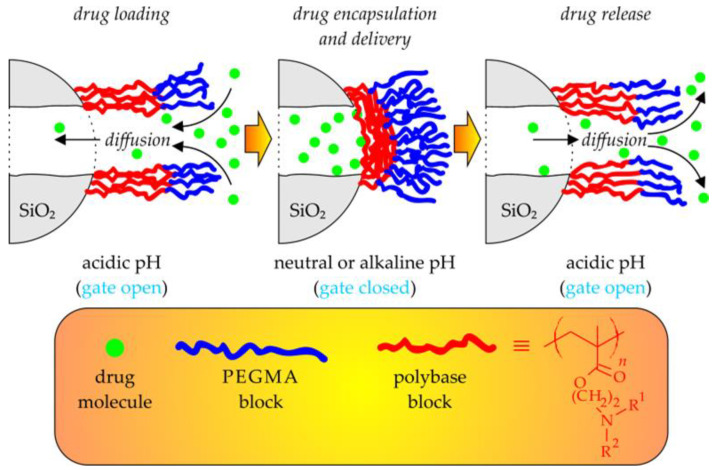
Schematic illustration of drug loading, delivery, and release from the surface-modified HNPs containing mesoporous silica cores.

A different type of HNP was investigated by Alswieleh and coworkers, who modified mesoporous silica nanoparticles with amphiphilic (co)polymers containing PDEAEMA and poly(oligo(ethylene glycol) methyl ether methacrylate) (POEGMA) or poly(2-(*tert*-butylamino)ethyl methacrylate) (PTBAEMA) and PEGMA as hydrophobic and hydrophilic blocks, respectively [222,223]. In both cases, before the ATRP procedure, the surface hydroxyl groups of the SiO_2_ nanoparticles were subjected to silanization with (3-aminopropyl)triethoxysilane (APTES) and then amidation with BIBB. In the case of the PDEAEMA-based system, hydrophobic and hydrophilic blocks were linked together via succinic acid and cysteine linkages [222], whereas the PTBAEMA block was directly copolymerized (by ATRP) with hydrophilic PEGMA [223]. Therefore, the synthesized HNPs were utilized as nanocarriers for water-soluble drugs, such as anticancer DOX (in its hydrochloride form, DOX·HCl) [222] or doxycycline (a tetracycline antibiotic) [223], exhibiting drug loading efficiencies of 69% or 38–44%, respectively. Interestingly, the mechanism of drug loading in these polymer-modified HNPs is based on the physical entrapment of drug molecules inside pores present in the silica core, although it can be additionally supported by interactions with drug-complexing functional moieties (e.g., amine groups) introduced to the surface of silica pores [223]. Moreover, in this type of DDSs, the ATRP-derived chains of hydrophobic polymers, containing tertiary amine units, function as the pH-responsive gatekeepers. Due to electrostatic repulsion forces, caused by protonation at acidic pH, hydrophobic polymers stretch out from the surface of HNPs opening pores for the diffusion of drug molecules, then at physiological pH, they collapse onto the surface of HNPs, closing the pores and entrapping the drug within them, and finally, after endocytosis into tumor cells, the polymers once again open the pores in the silica core, releasing the drug directly into endo-/lysosomes (see Figure 11).

It is worth noting that although the properties of the above-mentioned systems (e.g., their pH-responsive behavior) depend on the type of monomer and processing condition chosen for ATRP, they can be also tuned during post-polymerization chemical modification. For that purpose, one can utilize a well-known quaternization reaction with alkyl iodide to introduce new organic substituents and/or cationic sites to the amine-containing pendant groups of polymer chains grafted from a silica surface via ATRP. An example of such modification was reported by Alswieleh and coworkers in the case of the PTBAEMA, which was reacted with 2-bioethanol [224]. Depending on the reaction time and the number of quaternized amine units, HNPs behave differently at acidic conditions.

Li and coworkers proposed a different version of mesoporous silica HNPs for the delivery of DOX·HCl [225]. Instead of one amphiphilic LBCP, they used two separate homopolymers (hydrophilic MPEG and hydrophobic poly(2-(1-piperidino)ethyl methacrylate) synthesized via ATRP) for the modification of the surface of silica nanoparticles. Nevertheless, the mechanism of drug loading/release (the “gatekeeper model”) did not change. The authors obtained DOX·HCl-loaded HNPs with hydrodynamic diameters below 100 nm and a high drug encapsulation efficiency (ca. 65%). At physiological pH, these DDSs were stable, showed no signs of an unfavorable aggregation, and only minimal drug release (15% after 40 h of incubation), whereas at acidic conditions, they liberated DOX·HCl in a much-accelerated manner (the cumulative release after 40 h was 68% and 84% at pHs of 6.5 and 5.0, respectively). They were also characterized by very good cytotoxicity against HeLa tumor cells.

Zhang and coworkers extended the “gatekeeper” strategy on HNPs containing pH-cleavable linkers between their inorganic, mesoporous cores and polymeric coronas [226]. They loaded DOX (loading content of ca. 14%) into the pores of APTES-modified mesoporous SiO_2_ nanoparticles and subsequently closed the pores by covalently grafting their surface with the chains of an amphiphilic copolymer containing a poly[p-(2-methacryloxyethoxy)benzaldehyde] (PMAEBA) hydrophobic block and PPEGMA hydrophilic block. pH labile imine linkages were formed in the reaction of the copolymer’s pendant aldehyde groups with amine groups located on the surface of silica. ARGET ATRP was used to copolymerize PEGMA and the benzaldehyde-bearing monomer, in the presence of ethyl 2-bromoisobutyrate initiator and tin(II) 2-ethylhexanoate. At physiological pH, the DOX-loaded HNPs showed a very limited drug release (the cumulative DOX release was less than 20% after 3 days of incubation), whereas at a pH of 5.0, this process accelerated more than three times due to the imine bond-breaking, detachment of the copolymer “gatekeeper”, and opening of the DOX-loaded pores. Tests showed that this DDS exhibited a good cellular up-take by the HepG2 liver tumor cells, as well as increased cytotoxicity toward them (the viability of HepG2 cells cultured at the 20 mg/L concentration of the DDS decreased to less than 20% after 48 h of incubation) [226].

It should be noted that a physical entrapment of the drug within the pores of mesoporous silica is not the only pathway leading to drug-loaded silica HNPs. Wei and coworkers showed that the latter can be obtained by a simple complexation of amine-bearing drug molecules (e.g., cisplatin) with polyacid copolymers attached to the surface of mesoporous silica nanoparticles [227]. They covalently attached BIBB on the surface of the APTES-modified silica and then utilized a surface-initiated metal-free ATRP procedure to copolymerize itaconic acid (a dicarboxylic unsaturated acid) with PEGMA. Interestingly, this polymerization was induced by an organic catalyst (10-phenylphenothiazine) and visible light instead of the conventional metal/amine ligand catalyst system. The pendant carboxylic groups in the copolymer shell of the obtained HNPs strongly complexed cisplatin at physiological pH and easily released it upon acidification: a cumulative cisplatin release after 48 h increased from less than 10% at a pH of 7.4 to ca. 60% at a pH of 5.5.

Alswieleh and coworkers also studied magnetic mesoporous HNPs, although they utilized them as solely pH-responsive DOX-delivering nanocarriers [228]. Their preparation included the coating of Fe_3_O_4_ nanoparticles with mesoporous silica, surface functionalization with an ATRP initiator, the grafting of PDEAEMA chains via the ARGET ATRP technique, and the optional capping of the PDEAEMA chain-ends with folic acid. The authors showed that due to the protonation of tertiary amine in the PDEAEMA units, the nanoparticle’s dimensions increased to ca. 750 nm in acidic media (from the initial size of ca 450 nm in a neutral or slightly alkaline environment) and the entrapped DOX was released at an accelerated rate (16% at pH = 5.0 vs. <6% at pH > 7) [228]. The double responsivity of the Fe-containing HNPs to the pH and magnetic field was experimentally proven by He and coworkers, who investigated water-soluble Fe_2_O_3_ nanoparticles with a dendritic–linear-brush-like triblock copolymer located on their surface [229]. ATRP was utilized for a sequential synthesis of the linear part of the copolymer—first the hydrophobic block of PDMAEMA, and then the hydrophilic block of PPEGMA. Field-dependent magnetization tests showed that at room temperature, the obtained HNPs exhibited superparamagnetic properties (e.g., no hysteresis on the magnetization–magnetic field curves) and their saturation magnetizations were within the limits usually accepted for magnetic particles destined for biomedical applications. As for the drug release properties, these Fe_2_O_3_-based HNPs could be loaded with DOX up to a loading capacity of ca. 7% and showed a prolonged profile of DOX release in HeLa-line tumor cells, while maintaining good biocompatibility and a very low cytotoxicity against healthy cells.

### 5.2. Metal-Based Nanocarriers

**Figure 12:**
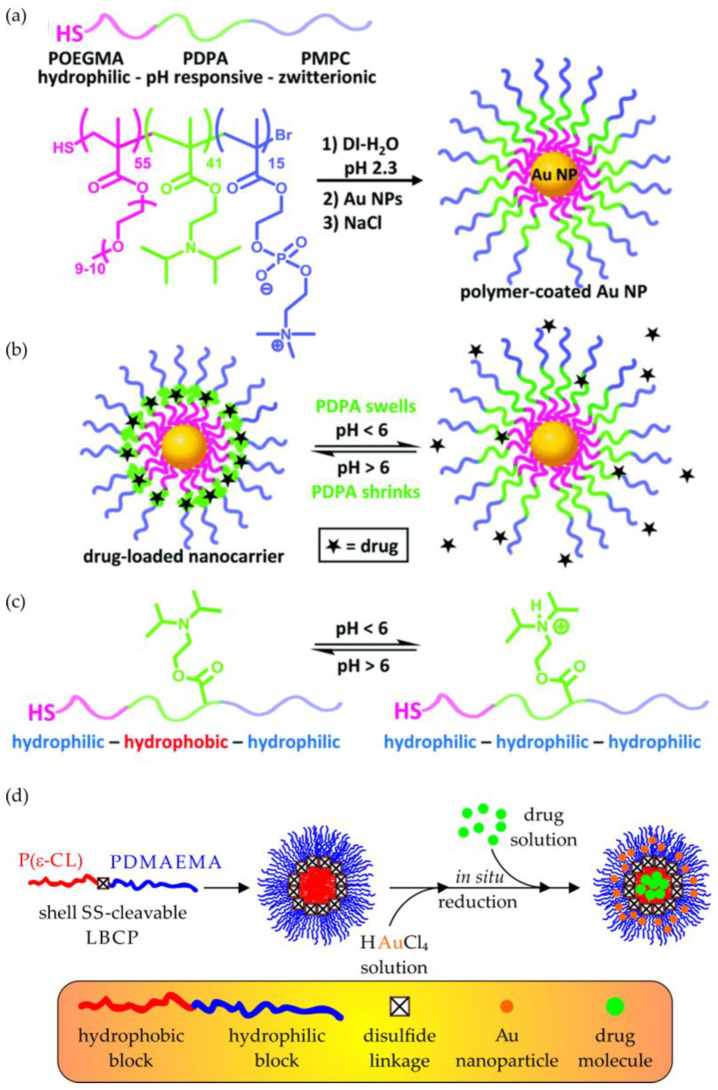
Representative synthetic pathways and main types of metal-containing drug delivery nanocarriers: (**a**–**c**) polymer-coated HNPs (reproduced from Ellis, E.; Zhang, K.; Lin, Q.; Ye, E.; Poma, A.; Battaglia, G.; Loh, X. J.; Lee, T.-C., Biocompatible pH-responsive nanoparticles with a core-anchored multilayer shell of triblock copolymers for enhanced cancer therapy. Journal of Materials Chemistry B 2017, 5 (23), 4421–4425, DOI: 10.1039/c7tb00654c, https://pubs.rsc.org/en/content/articlehtml/2017/tb/c7tb00654c (accessed on 26 February 2023) with permission from the Royal Society of Chemistry); (**d**) micelles with metal nanoparticle-containing coronas.

Over the last decade of scientific research, two main types of metal-containing polymeric DDSs have been investigated. Depending on the location of the zero-valent metal component within these nanoassemblies, one can distinguish HNPs with a central metal core or micelles having metal aggregates in their hydrophilic coronas. Their structures and synthetic strategies are shown in Figure 12.

The utilization of metal nanoparticles as the inorganic cores of HNPs used for drug delivery was demonstrated by Lee and coworkers in their example of polymer-coated gold nanoparticles (hydrodynamic diameters of ca. 60 nm) subjected to loading with DOX (47% of LC, encapsulation efficiency ca. 37%) [230]. The organic coating was made of a double hydrophilic triblock copolymer, namely poly(oligo(ethylene glycol) methyl ether methacrylate)-*b*-poly(2-(*N*,*N*’-diisopropylamino)ethyl methacrylate)-*b*-poly(2-(methacryloyloxy)ethyl phosphorylcholine) (POEGMA-*b*-PDPAEMA-*b*-PMPC), anchored to the surface of the gold nanoparticles through the sulfide bond (the “grafting onto” approach). POEGMA-*b*-PDPAEMA-*b*-PMPC chains were synthesized via a reductive cleavage of the RS–SR moiety present in a symmetrical, hexablock copolymer produced through a sequential ATRP, which was started on a bifunctional initiator containing a disulfide bridge. The synthesized Au HNPs exhibited much better protein antifouling properties than their analogs coated with PEG chains, whereas their cytotoxicity against the MCF-7 breast cancer cells was greatly enhanced compared to the free DOX at the same concentration (the normalized cell viability was ca. 25% vs. ca. 80%, respectively). The pH-triggered DOX release from such HNPs (in an acidic environment) was explained by the protonation of the pendant tertiary amine groups, present within the central block of the POEGMA-*b*-PDPAEMA-*b*-PMPC copolymer, leading to its hydrophilization and electrostatic swelling [230].

Micelles with zero-valent gold nanoaggregates in their hydrophilic coronas have been reported by Zhang and coworkers [231,232]. The authors proposed that the starting point for the synthesis of the drug nanocarrier should be the preparation of either dynamic or static copolymeric micelles containing PDMAEMA blocks [231,232]. The former were obtained via the conventional self-assembling of amphiphilic LBCP chains containing redox-responsive disulfide linkages at the PCL/PDMAEMA block junction. This shell-sheddable copolymer was synthesized from a bifunctional initiator, 2-hydroxyethyl-2′-(bromoisobutyryl) ethyl disulfide via the ROP of ε-CL followed by the ARGET ATRP of DMAEMA [231]. On the other hand, single-molecule micelles resistant to dilution (unimolecular micelles) were obtained from amphiphilic 21-arm star-like copolymers composed of poly(lactide)-*b*-poly(2-(*N*,*N*-dimethylamino)ethyl methacrylate)-*b*-poly[oligo(2-ethyl-2-oxazoline) methacrylate] chains connected to β-cyclodextrin. First, β-cyclodextrin was used as a macroinitiator in the ROP of lactide and then, after esterification with BIBB of the hydroxyl chain-ends in the newly formed PLA, as a macroinitiator during a sequential ARGET ATRP of DMAEMA and oligo(2-ethyl-2-oxazoline) methacrylate [232]. The presence of the pendant tertiary amine moieties in both types of micelles was crucial for the next step of the synthesis (i.e., the formation of gold nanostructures) since they were able to actively reduce [AuCl_4_]^−^ ions to Au^0^. Thus, after the infusion of the PDMAEMA-containing micelles with an aqueous solution of HAuCl_4_, an in situ [AuCl_4_]^−^→Au^0^ reduction proceeded within the internal PDMAEMA layer of the micelle’s hydrophilic corona. In both systems, the obtained gold nanoparticles had a uniform distribution and diameters of less than 10 nm [231,232]; however, their sizes strictly depended on the HAuCl_4_ and copolymer concentration (a higher concentration of reagents promoted the generation of larger Au structures), PDMAEMA block length (the longer it was, the smaller the gold nanoparticles were), and the tertiary amine/HAuCl_4_ molar ratio (a higher molar excess of the reducing groups resulted in smaller gold nanoparticles) [232]. Zhang’s group reported that, regardless of their type, the Au-bearing micelles showed no significant cytotoxicity since the cell viability was over 80% even at the highest concentration of the micelles [231,232]. Moreover, the dynamic micelles made of the sulfide-containing LBCP could be loaded with DOX and exhibited an accelerated DOX release at the lysosomal pH and reductive microenvironment [231].

## 6. Bioconjugates

A large number of the drugs currently used are small-molecule compounds, which means that therapy using this form of the drug may have disadvantages, such as limited solubility, drug aggregation, low bioavailability, poor biodistribution, lack of selectivity, difficulties with targeting the therapeutic effect, and the troublesome side effects of therapeutic drugs [233]. These difficulties are being solved by the development of new preparations with better effects, acting in accordance with the principles of the DDS. An ideal DDS should allow the conjugate to find the target cell and freely penetrate the cell membrane, resulting in entry into the cell nucleus. In addition, the active substance should not be released until it has found its target cell. Through appropriate conjugation, the therapeutic efficacy of a drug can be improved, and toxic effects can be significantly reduced by increasing the amount and persistence of drugs in the vicinity of target cells while reducing drug exposure to non-target cells [234]. Drugs can be taken in different ways, such as by mouth, by inhalation, by skin absorption, or intravenously. Each method of drug delivery to the body has pros and cons. In addition, the method of delivery is strictly related to the type of therapeutic agent. Administering drugs topically, rather than systemically (affecting the whole body), is a common way to reduce the side effects and toxicity of drugs while maximizing the impact of treatment [235,236].

One of the most common methods of drug delivery is the conjugation of an active substance on a polymer carrier. This technique offers several benefits, including improved drug solubility, prolonged circulation, reduced immunogenicity, controlled release, and increased safety. In addition, it is possible to create an advanced complex DDS that, in addition to the polymer and the active substance, may contain other active ingredients that enhance the activity of the main drug [237,238,239]. Furthermore, polymeric materials are widely used in biomedical applications, such as implants, surgical sutures, tissue engineering, and many others [240,241,242]. The development of polymers as carriers of bioactive pharmaceuticals started relatively recently. In the past, it was believed that polymers were too heterogeneous in terms of molar mass, composition, and structure to be useful in the production of therapeutics. This approach changed in 1975 with the development of the first polymer anti-cancer drug by Ringsdorf, which ushered in a new era of polymer conjugate research. Ringsdorf proposed a macromolecular conjugate model that consists of a polymer backbone with three distinct regions. The first region contains moieties that modify the solubility of the conjugate, the second contains the drug (attached via a biodegradable linker), and the third contains tropic molecules (responsible for target cell recognition) [243,244,245]. One of the flagship, highly versatile, efficient, and sustainable controlled radical polymerization techniques is ATRP, which allows for obtaining functional polymers with well-defined structural parameters, such as molar mass and its distribution, as well as a specific architecture [246]. It is widely used as a technique for designing and obtaining multifunctional, nanostructured materials for various applications in the pharmaceutical, medical, and biotechnology industries, including drug delivery systems [35]. In the further course of this work, examples of polymer conjugates with active substances, such as proteins or therapeutic drugs, obtained using the ATRP technique will be presented.

### 6.1. Protein–Polymer Conjugates

Due to several advantages mentioned in the previous subchapters, ATRP techniques have found wide application in the pharmaceutical, medical, and biotechnological industries. In this section, examples from the literature of the synthesis of polymer–protein conjugates using the ATRP technique will be presented. Systems with this structure are produced to improve the efficiency of drug delivery and operation, as well as to improve their pharmacological properties. Proteins have found use as therapeutics due to some of their specific features, such as a relatively large size, high degree of structural definition, biocompatibility, and a range of diverse biological functions. However, some specific characteristics of proteins (short half-life, poor stability, low solubility, and immunogenicity) limit their wide application, making their attachment to a polymer matrix essential in the preparation of effective therapeutic drugs [247,248,249].

**Figure 13:**
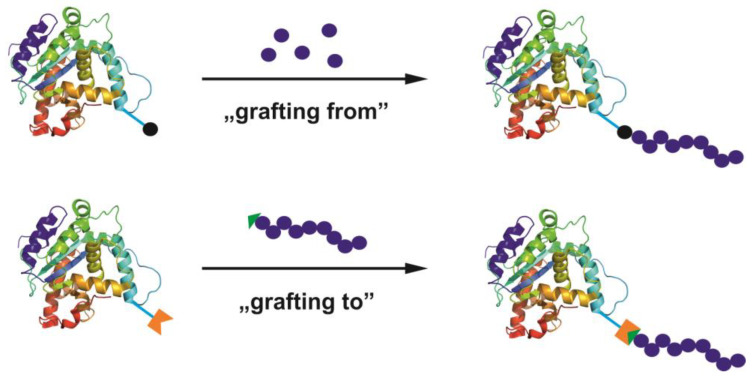
Two main strategies for obtaining polymer–protein conjugates.

In laboratory practice, two main strategies for obtaining polymer–protein conjugates can be distinguished [250], which are presented in Figure 13. The first widely used strategy is the “grafting to” method. It consists of the initial synthesis of the polymer, which in the next stage is directly attached to the protein structure. This strategy clearly has its advantages because the polymer can be synthesized under any conditions before the final step of protein conjugation. However, disadvantages such as the low efficiency of the reaction between two large molecules and difficulties in purifying the products limit its further use [248,249,251]. The second widely used strategy is the “grafting from” method. It consists in transforming the structure of the protein, creating a macroinitiator capable of initiating the processes of controlled living polymerization, including the ATRP technique widely used in this strategy. In the next stage, the actual process of obtaining the conjugate takes place through the process of the polymerization of individual classes of monomers. The main advantage of this method is the ease of separation of small monomer molecules from protein–polymer conjugates after polymerization [252,253].

Bontempo and co-workers have proposed a method for the synthesis of polymer–protein conjugates using the “grafting to” strategy, utilizing the ATRP technique in one of the stages and more specifically its classic variant. The first step is the ATRP of the HEMA monomer on a pyridyl initiator, containing disulfide groups, at room temperature. The PHEMA polymer is widely used in biomedical applications due to its easy and controlled polymerization process and biocompatibility, having a hydrophilic group, and forming a gel form when in contact with water, i.e., in the human body. The polymer synthesized in this way was reacted with protein and more specifically with bovine serum albumin (BSA), which is a standard reference protein used for various studies due to its availability and relatively low price. In its structure, it has cysteine, capable of forming sulfide bridges. This ability was used in the final conjugation process by introducing the previously obtained PHEMA polymer into the structure of the BSA. According to the authors, this strategy can be applied to the production of a wide range of polymer–protein conjugates without the need for post-polymerization modification of the polymers [254]. Another example of the application of the ATRP technique in the synthesis of protein–polymer conjugates by the “grafting to” strategy is the work of Sayers et al. In the first step, by polymerizing the PEGMA monomer on an aldehyde initiator with ATRP, several well-defined PPEGMA polymers were obtained. PPEGMAs were then conjugated to salmon calcitonin, a calcitropic hormone currently administered to treat a range of hypercalcemia-related diseases, by forming an N-terminal Schiff base followed by a reduction with sodium cyanoborohydride [255]. In vitro biological tests have shown that polymer conjugation does not affect the biological activity of the protein. According to the authors, the approach developed in this study appears to be of general application and could potentially open the door to the use of α-aldehyde coupling materials with different architectures for the N-terminal conjugation of a wider range of biologically relevant therapeutic proteins [256].

Notwithstanding, due to its undeniable advantages, in recent years, the strategy path has been mainly developed using the “grafting from” method, in which the processes of controlled radical polymerization play a key role, of which the lion’s share are syntheses using the ATRP and RAFT techniques [67,257,258]. An interesting way of synthesizing polymer–protein conjugates using the “grafting from” strategy, as well as using ATRP techniques, has been proposed by Cummings et al. [259]. They presented a three-step synthesis of a block copolymer using an initiator with a protein in its structure. The present paper describes a novel method of protein permeation enhancement through a polymeric additive. The first step was the synthesis of an initiator based on the BSA protein, which would gain a halogen atom capable of initiating the ATRP process of the PEGMA monomer in the next act. Then, in the third step, also via ATRP, a block of N-(3-(4-phenylpiperanysyl)propyl)acrylamide was added, which, according to previous reports, may be useful as an intestinal permeation enhancer. The authors proved, that, by incorporating a block of permeation-enhancing polymer, absorption through the intestinal monolayers was increased up to 35 times compared to that of unmodified protein. The team led by Professor Dworak also researched the synthesis of polymer–protein conjugates [260]. They proposed the synthesis of an enzymatically cleavable hybrid biomaterial—poly(N-isopropylacrylamide)-pentapeptide (Gly-Arg-Lys-Phe-Gly-dansyl) conjugate, using the ATRP technique. The researchers showed that due to the hydrophilic nature of the pentapeptide bound to the polymer chain, the bioconjugate exhibited a higher phase transition temperature than that of the corresponding homopolymer. Moreover, the bioconjugate chains were able to form small-sized mesoglobules by rapidly heating the bioconjugate solution. It was also shown that the peptides formed the outer layer of the mesoglobula, which made them fully accessible to the enzyme, and the introduction of arginine or lysine into the bioconjugate structure provided the possibility of cleaving the peptide segment from the polymer anchor, which could be useful for peptide release.

Protein–polymer conjugates using the “grafting from” strategy can also be prepared by various variants of the ATRP technique. Cohen-Karni et al. proposed the synthesis of conjugates based on the well-defined acrylamide, N, N-dimethylacrylamide, and N-vinyl imidazole homo and block copolymers from a model protein BSA initiator under bio-relevant conditions, using the ICAR ATRP technique [261]. This technique allows for a significant reduction in the amount of copper catalyst needed, even to a level below 100 ppm [262]. In addition, using N-vinyl imidazole as a catalytic ligand, the authors prepared, by loading palladium, a biohybrid catalyst that successfully catalyzes the Suzuki-Miyaura coupling in an aqueous environment under aerobic conditions [261]. Moncalvo et al. synthesized and characterized various lysozyme-PPEGMA and lysozyme-poly(glycerol monomethacrylate) (PGMMA) conjugates in terms of topology (linear or bi-armed) and molar mass [263]. The process was carried out using the ARGET ATRP variant. These results highlighted the potential of PGMMA as an alternative to polyethylene glycol in extending the half-life of biotherapeutics. PGMMA is a hydrophilic synthetic polymer with a low toxicity and very limited interactions with proteins. In addition, the two hydroxyl groups it has in its structure can be easily functionalized to obtain various variants of conjugates. Researchers have also shown that appropriate polymer architecture design can help reduce enzymatic degradation.

Table 4 below presents a review of the literature on the synthesis of modern polymer-protein conjugates using the “grafting from” strategy and various ATRP techniques, depending on the type of conjugated protein and monomers used. It shows the enormity of work recently put in by scientists in the development of this type of conjugates, as well as the commonness and versatility of various variants of the ATRP technique.

### 6.2. Drug–Polymer Conjugates

The development of polymer carrier-based drug delivery vehicles is a very fast-growing field that has many advantages, such as the selective targeting and prolonged circulation of the therapeutic. The delivery of new therapeutic agents, combination therapies, and novel polymer architectures are very exciting and promising areas. Undoubtedly, the ATRP technique helps to create more and more new solutions in this field. In this section, examples from the literature of the synthesis of polymer–drug conjugates using ATRP techniques will be presented [5,35,237].

**Figure 14:**
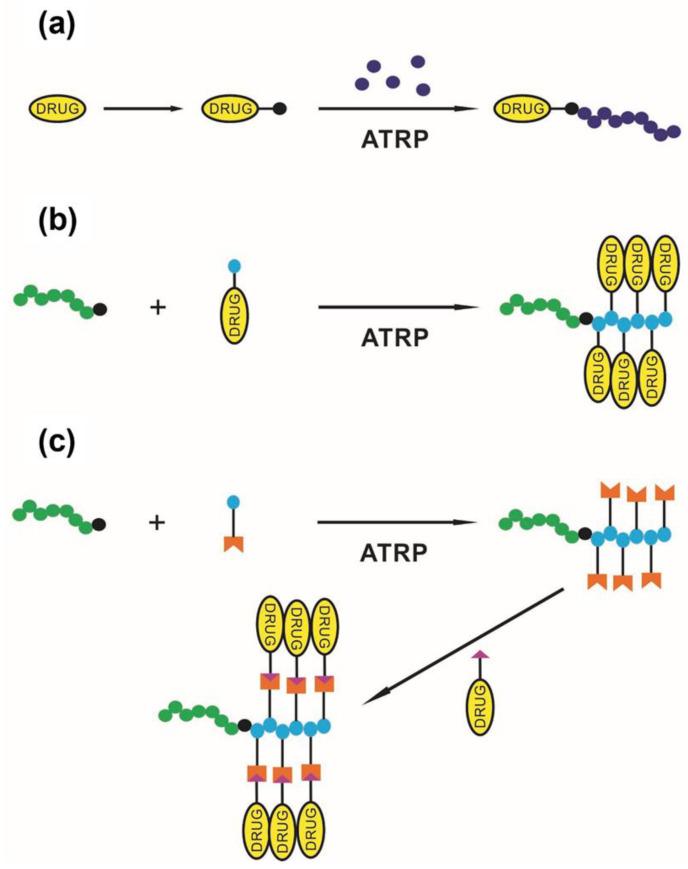
Three main strategies for obtaining covalently linked polymer–drug conjugates: (**a**) active substance as an ATRP initiator, (**b**) active substance as (meth)acrylate monomer, and (**c**) post-polymerization conjugation.

In laboratory practice, there are three types of strategies for obtaining covalently bound polymer–drug conjugates using ATRP techniques, which are presented in Figure 14. The key aspect is the creation of unstable covalent bonds between the drug and the macromolecular backbone. Various ways can be used to chemically link bioactive molecules to polymer chains through hydrolyzable or biodegradable bonds (for example, ester or carbonate bonds) [275].

The first strategy is the possibility of introducing a terminal halogen group into the chemical structure of the drug, capable of initiating the ATRP reaction, similar to the “grafting from” strategy shown in the example of polymer–protein conjugates. Cohen-Karni et al. developed the synthesis of a polymer–drug conjugate based on a derivative of fentanyl acting as an AGET ATRP initiator, classified as a narcotic analgesic [216]. The paper is discussed herein in Section 4.2 due to the ability of the diblock co-polymer to form polyplexes with siRNA; however, formally, the copolymer initiated with the fentanyl derivative is a polymer–drug covalent conjugate. The mechanism of action of the drug consists in the fact that the active substance contained in the drug binds to opioid receptors in the body. As a result of this connection, these receptors are stimulated [276,277]. The main task of fentanyl is to efficiently target the MOR receptor for neuronal targeting. The introduced polymer matrix retains a high degree of binding to the receptors and allows the modification of its structure by reacting the functional groups of the monomer used, GMA in this case, in order to attach a near-infrared fluorescent dye (ADS790WS) or to build a targeted siRNA delivery system by modifying groups with secondary amines. The results obtained support the possible use of this system for delivery to MOR-expressing cells. Li et al. have developed dual-sensitive and time-controlled cationic liposomes based on a conjugate of CPT with polymeric carriers for the co-delivery of siRNA for anticancer therapy [278]. CPT is a monoterpene-indole alkaloid of natural origin, with a strong anticancer effect. This substance inhibits the activity of Topoisomerase I, an enzyme that is involved in the process of DNA replication and transcription, causing damage to the genetic material, which leads to cell death [279,280]. The pH-sensitive zwitterionic poly(carboxybetaine) polymer was conjugated to CPT via the ATRP process, using a CPT derivative having a halogen atom in its structure as the initiator. CPT-based cationic liposomes, consisting of the prepared conjugate and a cationic lipid, were then constructed for the co-delivery of siRNA for combination therapy. The double-sensitive lipoplexes simultaneously delivered two drugs to the tumor cells and enabled time-controlled drug release, such that siRNA was released rapidly after a 4 h incubation and CPT was released in a sustained manner.

**Figure 15:**
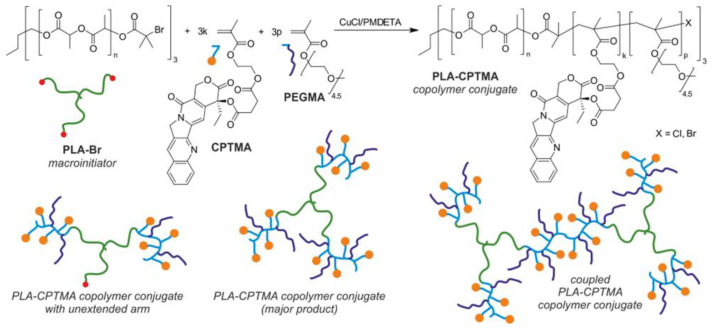
Synthesis of conjugates of PLA and CPT block copolymers proposed by Plichta et al. [281]. Reprinted with permission from Plichta, A.; Kowalczyk, S.; Kamiński, K.; Wasyłeczko, M.; Więckowski, S.; Olędzka, E.; Nałęcz-Jawecki, G.; Zgadzaj, A.; Sobczak, M., ATRP of Methacrylic Derivative of Camptothecin Initiated with PLA toward Three-Arm Star Block Copolymer Conjugates with Favorable Drug Release. Macromolecules 2017, 50 (17), 6439–6450, DOI: 10.1021/acs.macromol.7b01350 (accessed on 26 February 2023). Copyright 2017 American Chemical Society.

The second strategy is the possibility of refunctionalization of the therapeutic by introducing into its structure (meth)acrylic moieties which are able to undergo ATRP processes to form polymer–drug conjugates. Plichta et al. proposed a method for the synthesis of polymer–drug conjugates using ATRP macroinitiators based on PLA and the produced methacrylic derivative of CPT, which was conjugated on a polymer matrix [281]. In addition, in some syntheses, an additional PEGMA block was added. This process and the structures of the obtained conjugates are shown in Figure 15. The great strength of this type of conjugation is the possibility of introducing more than one molecule of the active substance per chain of the polymer matrix, in contrast to the first presented strategy, in which only one molecule could be introduced per entire polymer chain. The CPT content of the conjugates was determined using three techniques and ranged from 8 to 16.9 wt%. The release profile of CPT was also examined, with which it was shown that the more D-LA units in the structure, the slower the release of the active substance, while the PEGMA groups acted antagonistically towards D-LA. Gao et al. developed an amphiphilic copolymer based on a hydrophilic beta-cyclodextrin derivative used as the initiator of the ATRP process to embed a methacrylic derivative of the hydrophilic anticancer drug irinotecan onto a polymer matrix [282]. The obtained star-shaped amphiphilic copolymer had the ability to form stable monomolecular micelles in an aqueous solution, the reducing properties of which contributed to the controlled release of the drug and reduced toxicity to healthy tissues. The nanoparticles can achieve targeted release due to the presence of disulfide bonds found in the irinotecan derivative. Furthermore, the cytotoxicity assay showed a higher antitumor efficacy of the conjugate, compared to the free drug, against the two types of tumor cells tested.

The third strategy is the possibility of obtaining a (co)polymer, whose repeat units will have additional functional groups capable of binding to the drug molecule in a post-polymerization act, similar to the “grafting to” strategy shown in the example of polymer–protein conjugates. Chen et al. synthesized a redox-responsive polymer–drug conjugate based on a hydrophilic diblock copolymer covalently linked to a sulfide-bridged derivative of the anti-cancer drug PTX [283]. The hydrophilic diblock copolymer PEG-*b*-PHEMA was synthesized via the ATRP process using 2-(trimethylsilyloxy)ethyl methacrylate and PEG-Br as a macroinitiator to then, in the next step, selectively hydrolyze the trimethylsilane group to hydroxyl groups. Utilizing the generated hydroxyl functionalities, PTX was covalently coupled to the polymer matrix resulting in an LC of 18.4 wt%. The authors demonstrated the possibility of the self-assembly of the conjugate into spherical micelles in an aqueous solution, with hydrophobic paclitaxel as the core and hydrophilic PEG chains as the shell. Most importantly, the results of cytotoxicity indicate that the obtained conjugates can effectively inhibit the proliferation of tumor cells. Dong et al. designed the synthesis of a polymer–drug conjugate based on a diblock copolymer via the ATRP process of GMA with an initiator based on PEG-Br and the post-polymerization aldehyde modification and conjugation of DOX via an acid labile imine bond [284]. The amphiphilic conjugate can self-assemble into nanoparticles with a core-shell structure, whereas the PEG block is a hydrophilic shell and the block containing DOX is a hydrophobic core. The authors showed that the conjugate produced can effectively deliver the active substance to the cell nuclei and shows a more effective anticancer effect compared to that of free DOX.

Table 5 presents an overview of the literature on the synthesis of covalently bound polymer–drug conjugates using three strategies utilizing various ATRP techniques, depending on the type of therapeutic agent used and the composition of the polymer matrix. The table shows the work that has recently been put into the development of this type of conjugate, as well as the universality, commonness, and versatility of various variants of the ATRP technique.

## 7. Concluding Remarks

Although this review is limited to selected DDS-related papers published in the last decade, it demonstrates the enormous potential of ATRP in developing new classes of block copolymers, bioconjugates, and hybrid inorganic–organic particles that can serve as carriers for small drugs, proteins, and nucleic acids. Current research makes it possible to obtain smart carriers in the form of a wide range of stimuli-responsive micelles, polymersomes, polyplexes, hybrid inorganic–organic particles, or implantable wafers with a controlled delivery of selected active substances. One can expect that among these, the ones susceptible to the internal stimuli present in the human body subjected to a disease (e.g., pH changes or redox conditions) seem to have a particular potential for quick implementation in medical treatment due to their simplicity of use by patients (e.g., no or only limited requirements for specialized instrumentation, much less need to engage the patient’s or physician’s attention to control treatment conditions). Nevertheless, scaling up the synthesis of many of the described carriers may be a difficult problem and significant work should be directed toward more efficient, as well as less costly and time-consuming, procedures. As a result, it remains a very important task to develop new ATRP methods to effectively control the polymerization process using trace amounts of metal catalysts. Photochemical variants of metal-free ATRP also appear to be of interest, especially those in which the photo-initiator and the catalyst providing the balance between active and dormant species are fully biocompatible organic compounds (e.g., vitamin B2) [299]. It should also be noted that the RAFT polymerization technique has also been used extensively in drug delivery systems in recent years and offers a large library of alternative approaches, which can be applied to future technologies [300]. One of the next big challenges is to better understand the cytocompatibility of ATRP- and RAFT-based materials and to confirm their applicability in various animal models and ultimately in humans.

## Figures and Tables

**Table 1:** A review of the literature on the synthesis of modern polymersomes using ATRP techniques.

Encapsulated Drug	Monomers Used	The Variant of the ATRP Technique	Applications	Ref
PTX	MMA, DMAEMA	normal	pH-triggered drug release	[163]
DOX·HCl	MEMA, DEAEMA	normal	Ultrasound-triggered drug release	[164]
-	MMA, DMAEMA	ARGET	Gateway to stimuli-responsive giant hybrid vesicle DDS	[165]
-	ABOC, FBOC	normal	Gateway for pH-triggered DDS	[166]

**Table 2:** A review of the literature on the synthesis of modern polyplexes using normal ATRP technique.

Encapsulated Drug	Nucleic Acid Used	Monomers Used	Applications	Ref
-	pDNA	NIPAM	Thermosensitive nucleic acid delivery	[176]
-	siRNA	NIPAM	Hydrogel-aided nucleic acid delivery	[177]
DOX	DNA	DMAEMA	Redox-triggered drug and nucleic acid release	[178]
PTX	pDNA	DMAEMA	Bcl-2 targeted drug and nucleic acid delivery	[179]

**Table 3:** A review of the literature on the synthesis of modern polymer combs and brushes using ATRP techniques.

Encapsulated Drug/Nucleic Acid	Monomers Used	The Variant of the ATRP Technique	Applications	Ref
DEX	PEGMA	normal	Tuning molecular architecture for tailoring drug-releasing properties	[206]
DOX	OEGMA	normal	Redox-triggered drug release	[207]
DOX	PEGMA	normal	Redox-triggered drug release	[209,210]
-	ABMA	normal	Gateway for thermos-responsive drug release	[211]
pDNA	EGDMA, DMAEMA, β-Pinene	DE/AGET	Nucleic acid delivery	[212]
siRNA	OEOMA, GMA	ARGET	MOR-targeted nucleic acid delivery	[216]
pDNA	GMA	normal	pH-triggered nucleic acid delivery	[219]
DOX, siRNA	PEGMA	normal	Redox-triggered drug and nucleic acid release	[220]

**Table 4:** A review of the literature on the synthesis of modern polymer–protein conjugates using the “grafting from” strategy and ATRP techniques.

Type of Protein	Type of Monomers ^1^	The Variant of the ATRP Technique Used	Applications and Conclusions	Ref.
Chymotrypsin	CBMA, AMA	normal	Modifying the structure by adding a polymer significantly increased protein stability and reduced protein–protein interactions.	[253]
Chymotrypsin-α Trypsin	CBMA PEGMA	normal	Protein–polymer conjugates, which can exist as a prodrug until the activator is introduced, can be used in enzyme-based biosensors and drug delivery for cancer treatment.	[264]
Chymotrypsin-α	CBMA PEGMA 3-SPMA DMAEMA	normal	Covalently attached synthetic polymers are able to modulate protein folding, emulating molecular chaperones.	[265]
Human serum albumin	DPA	normal	Promising as a new class of tumor microenvironment responsive nanocarriers for improved tumor imaging and therapy.	[266]
Interferon-α	HPMAPEGMA	normal	Promising next-generation technology that will significantly improve the pharmacological performance of therapeutic proteins with a short circulating half-life.	[267]
Lysozyme	CBMA PEGMA	normal	The covalent attachment of polymers to a protein can significantly change the protein solubility, which can be adjusted by changing the polymer type, grafting density, and polymer length. Polymer attachment increases the resistance to unfavorable environments and the thermostability of the protein.	[268]
Horseradish peroxidase	ACR	AGET	The resulting conjugates essentially retained the catalytic properties of the protein and showed significantly improved thermal stability to high temperature and trypsin digestion.	[269]
Green fluorescent protein	PEGMA	ARGET	The protein retained its bio-fluorescent properties during the process, indicating the utility of ARGET ATRP for the preparation of protein–polymer conjugates.	[270]
Lipase	DMAPAA	ICAR	A ubiquitous class of amino acid residues can be modified by ATRP initiators without affecting enzyme activity. This new amino acid modification strategy can be applied to other enzymes, providing access to new biohybrid modification schemes.	[271]
Bovine serum albumin	OEOMA	Photo	The first example of photo-ATRP using blue LED irradiation in an aquatic environment. Compared to more energetic light sources, blue light is more friendly to biological systems and allows enzymes to survive and maintain their structure and functions.	[272]
Bovine serum albumin	MSEAM	PICAR	A new sulfoxide-functional acrylamide monomer was synthesized as an alternative to PEG in some biomedical applications. It was used in the PICAR ATRP process under biologically relevant conditions without degassing the reaction mixture.	[273]
β-barrel transmembrane	NIPAM	SARA	The first example of the use of a transmembrane protein in the production of conjugates by the “grafting from” strategy, using ATRP techniques. Thanks to the preserved pore geometry, transmembrane protein–polymer conjugates can be used as building blocks of functional polymer membranes, drug and gene carriers, and nanoreactors.	[274]

^1^ Monomer abbreviations not introduced in the text: carboxybetaine methacrylate (CBMA), azide methacrylate (AMA), 3-Sulfopropyl methacrylate (3-SPMA), 2-(diisopropylamino)ethyl methacrylate (DPA), 2-hydroxypropyl methacrylate (HPMA), acrylamide (ACR), N-[3-(N,N-dimethylamino)propyl] acrylamide (DMAPAA), 2-(methylsulfinyl)ethyl acrylamide (MSEAM).

**Table 5:** A review of the literature on the synthesis of polymer–drug conjugates using ATRP techniques.

Strategy	(Co)Polymers	Active Substance	Synthesis Techniques	Ref.
Active substance as an ATRP initiator	Poly(carboxybetaine)	CPT	ATRP	[278]
	Poly(methacryloyloxyethyl phosphorylcholine)	CPT	ATRP	[68]
	Poly(oligo(ethylene oxide) methacrylate)-b-(glycidyl methacrylate)	Fentanyl	AGET ATRP	[216]
	Poly(di(ethylene glycol) methyl ether methacrylate) Poly(di(ethylene glycol) methyl ether methacrylate)-b-poly(methyl methacrylate)	Inositol (vitamin B_8_)	ARGET ATRP, SARA ATRP, seATRP	[183]
	Poly(methyl methacrylate-co-2-hydroxyethyl methacrylate)	Retinol (vitamin A)	ATRP	[285]
	Poly(n-butyl acrylate) Poly(methyl methacrylate) Poly(N-isopropylacrylamide) Poly(N-isopropylacrylamide)-b-poly(oligo(ethylene glycol) acrylate) Poly(N-isopropylacrylamide)-b-poly(2-hydroxyethyl acrylate)	Riboflavin (vitamin B_2_)	ARGET ATRP, Metal-free ATRP, Photo ATRP, seATRP	[286]
Active substance as (meth)acrylate monomer	Poly(lactic acid)-b-poly(camptothecin mono-2-(methacryloyloxy)ethyl succinate) Poly(lactic acid)-b-poly(camptothecin mono-2-(methacryloyloxy)ethyl succinate-co- poly(ethylene glycol) methyl ether methacrylate)	CPT	ATRP	[281,287]
	Poly(hydroxypropyl methacrylate-co-Methacryloyloxy-3-thiohexanoyl camptothecin-co-2-(2′-Bromoisobutyryloxy)ethyl-2′’-methacryloyl oxyethyl disulfide) Poly(hydroxypropyl methacrylate-co-Methacryloyloxy-3-thiohexanoyl camptothecin-co-2-(2′-Bromoisobutyryloxy)ethyl-2′’-methacryloyl oxyethyl disulfide)(poly(poly(ethylene glycol) methyl ether methacrylate))	CPT	ATRP	[288]
	Cellulose-g-poly(methacrylate derivative of camptothecin)-b- poly(ethylene glycol) methyl ether methacrylate)	CPT	ATRP	[289]
	Dextran-poly(methacrylate derivative of camptothecin)-b-poly(ethylene glycol) methyl ether methacrylate)	CPT	ATRP	[290]
	α-cyclodextrin- poly(ethylene glycol) polyrotaxanes-poly(methacrylate derivative of camptothecin)-b-poly(ethylene glycol) methyl ether methacrylate)	CPT	ATRP	[291]
	Poly(ethylene glycol)-b-poly(2-([2-4-(2-methylpropil)phenyl]propionyl]oxy)ethyl methacrylate	Ibuprofen	ATRP	[96]
	β-cyclodextrin-poly(methacrylate derivative of irinotecan-co-poly(ethylene glycol) methyl ether methacrylate)	Irinotecan	ATRP	[282]
Post-polymerization conjugation	Poly(methacryloyloxyethyl phosphorylcholine)-graft-camptothecin	CPT	ATRP, Click Chemistry	[68]
	Poly(glycidyl methacrylate) Poly(poly(ethylene glycol) methyl ether methacrylate-co-glycidyl methacrylate)	Ciprofloxacin	AGET ATRP, ICAR ATRP, ROP, Click Chemistry	[218]
	Poly(methacryloyloxyethyl phosphorylcholine)-graft-doxorubicin Poly(methacryloyloxyethyl phosphorylcholine-co-2-tert-butoxy-2-oxoethyl methacrylate)	DOX	ATRP, Click Chemistry, Acylhydrazine formation	[292]
	Poly(poly(ethylene glycol) methacrylate)−b- poly(caprolactone)−b-poly(poly(ethylene glycol) methacrylate)	DOX	ATRP, Acylhydrazine formation	[293]
	Poly(methacryloyloxyethyl phosphorylcholine)-b-poly(2-methoxy-2-oxoethyl methacrylate)	DOX	ATRP, Acylhydrazine formation	[294]
	Poly(ethylene oxide)-b-poly(glycidyl methacrylate)	DOX	ATRP, Imine formation	[284]
	Poly(2-(2-bromoisobutyryloxy)ethyl methacrylate)-co-poly[poly(ethylene glycol) methacrylate-co-3-vinyl benzaldehyde]	DOX	ATRP, Imine formation	[295]
	Poly(methacrylic acid)	Estradiol Tamoxifen	ATRP, N-alkylation of amines with carboxylic acid	[296]
	Poly(ethylene oxide)-b-poly- (n-butyl methacrylate-co-4-methyl-[7-(methacryloyl)-oxyethyloxy]coumarin))	5-fluorouracil	ATRP, Photochemically induced [2 + 2] cycloaddition reaction	[297]
	Poly(ethylene oxide)-b-poly(glycerol monomethacrylate)	Indomethacin	ATRP, Steglich esterification	[298]
	Poly(ethylene glycol)-b-poly(2-(trimethylsilyloxyl) ethyl methacrylate)	Paclitaxel	ATRP, Esterification	[283]

## Data Availability

Not applicable.

## References

[1] Widder K.J. (1983). Controlled Release Delivery Systems.

[2] Uhrich K.E., Cannizzaro S.M., Langer R.S., Shakesheff K.M. (1999). Polymeric systems for controlled drug release. Chem. Rev. Columb..

[3] Duncan R. (2003). The dawning era of polymer therapeutics. Nat. Rev. Drug. Discov..

[4] Garnett M.C. (2001). Targeted drug conjugates: Principles and progress. Adv. Drug Deliv. Rev..

[5] Greco F., Vicent M.J. (2008). Polymer-drug conjugates: Current status and future trends. Front. Biosci.-Landmark.

[6] Pasut G., Veronese F. (2007). Polymer–drug conjugation, recent achievements and general strategies. Prog. Polym. Sci..

[7] Goodman L.S., Gilman A.G. (2017). Goodman & Gilman’s Pharmacological Basis of Therapeutics.

[8] Elsabahy M., Wooley K.L. (2012). Design of polymeric nanoparticles for biomedical delivery applications. Chem. Soc. Rev..

[9] Hubbell J.A., Chilkoti A. (2012). Nanomaterials for drug delivery. Science.

[10] Kowalczuk A., Trzcinska R., Trzebicka B., Müller A.H., Dworak A., Tsvetanov C.B. (2014). Loading of polymer nanocarriers: Factors, mechanisms and applications. Prog. Polym. Sci..

[11] Jahangirian H., Lemraski E.G., Webster T.J., Rafiee-Moghaddam R., Abdollahi Y. (2017). A review of drug delivery systems based on nanotechnology and green chemistry: Green nanomedicine. Int. J. Nanomed..

[12] Deng C., Jiang Y., Cheng R., Meng F., Zhong Z. (2012). Biodegradable polymeric micelles for targeted and controlled anticancer drug delivery: Promises, progress and prospects. Nano Today.

[13] Wei H., Zhuo R.-X., Zhang X.-Z. (2013). Design and development of polymeric micelles with cleavable links for intracellular drug delivery. Prog. Polym. Sci..

[14] Lee J.S., Feijen J. (2012). Polymersomes for drug delivery: Design, formation and characterization. J. Control. Release.

[15] Lefley J., Waldron C., Becer C.R. (2020). Macromolecular design and preparation of polymersomes. Polym. Chem..

[16] Chauhan A.S. (2018). Dendrimers for drug delivery. Molecules.

[17] Chacko R.T., Ventura J., Zhuang J., Thayumanavan S. (2012). Polymer nanogels: A versatile nanoscopic drug delivery platform. Adv. Drug Deliv. Rev..

[18] Anglin E.J., Cheng L., Freeman W.R., Sailor M.J. (2008). Porous silicon in drug delivery devices and materials. Adv. Drug Deliv. Rev..

[19] Vallet-Regí M., Balas F., Arcos D. (2007). Mesoporous materials for drug delivery. Angew. Chem. Int. Ed..

[20] Li Z., Barnes J.C., Bosoy A., Stoddart J.F., Zink J.I. (2012). Mesoporous silica nanoparticles in biomedical applications. Chem. Soc. Rev..

[21] Fouassier J., Allonas X., Lalevée J. (2022). Macromolecular Engineering: From Precise Macromolecular Synthesis to Macroscopic Materials Properties and Applications.

[22] Corbin D.A., Miyake G.M. (2021). Photoinduced organocatalyzed atom transfer radical polymerization (O-ATRP): Precision polymer synthesis using organic photoredox catalysis. Chem. Rev..

[23] Kreutzer J. (2018). Atom-transfer radical polymerization: New method breathes life into ATRP. Nat. Rev. Chem..

[24] Pan X., Fantin M., Yuan F., Matyjaszewski K. (2018). Externally controlled atom transfer radical polymerization. Chem. Soc. Rev..

[25] Chmielarz P., Fantin M., Park S., Isse A.A., Gennaro A., Magenau A.J., Sobkowiak A., Matyjaszewski K. (2017). Electrochemically mediated atom transfer radical polymerization (eATRP). Prog. Polym. Sci..

[26] Dadashi-Silab S., Atilla Tasdelen M., Yagci Y. (2014). Photoinitiated atom transfer radical polymerization: Current status and future perspectives. J. Polym. Sci. Part A Polym. Chem..

[27] Tsarevsky N.V., Matyjaszewski K. (2013). Atom transfer radical polymerization (ATRP). Fundamentals of Conrolled/Living Radical Polymerization.

[28] Ayres N. (2011). Atom transfer radical polymerization: A robust and versatile route for polymer synthesis. Polym. Rev..

[29] Pintauer T., Matyjaszewski K. (2008). Atom transfer radical addition and polymerization reactions catalyzed by ppm amounts of copper complexes. Chem. Soc. Rev..

[30] Tsarevsky N.V., Matyjaszewski K. (2007). “Green” atom transfer radical polymerization: From process design to preparation of well-defined environmentally friendly polymeric materials. Chem. Rev..

[31] Faucher S., Zhu S. (2007). Fundamentals and development of high-efficiency supported catalyst systems for atom transfer radical polymerization. J. Polym. Sci. Part A Polym. Chem..

[32] Tsarevsky N.V., Matyjaszewski K. (2006). Environmentally benign atom transfer radical polymerization: Towards “green” processes and materials. J. Polym. Sci. Part A Polym. Chem..

[33] Matyjaszewski K., Xia J. (2001). Atom transfer radical polymerization. Chem. Rev..

[34] Boyer C., Bulmus V., Davis T.P., Ladmiral V., Liu J., Perrier S. (2009). Bioapplications of RAFT polymerization. Chem. Rev..

[35] Siegwart D.J., Oh J.K., Matyjaszewski K. (2012). ATRP in the design of functional materials for biomedical applications. Prog. Polym. Sci..

[36] Wang J.S., Matyjaszewski K. (1995). Controlled/“living” radical polymerization. atom transfer radical polymerization in the presence of transition-metal complexes. J. Am. Chem. Soc..

[37] Beers K.L. (2020). The first dive into the mechanism and kinetics of ATRP. Macromolecules.

[38] Curran D.P. (2002). The Design and Application of Free Radical Chain Reactions in Organic Synthesis. Part 1. Synthesis.

[39] Hawker C.J., Barclay G.G., Orellana A., Dao J., Devonport W. (1996). Initiating systems for nitroxide-mediated “living” free radical polymerizations: Synthesis and evaluation. Macromolecules.

[40] Goto A., Sato K., Tsujii Y., Fukuda T., Moad G., Rizzardo E., Thang S.H. (2001). Mechanism and kinetics of RAFT-based living radical polymerizations of styrene and methyl methacrylate. Macromolecules.

[41] Zhou Y.-N., Li J.-J., Wang T.-T., Wu Y.-Y., Luo Z.-H. (2022). Precision Polymer Synthesis by Controlled Radical Polymerization: Fusing the progress from Polymer Chemistry and Reaction Engineering. Prog. Polym. Sci..

[42] Matyjaszewski K., Xia J., Matyjaszewski K.D., Thomas P. (2002). Fundamentals of Atom Transfer Radical Polymerization. Handbook of Radical Polymerization.

[43] Lorandi F., Fantin M., Matyjaszewski K. (2022). Atom Transfer Radical Polymerization: A Mechanistic Perspective. J. Am. Chem. Soc..

[44] Fung A.K., Coote M.L. (2021). A mechanistic perspective on atom transfer radical polymerization. Polym. Int..

[45] Xia J., Zhang X., Matyjaszewski K. (2000). The effect of ligands on copper-mediated atom transfer radical polymerization. Transition Metal Catalysis in Macromolecular Design.

[46] Matyjaszewski K., Göbelt B., Paik H.-j., Horwitz C.P. (2001). Tridentate nitrogen-based ligands in Cu-based ATRP: A structure− activity study. Macromolecules.

[47] Chen X.P., Qiu K.Y. (2000). ‘Living’radical polymerization of styrene with AIBN/FeCl3/PPh3 initiating system via a reverse atom transfer radical polymerization process. Polym. Int..

[48] Dadashi-Silab S., Matyjaszewski K. (2020). Iron catalysts in atom transfer radical polymerization. Molecules.

[49] Zhu S., Xiao G., Yan D. (2001). Synthesis of aromatic polyethersulfone-based graft copolyacrylates via ATRP catalyzed by FeCl2/isophthalic acid. J. Polym. Sci. Part A Polym. Chem..

[50] Xue Z., He D., Xie X. (2015). Iron-catalyzed atom transfer radical polymerization. Polym. Chem..

[51] Plichta A., Li W., Matyjaszewski K. (2009). ICAR ATRP of styrene and methyl methacrylate with Ru (Cp*) Cl (PPh3) 2. Macromolecules.

[52] He D., Noh S.K., Lyoo W.S. (2011). In situ-generated Ru (III)-mediated ATRP from the polymeric Ru (III) complex in the absence of activator generation agents. J. Polym. Sci. Part A Polym. Chem..

[53] Yang X., Yu Y., Lai Q., Yang X., Luo P., Zhang B., Zhang X., Wei Y. (2022). Recent development and advances on fabrication and biomedical applications of Ga-based liquid metal micro/nanoparticles. Compos. Part B Eng..

[54] Noto N., Saito S. (2022). Arylamines as More Strongly Reducing Organic Photoredox Catalysts than fac-[Ir (ppy) 3]. ACS Catal..

[55] Bai L., Zhang L., Cheng Z., Zhu X. (2012). Activators generated by electron transfer for atom transfer radical polymerization: Recent advances in catalyst and polymer chemistry. Polym. Chem..

[56] Mueller L., Jakubowski W., Tang W., Matyjaszewski K. (2007). Successful chain extension of polyacrylate and polystyrene macroinitiators with methacrylates in an ARGET and ICAR ATRP. Macromolecules.

[57] Zhang L., Miao J., Cheng Z., Zhu X. (2010). Iron-Mediated ICAR ATRP of Styrene and Methyl Methacrylate in the Absence of Thermal Radical Initiator. Macromol. Rapid Commun..

[58] Konkolewicz D., Wang Y., Zhong M., Krys P., Isse A.A., Gennaro A., Matyjaszewski K. (2013). Reversible-deactivation radical polymerization in the presence of metallic copper. A critical assessment of the SARA ATRP and SET-LRP mechanisms. Macromolecules.

[59] Matyjaszewski K., Magenau A., Gennaro A., Strandwitz N.C. (2017). Electrochemically mediated atom transfer radical polymerization. Science.

[60] Mohapatra H., Kleiman M., Esser-Kahn A.P. (2017). Mechanically controlled radical polymerization initiated by ultrasound. Nat. Chem..

[61] Wang Z., Wang Z., Pan X., Fu L., Lathwal S., Olszewski M., Yan J., Enciso A.E., Wang Z., Xia H. (2018). Ultrasonication-induced aqueous atom transfer radical polymerization. ACS Macro Lett..

[62] Soly S., Mistry B., Murthy C. (2022). Photo-mediated metal-free atom transfer radical polymerization: Recent advances in organocatalysts and perfection towards polymer synthesis. Polym. Int..

[63] Dworakowska S., Lorandi F., Gorczyński A., Matyjaszewski K. (2022). Toward Green Atom Transfer Radical Polymerization: Current Status and Future Challenges. Adv. Sci..

[64] Berbigier J.F., Teixeira Alves Duarte L.G., Zawacki M.F., de Araujo B.B., Moura Santos C.d., Atvars T.D.Z., Gonçalves P.F.B., Petzhold C.L., Rodembusch F.S. (2020). ATRP initiators based on proton transfer benzazole dyes: Solid-state photoactive polymer with very large Stokes shift. ACS Appl. Polym. Mater..

[65] Qi X., Yan H., Li Y. (2021). ATRP-based synthesis of a pH-sensitive amphiphilic block polymer and its self-assembled micelles with hollow mesoporous silica as DOX carriers for controlled drug release. RSC Adv..

[66] Sumerlin B.S. (2012). Proteins as initiators of controlled radical polymerization: Grafting-from via ATRP and RAFT. ACS Macro Lett..

[67] Messina M.S., Messina K.M.M., Bhattacharya A., Montgomery H.R., Maynard H.D. (2020). Preparation of biomolecule-polymer conjugates by grafting-from using ATRP, RAFT, or ROMP. Prog. Polym. Sci..

[68] Chen X., McRae S., Parelkar S., Emrick T. (2009). Polymeric Phosphorylcholine−Camptothecin Conjugates Prepared by Controlled Free Radical Polymerization and Click Chemistry. Bioconjugate Chem..

[69] Cheng-Mei L., Rui B., Jin-Jun Q., Fen H., Yan X., Chen Z., Yun Z. (2006). Coumarin end-capped polystyrene by ATRP and photodimerization reaction. Polym. Bull..

[70] Mansfeld U., Pietsch C., Hoogenboom R., Becer C.R., Schubert U.S. (2010). Clickable initiators, monomers and polymers in controlled radical polymerizations—A prospective combination in polymer science. Polym. Chem..

[71] Coad B.R., Styan K.E., Meagher L. (2014). One step ATRP initiator immobilization on surfaces leading to gradient-grafted polymer brushes. ACS Appl. Mater. Interfaces.

[72] Ślusarczyk K., Flejszar M., Chmielarz P. (2021). Less is more: A review of μL-scale of SI-ATRP in polymer brushes synthesis. Polymer.

[73] Matyjaszewski K. (2018). Advanced materials by atom transfer radical polymerization. Adv. Mater..

[74] Boyce J.R., Shirvanyants D., Sheiko S.S., Ivanov D.A., Qin S., Börner H., Matyjaszewski K. (2004). Multiarm molecular brushes: Effect of the number of arms on the molecular weight polydispersity and surface ordering. Langmuir.

[75] Nasrullah M.J., Vora A., Webster D.C. (2011). Block copolymer synthesis via a combination of ATRP and RAFT using click chemistry. Macromol. Chem. Phys..

[76] Dau H., Jones G.R., Tsogtgerel E., Nguyen D., Keyes A., Liu Y.-S., Rauf H., Ordonez E., Puchelle V., Basbug Alhan H. (2022). Linear block copolymer synthesis. Chem. Rev..

[77] Plichta A., Jaskulski T., Lisowska P., Macios K., Kundys A. (2015). Elastic polyesters improved by ATRP as reactive epoxy-modifiers of PLA. Polymer.

[78] Karayianni M., Pispas S. (2021). Block copolymer solution self-assembly: Recent advances, emerging trends, and applications. J. Polym. Sci..

[79] Plichta A., Zhong M., Li W., Elsen A.M., Matyjaszewski K. (2012). Tuning dispersity in diblock copolymers using ARGET ATRP. Macromol. Chem. Phys..

[80] Listak J., Jia X., Plichta A., Zhong M., Matyjaszewski K., Bockstaller M.R. (2012). Effect of block molecular weight distribution on the structure formation in block copolymer/homopolymer blends. J. Polym. Sci. Part B Polym. Phys..

[81] Listak J., Jakubowski W., Mueller L., Plichta A., Matyjaszewski K., Bockstaller M.R. (2008). Effect of symmetry of molecular weight distribution in block copolymers on formation of “metastable” morphologies. Macromolecules.

[82] Oliveira A.S., Mendonça P.V., Simões S., Serra A.C., Coelho J.F. (2021). Amphiphilic well-defined degradable star block copolymers by combination of ring-opening polymerization and atom transfer radical polymerization: Synthesis and application as drug delivery carriers. J. Polym. Sci..

[83] Mühlebach A., Gaynor S.G., Matyjaszewski K. (1998). Synthesis of amphiphilic block copolymers by atom transfer radical polymerization (ATRP). Macromolecules.

[84] Hua M., Kaneko T., Liu X.-Y., Chen M.-q., Akashi M. (2005). Successful ATRP syntheses of amphiphilic block copolymers poly (styrene-block-N, N-dimethylacrylamide) and their self-assembly. Polym. J..

[85] Wei H., Perrier S., Dehn S., Ravarian R., Dehghani F. (2012). One-pot ATRP synthesis of a triple hydrophilic block copolymer with dual LCSTs and its thermo-induced association behavior. Soft Matter.

[86] Kashapov R., Gaynanova G., Gabdrakhmanov D., Kuznetsov D., Pavlov R., Petrov K., Zakharova L., Sinyashin O. (2020). Self-assembly of amphiphilic compounds as a versatile tool for construction of nanoscale drug carriers. Int. J. Mol. Sci..

[87] He W., Jiang H., Zhang L., Cheng Z., Zhu X. (2013). Atom transfer radical polymerization of hydrophilic monomers and its applications. Polym. Chem..

[88] Colombani O., Ruppel M., Schubert F., Zettl H., Pergushov D.V., Müller A.H. (2007). Synthesis of poly (n-butyl acrylate)-block-poly (acrylic acid) diblock copolymers by ATRP and their micellization in water. Macromolecules.

[89] Kuperkar K., Patel D., Atanase L.I., Bahadur P. (2022). Amphiphilic Block Copolymers: Their Structures, and Self-Assembly to Polymeric Micelles and Polymersomes as Drug Delivery Vehicles. Polymers.

[90] Feng H., Lu X., Wang W., Kang N.-G., Mays J.W. (2017). Block copolymers: Synthesis, self-assembly, and applications. Polymers.

[91] Prasad P.V., Purkayastha K., Sharma U., Barik M. (2020). Ph-sensitive nanomedicine for treating gynaecological cancers. J. Womans Reprod. Health.

[92] Cabral H., Kataoka K. (2014). Progress of drug-loaded polymeric micelles into clinical studies. J. Control. Release.

[93] Wang G., Zhang L. (2016). Synthesis, self-assembly and pH sensitivity of PDEAEMA–PEG–PDEAEMA triblock copolymer micelles for drug delivery. React. Funct. Polym..

[94] Zhang L., Zhang C., Gu X., Wang G. (2021). Self-assembly, pH-responsibility and controlled release of doxorubicin of PDEAEMA-PEG-PDEAEMA triblock copolymers: Effects of PEG length. J. Polym. Res..

[95] Biswas D., An S.Y., Li Y., Wang X., Oh J.K. (2017). Intracellular delivery of colloidally stable core-cross-linked triblock copolymer micelles with glutathione-responsive enhanced drug release for cancer therapy. Mol. Pharm..

[96] Zeng Z., Wei Z., Ma L., Xu Y., Xing Z., Niu H., Wang H., Huang W. (2017). pH-Responsive nanoparticles based on ibuprofen prodrug as drug carriers for inhibition of primary tumor growth and metastasis. J. Mater. Chem. B.

[97] Zhang X., Yuan T., Dong H., Xu J., Wang D., Tong H., Ji X., Sun B., Zhu M., Jiang X. (2018). Novel block glycopolymers prepared as delivery nanocarriers for controlled release of bortezomib. Colloid Polym. Sci..

[98] Yang C., Liu W., Xiao J., Yuan C., Chen Y., Guo J., Yue H., Zhu D., Lin W., Tang S. (2020). pH-sensitive mixed micelles assembled from PDEAEMA-PPEGMA and PCL-PPEGMA for doxorubicin delivery: Experimental and DPD simulations study. Pharmaceutics.

[99] Hao D., Zhang Z., Ji Y. (2022). Responsive polymeric drug delivery systems for combination anticancer therapy: Experimental design and computational insights. Int. J. Polym. Mater. Polym. Biomater..

[100] Chu S., Shi X., Tian Y., Gao F. (2022). pH-Responsive Polymer Nanomaterials for Tumor Therapy. Front. Oncol..

[101] Koltai T. (2020). The Ph paradigm in cancer. Eur. J. Clin. Nutr..

[102] Kumar R., Santa Chalarca C.F., Bockman M.R., Bruggen C.V., Grimme C.J., Dalal R.J., Hanson M.G., Hexum J.K., Reineke T.M. (2021). Polymeric delivery of therapeutic nucleic acids. Chem. Rev..

[103] Kennedy L., Sandhu J.K., Harper M.-E., Cuperlovic-Culf M. (2020). Role of glutathione in cancer: From mechanisms to therapies. Biomolecules.

[104] Casado N., Hernandez G., Sardon H., Mecerreyes D. (2016). Current trends in redox polymers for energy and medicine. Prog. Polym. Sci..

[105] Chen Q., Lin W., Wang H., Wang J., Zhang L. (2016). PDEAEMA-based pH-sensitive amphiphilic pentablock copolymers for controlled anticancer drug delivery. RSC Adv..

[106] Chen Q., Zheng J., Yuan X., Wang J., Zhang L. (2018). Folic acid grafted and tertiary amino based pH-responsive pentablock polymeric micelles for targeting anticancer drug delivery. Mater. Sci. Eng. C.

[107] Yang C., Xiao J., Xiao W., Lin W., Chen J., Chen Q., Zhang L., Zhang C., Guo J. (2017). Fabrication of PDEAEMA-based pH-responsive mixed micelles for application in controlled doxorubicin release. RSC Adv..

[108] Li Y., Yu A., Li L., Zhai G. (2018). The development of stimuli-responsive polymeric micelles for effective delivery of chemotherapeutic agents. J. Drug Target..

[109] Jazani A.M., Oh J.K. (2020). Development and disassembly of single and multiple acid-cleavable block copolymer nanoassemblies for drug delivery. Polym. Chem..

[110] Hu X., Jazani A.M., Oh J.K. (2021). Recent advances in development of imine-based acid-degradable polymeric nanoassemblies for intracellular drug delivery. Polymer.

[111] Gannimani R., Walvekar P., Naidu V.R., Aminabhavi T.M., Govender T. (2020). Acetal containing polymers as pH-responsive nano-drug delivery systems. J. Control. Release.

[112] Zhang L.-J., Dong B.-T., Du F.-S., Li Z.-C. (2012). Degradable thermoresponsive polyesters by atom transfer radical polyaddition and click chemistry. Macromolecules.

[113] Qiao Z.-Y., Ji R., Huang X.-N., Du F.-S., Zhang R., Liang D.-H., Li Z.-C. (2013). Polymersomes from dual responsive block copolymers: Drug encapsulation by heating and acid-triggered release. Biomacromolecules.

[114] Zheng L., Zhang X., Wang Y., Liu F., Peng J., Zhao X., Yang H., Ma L., Wang B., Chang C. (2018). Fabrication of acidic pH-cleavable polymer for anticancer drug delivery using a dual functional monomer. Biomacromolecules.

[115] Khorsand B., Oh J.K. (2013). pH-responsive destabilization and facile bioconjugation of new hydroxyl-terminated block copolymer micelles. J. Polym. Sci. Part A Polym. Chem..

[116] Cao H., Chen C., Xie D., Chen X., Wang P., Wang Y., Song H., Wang W. (2018). A hyperbranched amphiphilic acetal polymer for pH-sensitive drug delivery. Polym. Chem..

[117] Song C.-C., Su C.-C., Cheng J., Du F.-S., Liang D.-H., Li Z.-C. (2013). Toward tertiary amine-modulated acid-triggered hydrolysis of copolymers containing pendent ortho ester groups. Macromolecules.

[118] Deng H., Zhao X., Liu J., Zhang J., Deng L., Liu J., Dong A. (2016). Synergistic dual-pH responsive copolymer micelles for pH-dependent drug release. Nanoscale.

[119] Fang Y., Xue J., Gao S., Lu A., Yang D., Jiang H., He Y., Shi K. (2017). Cleavable PEGylation: A strategy for overcoming the “PEG dilemma” in efficient drug delivery. Drug Deliv..

[120] Zalba S., Ten Hagen T.L., Burgui C., Garrido M.J. (2022). Stealth nanoparticles in oncology: Facing the PEG dilemma. J. Control. Release.

[121] Patil S.S., Wadgaonkar P.P. (2017). Temperature and pH dual stimuli responsive PCL-b-PNIPAA m block copolymer assemblies and the cargo release studies. J. Polym. Sci. Part A Polym. Chem..

[122] Babikova D., Kalinova R., Momekova D., Ugrinova I., Momekov G., Dimitrov I. (2019). Multifunctional polymer nanocarrier for efficient targeted cellular and subcellular anticancer drug delivery. ACS Biomater. Sci. Eng..

[123] Jazani A.M., Shetty C., Movasat H., Bawa K.K., Oh J.K. (2021). Imidazole-Mediated Dual Location Disassembly of Acid-Degradable Intracellular Drug Delivery Block Copolymer Nanoassemblies. Macromol. Rapid Commun..

[124] Hao Y., He J., Li S., Liu J., Zhang M., Ni P. (2014). Synthesis of an acid-cleavable and fluorescent amphiphilic block copolymer as a combined delivery vector of DNA and doxorubicin. J. Mater. Chem. B.

[125] Babikova D., Kalinova R., Zhelezova I., Momekova D., Konstantinov S., Momekov G., Dimitrov I. (2016). Functional block copolymer nanocarriers for anticancer drug delivery. RSC Adv..

[126] Kamenova K., Grancharov G., Kortenova V., Petrov P.D. (2022). Redox-Responsive Crosslinked Mixed Micelles for Controllable Release of Caffeic Acid Phenethyl Ester. Pharmaceutics.

[127] Mirhadi E., Mashreghi M., Maleki M.F., Alavizadeh S.H., Arabi L., Badiee A., Jaafari M.R. (2020). Redox-sensitive nanoscale drug delivery systems for cancer treatment. Int. J. Pharm..

[128] Sun W., Yang Y. (2022). Recent advances in redox-responsive nanoparticles for combined cancer therapy. Nanoscale Adv..

[129] Zhang M., Song C.-C., Ji R., Qiao Z.-Y., Yang C., Qiu F.-Y., Liang D.-H., Du F.-S., Li Z.-C. (2016). Oxidation and temperature dual responsive polymers based on phenylboronic acid and N-isopropylacrylamide motifs. Polym. Chem..

[130] Zhang M., Song C.-C., Su S., Du F.-S., Li Z.-C. (2018). ROS-activated ratiometric fluorescent polymeric nanoparticles for self-reporting drug delivery. ACS Appl. Mater. Interfaces.

[131] Stubelius A., Lee S., Almutairi A. (2019). The chemistry of boronic acids in nanomaterials for drug delivery. Acc. Chem. Res..

[132] Song C.-C., Ji R., Du F.-S., Liang D.-H., Li Z.-C. (2013). Oxidation-accelerated hydrolysis of the ortho ester-containing acid-labile polymers. ACS Macro Lett..

[133] Hermanson G. (2013). The Reactions of Bioconjugation. Bioconjugate Techniques.

[134] Oh J.K. (2019). Disassembly and tumor-targeting drug delivery of reduction-responsive degradable block copolymer nanoassemblies. Polym. Chem..

[135] Ko N.R., Oh J.K. (2014). Glutathione-triggered disassembly of dual disulfide located degradable nanocarriers of polylactide-based block copolymers for rapid drug release. Biomacromolecules.

[136] Chan N., An S.Y., Oh J.K. (2014). Dual location disulfide degradable interlayer-crosslinked micelles with extended sheddable coronas exhibiting enhanced colloidal stability and rapid release. Polym. Chem..

[137] Liu Y.-S., Huang S.-J., Huang X.-S., Wu Y.-T., Chen H.-Y., Lo Y.-L., Wang L.-F. (2016). The synthesis and comparison of poly (methacrylic acid)–poly (ε-caprolactone) block copolymers with and without symmetrical disulfide linkages in the center for enhanced cellular uptake. RSC Adv..

[138] Lo Y.-L., Huang X.-S., Chen H.-Y., Huang Y.-C., Liao Z.-X., Wang L.-F. (2021). ROP and ATRP fabricated redox sensitive micelles based on PCL-SS-PMAA diblock copolymers to co-deliver PTX and CDDP for lung cancer therapy. Colloids Surf. B Biointerfaces.

[139] Li S.-X., Liu L., Zhang L.-J., Wu B., Wang C.-X., Zhou W., Zhuo R.-X., Huang S.-W. (2016). Synergetic enhancement of antitumor efficacy with charge-reversal and reduction-sensitive polymer micelles. Polym. Chem..

[140] Ko N.R., Cheong J., Noronha A., Wilds C.J., Oh J.K. (2015). Reductively-sheddable cationic nanocarriers for dual chemotherapy and gene therapy with enhanced release. Colloids Surf. B Biointerfaces.

[141] Zhang Q., Ko N.R., Oh J.K. (2012). Modulated morphologies and tunable thiol-responsive shedding of aqueous block copolymer aggregates. RSC Adv..

[142] An S.Y., Hong S.H., Tang C., Oh J.K. (2016). Rosin-based block copolymer intracellular delivery nanocarriers with reduction-responsive sheddable coronas for cancer therapy. Polym. Chem..

[143] Zhang Q., Aleksanian S., Noh S.M., Oh J.K. (2013). Thiol-responsive block copolymer nanocarriers exhibiting tunable release with morphology changes. Polym. Chem..

[144] Khorsand B., Lapointe G., Brett C., Oh J.K. (2013). Intracellular drug delivery nanocarriers of glutathione-responsive degradable block copolymers having pendant disulfide linkages. Biomacromolecules.

[145] Kumar P., Behl G., Kaur S., Yadav N., Liu B., Chhikara A. (2021). Tumor microenvironment responsive nanogels as a smart triggered release platform for enhanced intracellular delivery of doxorubicin. J. Biomater. Sci. Polym. Ed..

[146] Tian K., Jia X., Zhao X., Liu P. (2016). pH/Reductant Dual-Responsive Core-Cross-Linked Micelles via Facile in Situ ATRP for Tumor-Targeted Delivery of Anticancer Drug with Enhanced Anticancer Efficiency. Mol. Pharm..

[147] Chan N., Khorsand B., Aleksanian S., Oh J.K. (2013). A dual location stimuli-responsive degradation strategy of block copolymer nanocarriers for accelerated release. Chem. Commun..

[148] Huang Y., Moini Jazani A., Howell E.P., Oh J.K., Moffitt M.G. (2019). Controlled Microfluidic Synthesis of Biological Stimuli-Responsive Polymer Nanoparticles. ACS Appl. Mater. Interfaces.

[149] Huang Y., Jazani A.M., Howell E.P., Reynolds L.A., Oh J.K., Moffitt M.G. (2020). Microfluidic Shear Processing Control of Biological Reduction Stimuli-Responsive Polymer Nanoparticles for Drug Delivery. ACS Biomater. Sci. Eng..

[150] Jazani A.M., Oh J.K. (2017). Dual location, dual acidic pH/reduction-responsive degradable block copolymer: Synthesis and investigation of ketal linkage instability under ATRP conditions. Macromolecules.

[151] Shetty C., Noronha A., Pontarelli A., Wilds C.J., Oh J.K. (2020). Dual-location dual-acid/glutathione-degradable cationic micelleplexes through hydrophobic modification for enhanced gene silencing. Mol. Pharm..

[152] Akimoto J., Ito Y., Okano T., Nakayama M. (2018). Controlled aggregation behavior of thermoresponsive polymeric micelles by introducing hydrophilic segments as corona components. J. Polym. Sci. Part A Polym. Chem..

[153] Dong Y., Ma X., Huo H., Zhang Q., Qu F., Chen F. (2018). Preparation of quadruple responsive polymeric micelles combining temperature-, pH-, redox-, and UV-responsive behaviors and its application in controlled release system. J. Appl. Polym. Sci..

[154] Ma X., Liu J., Lei L., Yang H., Lei Z. (2019). Synthesis of light and dual-redox triple-stimuli-responsive core-crosslinked micelles as nanocarriers for controlled release. J. Appl. Polym. Sci..

[155] Yuan W., Guo W. (2014). Ultraviolet light-breakable and tunable thermoresponsive amphiphilic block copolymer: From self-assembly, disassembly to re-self-assembly. Polym. Chem..

[156] Jazani A.M., Oh J.K. (2022). Synthesis of multiple stimuli-responsive degradable block copolymers via facile carbonyl imidazole-induced postpolymerization modification. Polym. Chem..

[157] Sharma A.K., Prasher P., Aljabali A.A., Mishra V., Gandhi H., Kumar S., Mutalik S., Chellappan D.K., Tambuwala M.M., Dua K. (2020). Emerging era of “somes”: Polymersomes as versatile drug delivery carrier for cancer diagnostics and therapy. Drug Deliv. Transl. Res..

[158] Trombino S., Curcio F., Cassano R. (2022). Polymersomes as a promising vehicle for controlled drug delivery. Stimuli-Responsive Nanocarriers.

[159] Rodrigues P.R., Vieira R.P. (2019). Advances in atom-transfer radical polymerization for drug delivery applications. Eur. Polym. J..

[160] Hasannia M., Aliabadi A., Abnous K., Taghdisi S.M., Ramezani M., Alibolandi M. (2022). Synthesis of block copolymers used in polymersome fabrication: Application in drug delivery. J. Control. Release.

[161] Mohammadi M., Ramezani M., Abnous K., Alibolandi M. (2017). Biocompatible polymersomes-based cancer theranostics: Towards multifunctional nanomedicine. Int. J. Pharm..

[162] Kim M.S., Lee D.S. (2010). Biodegradable and pH-sensitive polymersome with tuning permeable membrane for drug delivery carrier. Chem. Comm..

[163] Villani S., Adami R., Reverchon E., Ferretti A.M., Ponti A., Lepretti M., Caputo I., Izzo L. (2017). pH-sensitive polymersomes: Controlling swelling via copolymer structure and chemical composition. J. Drug Target..

[164] Wei P., Sun M., Yang B., Xiao J., Du J. (2020). Ultrasound-responsive polymersomes capable of endosomal escape for efficient cancer therapy. J. Control. Release.

[165] Miele Y., Mingotaud A.-F., Caruso E., Malacarne M.C., Izzo L., Lonetti B., Rossi F. (2021). Hybrid giant lipid vesicles incorporating a PMMA-based copolymer. Biochim. Biophys. Acta BBA Gen. Subj..

[166] Dadhwal S., Lee A., Goswami S.K., Hook S., Gamble A.B. (2021). Synthesis and formulation of self-immolative PEG-aryl azide block copolymers and click-to-release reactivity with trans-cyclooctene. J. Polym. Sci..

[167] Vasile C., Vasile C. (2019). Polymeric nanomaterials: Recent developments, properties and medical applications. Polymeric Nanomaterials in Nanotherapeutics.

[168] Pergushov D.V., Müller A.H., Schacher F.H. (2012). Micellar interpolyelectrolyte complexes. Chem. Soc. Rev..

[169] Ita K. (2020). Polyplexes for gene and nucleic acid delivery: Progress and bottlenecks. Eur. J. Pharm. Sci..

[170] Gombotz W.R., Pettit D.K. (1995). Biodegradable polymers for protein and peptide drug delivery. Bioconjugate Chem..

[171] Bus T., Traeger A., Schubert U.S. (2018). The great escape: How cationic polyplexes overcome the endosomal barrier. J. Mater. Chem. B.

[172] Machtakova M., Thérien-Aubin H., Landfester K. (2022). Polymer nano-systems for the encapsulation and delivery of active biomacromolecular therapeutic agents. Chem. Soc. Rev..

[173] Uchida S., Kataoka K. (2019). Design concepts of polyplex micelles for in vivo therapeutic delivery of plasmid DNA and messenger RNA. J. Biomed. Mater. Res. Part A.

[174] De Ávila Gonçalves S., Vieira R.P. (2020). Current status of ATRP-based materials for gene therapy. React. Funct. Polym..

[175] Xu F., Yang W. (2011). Polymer vectors via controlled/living radical polymerization for gene delivery. Prog. Polym. Sci..

[176] Fliervoet L.A., van Nostrum C.F., Hennink W.E., Vermonden T. (2019). Balancing hydrophobic and electrostatic interactions in thermosensitive polyplexes for nucleic acid delivery. Multifunct. Mater..

[177] Fliervoet L.A., Zhang H., van Groesen E., Fortuin K., Duin N.J., Remaut K., Schiffelers R.M., Hennink W.E., Vermonden T. (2020). Local release of siRNA using polyplex-loaded thermosensitive hydrogels. Nanoscale.

[178] Zhang Y., He J., Cao D., Zhang M., Ni P. (2014). Galactosylated reduction and pH dual-responsive triblock terpolymer Gal-PEEP-a-PCL-ss-PDMAEMA: A multifunctional carrier for the targeted and simultaneous delivery of doxorubicin and DNA. Polym. Chem..

[179] Wang X., Liow S.S., Wu Q., Li C., Owh C., Li Z., Loh X.J., Wu Y.L. (2017). Codelivery for Paclitaxel and Bcl-2 Conversion Gene by PHB-PDMAEMA Amphiphilic Cationic Copolymer for Effective Drug Resistant Cancer Therapy. Macromol. Biosci..

[180] Sun R., Wang Y., Gou P., Zuo M., Li X., Zhu W., Shen Z. (2018). Amphiphilic seven-arm star triblock copolymers with diverse morphologies in aqueous solution induced by crystallization and pH. Chem. Res. Chin. Univ..

[181] Huang B., Chen M., Zhou S., Wu L. (2015). Synthesis and properties of clickable A(B-b-C)20 miktoarm star-shaped block copolymers with a terminal alkyne group. Polym. Chem..

[182] Zhang Y., Bradley M., Geng J. (2019). Photo-controlled one-pot strategy for the synthesis of asymmetric three-arm star polymers. Polym. Chem..

[183] Chmielarz P. (2017). Synthesis of inositol-based star polymers through low ppm ATRP methods. Polym. Adv. Technol..

[184] Zhou P., Liu Y.-Y., Niu L.-Y., Zhu J. (2015). Self-assemblies of the six-armed star triblock ABC copolymer: pH-tunable morphologies and drug release. Polym. Chem..

[185] Huang L.-m., Li L.-d., Shang L., Zhou Q.-h., Lin J. (2016). Preparation of pH-sensitive micelles from miktoarm star block copolymers by ATRP and their application as drug nanocarriers. React. Funct. Polym..

[186] Neugebauer D., Odrobińska J., Bielas R., Mielańczyk A. (2016). Design of systems based on 4-armed star-shaped polyacids for indomethacin delivery. New J. Chem..

[187] Liu X., Tian Z., Chen C., Allcock H.R. (2013). UV-cleavable unimolecular micelles: Synthesis and characterization toward photocontrolled drug release carriers. Polym. Chem..

[188] Mendrek B., Fus A., Klarzyńska K., Sieroń A.L., Smet M., Kowalczuk A., Dworak A. (2018). Synthesis, Characterization and Cytotoxicity of Novel Thermoresponsive Star Copolymers of N,N′-Dimethylaminoethyl Methacrylate and Hydroxyl-Bearing Oligo(Ethylene Glycol) Methacrylate. Polymers.

[189] Zheng A., Xue Y., Wei D., Guan Y., Xiao H. (2013). Amphiphilic star block copolymers as gene carrier Part I: Synthesis via ATRP using calix[4]resorcinarene-based initiators and characterization. Mater. Sci. Eng. C.

[190] Mendrek B., Sieroń Ł., Żymełka-Miara I., Binkiewicz P., Libera M., Smet M., Trzebicka B., Sieroń A.L., Kowalczuk A., Dworak A. (2015). Nonviral Plasmid DNA Carriers Based on N,N′-Dimethylaminoethyl Methacrylate and Di(ethylene glycol) Methyl Ether Methacrylate Star Copolymers. Biomacromolecules.

[191] Cho H.Y., Averick S.E., Paredes E., Wegner K., Averick A., Jurga S., Das S.R., Matyjaszewski K. (2013). Star Polymers with a Cationic Core Prepared by ATRP for Cellular Nucleic Acids Delivery. Biomacromolecules.

[192] Kamaly N., Yameen B., Wu J., Farokhzad O.C. (2016). Degradable Controlled-Release Polymers and Polymeric Nanoparticles: Mechanisms of Controlling Drug Release. Chem. Rev..

[193] Yilmaz G. (2019). One-Pot Synthesis of Star Copolymers by the Combination of Metal-Free ATRP and ROP Processes. Polymers.

[194] Mielańczyk A., Kupczak M., Klymenko O., Mielańczyk Ł., Arabasz S., Madej K., Neugebauer D. (2022). The structure–self-assembly relationship in PDMAEMA/polyester miktoarm stars. Polym. Chem..

[195] Xu F., Zheng S.-Z., Luo Y.-L. (2013). Thermosensitive t-PLA-b-PNIPAAm tri-armed star block copolymer nanoscale micelles for camptothecin drug release. J. Polym. Sci. Part A Polym. Chem..

[196] Li C., Wang B., Liu Y., Cao J., Feng T., Chen Y., Luo X. (2013). Synthesis and evaluation of star-shaped poly(ϵ-caprolactone)-poly(2-hydroxyethyl methacrylate) as potential anticancer drug delivery carriers. J. Biomater. Sci. Polym. Ed..

[197] Xiong D., Yao N., Gu H., Wang J., Zhang L. (2017). Stimuli-responsive shell cross-linked micelles from amphiphilic four-arm star copolymers as potential nanocarriers for “pH/redox-triggered” anticancer drug release. Polymer.

[198] Yuan H., Chi H., Yuan W. (2016). A star-shaped amphiphilic block copolymer with dual responses: Synthesis, crystallization, self-assembly, redox and LCST–UCST thermoresponsive transition. Polym. Chem..

[199] Mielańczyk A., Kupczak M., Burek M., Mielańczyk Ł., Klymenko O., Wandzik I., Neugebauer D. (2018). Functional (mikto)stars and star-comb copolymers from d-gluconolactone derivative: An efficient route for tuning the architecture and responsiveness to stimuli. Polymer.

[200] Huang X., Xiao Y., Lang M. (2011). Synthesis and self-assembly behavior of six-armed block copolymers with pH- and thermo-responsive properties. Macromol. Res..

[201] Teng X., Zhang P., Liu T., Xin J., Zhang J. (2020). Biobased miktoarm star copolymer from soybean oil, isosorbide, and caprolactone. J. Appl. Polym. Sci..

[202] Milner S.T. (1991). Polymer brushes. Science.

[203] Bousquet A., Boyer C., Davis T.P., Stenzel M.H. (2010). Electrostatic assembly of functional polymer combs onto gold nanoparticle surfaces: Combining RAFT, click and LbL to generate new hybrid nanomaterials. Polym. Chem..

[204] Peng S., Bhushan B. (2012). Smart polymer brushes and their emerging applications. RSC Adv..

[205] Sun W., Liu W., Wu Z., Chen H. (2020). Chemical surface modification of polymeric biomaterials for biomedical applications. Macromol. Rapid Commun..

[206] Celentano W., Ordanini S., Bruni R., Marocco L., Medaglia P., Rossi A., Buzzaccaro S., Cellesi F. (2021). Complex poly(ε-caprolactone)/poly(ethylene glycol) copolymer architectures and their effects on nanoparticle self-assembly and drug nanoencapsulation. Eur. Polym. J..

[207] Tu X.Y., Meng C., Zhang X.L., Jin M.G., Zhang X.S., Zhao X.Z., Wang Y.F., Ma L.W., Wang B.Y., Liu M.Z. (2018). Fabrication of Reduction-Sensitive Amphiphilic Cyclic Brush Copolymer for Controlled Drug Release. Macromol. Biosci..

[208] Cheng R., Meng F., Deng C., Klok H.-A., Zhong Z. (2013). Dual and multi-stimuli responsive polymeric nanoparticles for programmed site-specific drug delivery. Biomaterials.

[209] Kumar A., Lale S.V., Mahajan S., Choudhary V., Koul V. (2015). ROP and ATRP fabricated dual targeted redox sensitive polymersomes based on pPEGMA-PCL-ss-PCL-pPEGMA triblock copolymers for breast cancer therapeutics. ACS Appl. Mater. Interfaces.

[210] Nehate C., Moothedathu Raynold A.A., Haridas V., Koul V. (2018). Comparative assessment of active targeted redox sensitive polymersomes based on pPEGMA-SS-PLA diblock copolymer with marketed nanoformulation. Biomacromolecules.

[211] Zhao P., Deng M., Yang Y., Zhang J., Zhang Y. (2021). Synthesis and Self-Assembly of Thermoresponsive Biohybrid Graft Copolymers Based on a Combination of Passerini Multicomponent Reaction and Molecular Recognition. Macromol. Rapid Commun..

[212] Rodrigues P.R., Wang X., Li Z., Lyu J., Wang W., Vieira R.P. (2023). A new nano hyperbranched β-pinene polymer: Controlled synthesis and nonviral gene delivery. Colloids Surf. B Biointerfaces.

[213] Newland B., Tai H., Zheng Y., Velasco D., Di Luca A., Howdle S.M., Alexander C., Wang W., Pandit A. (2010). A highly effective gene delivery vector–hyperbranched poly(2-(dimethylamino)ethyl methacrylate) from in situ deactivation enhanced ATRP. Chem. Comm..

[214] Flejszar M., Chmielarz P., Smenda J., Wolski K. (2021). Following principles of green chemistry: Low ppm photo-ATRP of DMAEMA in water/ethanol mixture. Polymer.

[215] Bouzenna H., Hfaiedh N., Giroux-Metges M.-A., Elfeki A., Talarmin H. (2017). Potential protective effects of alpha-pinene against cytotoxicity caused by aspirin in the IEC-6 cells. Biomed. Pharmacother..

[216] Cohen-Karni D., Kovaliov M., Li S., Jaffee S., Tomycz N.D., Averick S. (2017). Fentanyl initiated polymers prepared by atrp for targeted delivery. Bioconjugate Chem..

[217] Lutz J.F. (2008). Polymerization of oligo (ethylene glycol)(meth) acrylates: Toward new generations of smart biocompatible materials. J. Polym. Sci. Part A Polym. Chem..

[218] Li S., Cohen-Karni D., Kallick E., Edington H., Averick S. (2016). Post-polymerization functionalization of epoxide-containing copolymers in trifluoroethanol for synthesis of polymer-drug conjugates. Polymer.

[219] Zhang Y., Jiang Q., Wojnilowicz M., Pan S., Ju Y., Zhang W., Liu J., Zhuo R., Jiang X. (2018). Acid-sensitive poly (β-cyclodextrin)-based multifunctional supramolecular gene vector. Polym. Chem..

[220] Nehate C., Moothedathu Raynold A.A., Koul V. (2017). ATRP fabricated and short chain polyethylenimine grafted redox sensitive polymeric nanoparticles for codelivery of anticancer drug and siRNA in cancer therapy. ACS Appl. Mater. Interfaces.

[221] Ensafi A.A., Khoddami E., Nabiyan A., Rezaei B. (2017). Study the role of poly (diethyl aminoethyl methacrylate) as a modified and grafted shell for TiO2 and ZnO nanoparticles, application in flutamide delivery. React. Funct. Polym..

[222] Alotaibi K.M., Almethen A.A., Beagan A.M., Alfhaid L.H., Ahamed M., El-Toni A.M., Alswieleh A.M. (2021). Poly (oligo (ethylene glycol) methyl ether methacrylate) Capped pH-Responsive Poly (2-(diethylamino) ethyl methacrylate) Brushes Grafted on Mesoporous Silica Nanoparticles as Nanocarrier. Polymers.

[223] Alswieleh A.M., Beagan A.M., Alsheheri B.M., Alotaibi K.M., Alharthi M.D., Almeataq M.S. (2020). Hybrid mesoporous silica nanoparticles grafted with 2-(tert-butylamino) ethyl methacrylate-b-poly (ethylene glycol) methyl ether methacrylate diblock brushes as drug nanocarrier. Molecules.

[224] Alswieleh A.M., Alshahrani M.M., Alzahrani K.E., Alghamdi H.S., Niazy A.A., Alsilme A.S., Beagan A.M., Alsheheri B.M., Alghamdi A.A., Almeataq M.S. (2019). Surface modification of pH-responsive poly (2-(tert-butylamino) ethyl methacrylate) brushes grafted on mesoporous silica nanoparticles. Des. Monomers Polym..

[225] Chen T., Wu W., Xiao H., Chen Y., Chen M., Li J. (2016). Intelligent drug delivery system based on mesoporous silica nanoparticles coated with an ultra-pH-sensitive gatekeeper and poly (ethylene glycol). ACS Macro Lett..

[226] Peng S., Yuan X., Lin W., Cai C., Zhang L. (2019). pH-responsive controlled release of mesoporous silica nanoparticles capped with Schiff base copolymer gatekeepers: Experiment and molecular dynamics simulation. Colloids Surf. B Biointerfaces.

[227] Huang L., Liu M., Mao L., Xu D., Wan Q., Zeng G., Shi Y., Wen Y., Zhang X., Wei Y. (2017). Preparation and controlled drug delivery applications of mesoporous silica polymer nanocomposites through the visible light induced surface-initiated ATRP. Appl. Surf. Sci..

[228] Beagan A.M., Alghamdi A.A., Lahmadi S.S., Halwani M.A., Almeataq M.S., Alhazaa A.N., Alotaibi K.M., Alswieleh A.M. (2020). Folic acid-terminated poly (2-diethyl amino ethyl methacrylate) brush-gated magnetic mesoporous nanoparticles as a smart drug delivery system. Polymers.

[229] He X., Wu X., Cai X., Lin S., Xie M., Zhu X., Yan D. (2012). Functionalization of magnetic nanoparticles with dendritic–linear–brush-like triblock copolymers and their drug release properties. Langmuir.

[230] Ellis E., Zhang K., Lin Q., Ye E., Poma A., Battaglia G., Loh X.J., Lee T.-C. (2017). Biocompatible pH-responsive nanoparticles with a core-anchored multilayer shell of triblock copolymers for enhanced cancer therapy. J. Mater. Chem. B.

[231] Xiong D., Zhang X., Peng S., Gu H., Zhang L. (2018). Smart pH-sensitive micelles based on redox degradable polymers as DOX/GNPs carriers for controlled drug release and CT imaging. Colloids Surf. B: Biointerfaces.

[232] Yao N., Lin W., Zhang X., Gu H., Zhang L. (2016). Amphiphilic β-cyclodextrin-based star-like block copolymer unimolecular micelles for facile in situ preparation of gold nanoparticles. J. Polym. Sci. Part A Polym. Chem..

[233] Li C., Wang J., Wang Y., Gao H., Wei G., Huang Y., Yu H., Gan Y., Wang Y., Mei L. (2019). Recent progress in drug delivery. Acta Pharm. Sin. B.

[234] Paolino D., Sinha P., Fresta M., Ferrari M. (2006). Drug Delivery Systems. Encyclopedia of Medical Devices and Instrumentation.

[235] Huang Y., Cole S.P.C., Cai T., Cai Y. (2016). Applications of nanoparticle drug delivery systems for the reversal of multidrug resistance in cancer (Review). Oncol. Lett..

[236] Leppert W., Malec–Milewska M., Zajaczkowska R., Wordliczek J. (2018). Transdermal and Topical Drug Administration in the Treatment of Pain. Molecules.

[237] Khandare J., Minko T. (2006). Polymer–drug conjugates: Progress in polymeric prodrugs. Prog. Polym. Sci..

[238] Neeraj Agrawal R., Alok Mukerji A.J. (2013). Polymeric Prodrugs: Recent Achievements and General Strategies. J. Antivir. Antiretrovir..

[239] Ekladious I., Colson Y.L., Grinstaff M.W. (2019). Polymer–drug conjugate therapeutics: Advances, insights and prospects. Nat. Rev. Drug Discov..

[240] Ulery B.D., Nair L.S., Laurencin C.T. (2011). Biomedical applications of biodegradable polymers. J. Polym. Sci. Part B Polym. Phys..

[241] Dong R., Zhou Y., Huang X., Zhu X., Lu Y., Shen J. (2015). Functional Supramolecular Polymers for Biomedical Applications. Adv. Mater..

[242] Singhvi M.S., Zinjarde S.S., Gokhale D.V. (2019). Polylactic acid: Synthesis and biomedical applications. J. Appl. Microbiol..

[243] Ringsdorf H. (1975). Structure and properties of pharmacologically active polymers. J. Polym. Sci. Polym. Symp..

[244] Liu S., Maheshwari R., Kiick K.L. (2009). Polymer-Based Therapeutics. Macromolecules.

[245] Parveen S., Arjmand F., Tabassum S. (2019). Clinical developments of antitumor polymer therapeutics. RSC Adv..

[246] Ribelli T.G., Lorandi F., Fantin M., Matyjaszewski K. (2019). Atom Transfer Radical Polymerization: Billion Times More Active Catalysts and New Initiation Systems. Macromol. Rapid Commun..

[247] Briand V.A., Kumar C.V., Kasi R.M. (2011). Protein-Polymer Conjugates. Encyclopedia of Polymer Science and Technology.

[248] Zhao W., Liu F., Chen Y., Bai J., Gao W. (2015). Synthesis of well-defined protein–polymer conjugates for biomedicine. Polymer.

[249] Wang Y., Wu C. (2018). Site-Specific Conjugation of Polymers to Proteins. Biomacromolecules.

[250] Kovaliov M., Cohen-Karni D., Burridge K.A., Mambelli D., Sloane S., Daman N., Xu C., Guth J., Kenneth Wickiser J., Tomycz N. (2018). Grafting strategies for the synthesis of active DNase I polymer biohybrids. Eur. Polym. J..

[251] Silva A.R.P., Guimarães M.S., Rabelo J., Belén L.H., Perecin C.J., Farías J.G., Santos J.H.P.M., Rangel-Yagui C.O. (2022). Recent advances in the design of antimicrobial peptide conjugates. J. Mater. Chem. B.

[252] Pelegri-O’Day E.M., Maynard H.D. (2016). Controlled Radical Polymerization as an Enabling Approach for the Next Generation of Protein–Polymer Conjugates. Acc. Chem. Res..

[253] Kaupbayeva B., Boye S., Munasinghe A., Murata H., Matyjaszewski K., Lederer A., Colina C.M., Russell A.J. (2021). Molecular Dynamics-Guided Design of a Functional Protein–ATRP Conjugate That Eliminates Protein–Protein Interactions. Bioconjug. Chem..

[254] Bontempo D., Heredia K.L., Fish B.A., Maynard H.D. (2004). Cysteine-Reactive Polymers Synthesized by Atom Transfer Radical Polymerization for Conjugation to Proteins. J. Am. Chem. Soc..

[255] Patel A., Graeff-Armas L., Ross M., Goldner W., Niederhuber J.E., Armitage J.O., Kastan M.B., Doroshow J.H., Tepper J.E. (2020). 35—Hypercalcemia. Abeloff’s Clinical Oncology.

[256] Sayers C.T., Mantovani G., Ryan S.M., Randev R.K., Keiper O., Leszczyszyn O.I., Blindauer C., Brayden D.J., Haddleton D.M. (2009). Site-specific N-terminus conjugation of poly(mPEG1100) methacrylates to salmon calcitonin: Synthesis and preliminary biological evaluation. Soft Matter.

[257] Baker S.L., Kaupbayeva B., Lathwal S., Das S.R., Russell A.J., Matyjaszewski K. (2019). Atom Transfer Radical Polymerization for Biorelated Hybrid Materials. Biomacromolecules.

[258] Yoshihara E., Sasaki M., Nabil A., Iijima M., Ebara M. (2022). Temperature Responsive Polymer Conjugate Prepared by “Grafting from” Proteins toward the Adsorption and Removal of Uremic Toxin. Molecules.

[259] Cummings C.S., Fein K., Murata H., Ball R.L., Russell A.J., Whitehead K.A. (2017). ATRP-grown protein-polymer conjugates containing phenylpiperazine selectively enhance transepithelial protein transport. J. Control. Release.

[260] Trzebicka B., Robak B., Trzcinska R., Szweda D., Suder P., Silberring J., Dworak A. (2013). Thermosensitive PNIPAM-peptide conjugate—Synthesis and aggregation. Eur. Polym. J..

[261] Cohen-Karni D., Kovaliov M., Ramelot T., Konkolewicz D., Graner S., Averick S. (2017). Grafting challenging monomers from proteins using aqueous ICAR ATRP under bio-relevant conditions. Polym. Chem..

[262] Konkolewicz D., Magenau A.J.D., Averick S.E., Simakova A., He H., Matyjaszewski K. (2012). ICAR ATRP with ppm Cu Catalyst in Water. Macromolecules.

[263] Moncalvo F., Lacroce E., Franzoni G., Altomare A., Fasoli E., Aldini G., Sacchetti A., Cellesi F. (2022). Selective Protein Conjugation of Poly(glycerol monomethacrylate) and Poly(polyethylene glycol methacrylate) with Tunable Topology via Reductive Amination with Multifunctional ATRP Initiators for Activity Preservation. Macromolecules.

[264] Kaupbayeva B., Murata H., Rule G.S., Matyjaszewski K., Russell A.J. (2022). Rational Control of Protein–Protein Interactions with Protein-ATRP-Generated Protease-Sensitive Polymer Cages. Biomacromolecules.

[265] Baker S.L., Munasinghe A., Murata H., Lin P., Matyjaszewski K., Colina C.M., Russell A.J. (2018). Intramolecular Interactions of Conjugated Polymers Mimic Molecular Chaperones to Stabilize Protein–Polymer Conjugates. Biomacromolecules.

[266] Li P., Sun M., Xu Z., Liu X., Zhao W., Gao W. (2018). Site-Selective in Situ Growth-Induced Self-Assembly of Protein–Polymer Conjugates into pH-Responsive Micelles for Tumor Microenvironment Triggered Fluorescence Imaging. Biomacromolecules.

[267] Liu X., Sun M., Sun J., Hu J., Wang Z., Guo J., Gao W. (2018). Polymerization Induced Self-Assembly of a Site-Specific Interferon α-Block Copolymer Conjugate into Micelles with Remarkably Enhanced Pharmacology. J. Am. Chem. Soc..

[268] Baker S.L., Munasinghe A., Kaupbayeva B., Rebecca Kang N., Certiat M., Murata H., Matyjaszewski K., Lin P., Colina C.M., Russell A.J. (2019). Transforming protein-polymer conjugate purification by tuning protein solubility. Nat. Commun..

[269] Zhu B., Lu D., Ge J., Liu Z. (2011). Uniform polymer–protein conjugate by aqueous AGET ATRP using protein as a macroinitiator. Acta Biomater..

[270] Averick S.E., Bazewicz C.G., Woodman B.F., Simakova A., Mehl R.A., Matyjaszewski K. (2013). Protein–polymer hybrids: Conducting ARGET ATRP from a genetically encoded cleavable ATRP initiator. Eur. Polym. J..

[271] Kovaliov M., Cheng C., Cheng B., Averick S. (2018). Grafting-from lipase: Utilization of a common amino acid residue as a new grafting site. Polym. Chem..

[272] Fu L., Wang Z., Lathwal S., Enciso A.E., Simakova A., Das S.R., Russell A.J., Matyjaszewski K. (2018). Synthesis of Polymer Bioconjugates via Photoinduced Atom Transfer Radical Polymerization under Blue Light Irradiation. ACS Macro Lett..

[273] Olszewski M., Jeong J., Szczepaniak G., Li S., Enciso A., Murata H., Averick S., Kapil K., Das S.R., Matyjaszewski K. (2022). Sulfoxide-Containing Polyacrylamides Prepared by PICAR ATRP for Biohybrid Materials. ACS Macro Lett..

[274] Charan H., Kinzel J., Glebe U., Anand D., Garakani T.M., Zhu L., Bocola M., Schwaneberg U., Böker A. (2016). Grafting PNIPAAm from β-barrel shaped transmembrane nanopores. Biomaterials.

[275] Elvira C., Gallardo A., Roman J., Cifuentes A. (2005). Covalent Polymer-Drug Conjugates. Molecules.

[276] Stanley T.H. (2014). The Fentanyl Story. J. Pain.

[277] Stanley T.H., Eger Ii E.I., Saidman L.J., Westhorpe R.N. (2014). The History of Opioid Use in Anesthetic Delivery. The Wondrous Story of Anesthesia.

[278] Li Y., Liu R., Yang J., Ma G., Zhang Z., Zhang X. (2014). Dual sensitive and temporally controlled camptothecin prodrug liposomes codelivery of siRNA for high efficiency tumor therapy. Biomaterials.

[279] Mei C., Lei L., Tan L.-M., Xu X.-J., He B.-M., Luo C., Yin J.-Y., Li X., Zhang W., Zhou H.-H. (2020). The role of single strand break repair pathways in cellular responses to camptothecin induced DNA damage. Biomed. Pharmacother..

[280] Fan X., Lin X., Ruan Q., Wang J., Yang Y., Sheng M., Zhou W., Kai G., Hao X. (2022). Research progress on the biosynthesis and metabolic engineering of the anti-cancer drug camptothecin in Camptotheca acuminate. Ind. Crops Prod..

[281] Plichta A., Kowalczyk S., Kamiński K., Wasyłeczko M., Więckowski S., Olędzka E., Nałęcz-Jawecki G., Zgadzaj A., Sobczak M. (2017). ATRP of Methacrylic Derivative of Camptothecin Initiated with PLA toward Three-Arm Star Block Copolymer Conjugates with Favorable Drug Release. Macromolecules.

[282] Gao Y.-E., Bai S., Shi X., Hou M., Ma X., Zhang T., Xiao B., Xue P., Kang Y., Xu Z. (2018). Irinotecan delivery by unimolecular micelles composed of reduction-responsive star-like polymeric prodrug with high drug loading for enhanced cancer therapy. Colloids Surf. B: Biointerfaces.

[283] Chen W., Shah L.A., Yuan L., Siddiq M., Hu J., Yang D. (2014). Polymer–paclitaxel conjugates based on disulfide linkers for controlled drug release. RSC Adv..

[284] Dong Y., Du P., Pei M., Liu P. (2019). Design, postpolymerization conjugation and self-assembly of a di-block copolymer-based prodrug for tumor intracellular acid-triggered DOX release. J. Mater. Chem. B.

[285] Odrobińska J., Neugebauer D. (2019). Retinol derivative as bioinitiator in the synthesis of hydroxyl-functionalized polymethacrylates for micellar delivery systems. Express Polym. Lett..

[286] Zaborniak I., Chmielarz P., Matyjaszewski K. (2020). Synthesis of Riboflavin-Based Macromolecules through Low ppm ATRP in Aqueous Media. Macromol. Chem. Phys..

[287] Plichta A., Kowalczyk S., Olędzka E., Sobczak M., Strawski M. (2018). Effect of structural factors on release profiles of camptothecin from block copolymer conjugates with high load of drug. Int. J. Pharm..

[288] Qiu L., Liu Q., Hong C.-Y., Pan C.-Y. (2015). Unimolecular micelles of camptothecin-bonded hyperbranched star copolymers via β-thiopropionate linkage: Synthesis and drug delivery. J. Mater. Chem. B.

[289] Bai S., Jia D., Ma X., Liang M., Xue P., Kang Y., Xu Z. (2021). Cylindrical polymer brushes-anisotropic unimolecular micelle drug delivery system for enhancing the effectiveness of chemotherapy. Bioact. Mater..

[290] Bai S., Gao Y.-E., Ma X., Shi X., Hou M., Xue P., Kang Y., Xu Z. (2018). Reduction stimuli-responsive unimolecular polymeric prodrug based on amphiphilic dextran-framework for antitumor drug delivery. Carbohydr. Polym..

[291] Bai S., Hou M., Shi X., Chen J., Ma X., Gao Y.-E., Wang Y., Xue P., Kang Y., Xu Z. (2018). Reduction-active polymeric prodrug micelles based on α-cyclodextrin polyrotaxanes for triggered drug release and enhanced cancer therapy. Carbohydr. Polym..

[292] Chen X., Parelkar S.S., Henchey E., Schneider S., Emrick T. (2012). PolyMPC–Doxorubicin Prodrugs. Bioconjugate Chem..

[293] Lale S.V., Ravindran Girija A., Aravind A., Kumar D.S., Koul V. (2014). AS1411 Aptamer and Folic Acid Functionalized pH-Responsive ATRP Fabricated pPEGMA–PCL–pPEGMA Polymeric Nanoparticles for Targeted Drug Delivery in Cancer Therapy. Biomacromolecules.

[294] Wang H., Xu F., Li D., Liu X., Jin Q., Ji J. (2013). Bioinspired phospholipid polymer prodrug as a pH-responsive drug delivery system for cancer therapy. Polym. Chem..

[295] Pelras T., Duong H.T.T., Kim B.J., Hawkett B.S., Müllner M. (2017). A ‘grafting from’ approach to polymer nanorods for pH-triggered intracellular drug delivery. Polymer.

[296] Rickert E.L., Trebley J.P., Peterson A.C., Morrell M.M., Weatherman R.V. (2007). Synthesis and Characterization of Bioactive Tamoxifen-Conjugated Polymers. Biomacromolecules.

[297] Jin Q., Mitschang F., Agarwal S. (2011). Biocompatible Drug Delivery System for Photo-Triggered Controlled Release of 5-Fluorouracil. Biomacromolecules.

[298] Giacomelli C., Schmidt V., Borsali R. (2007). Nanocontainers Formed by Self-Assembly of Poly(ethylene oxide)-b-poly(glycerol monomethacrylate)−Drug Conjugates. Macromolecules.

[299] Zaborniak I., Chmielarz P., Wolski K. (2020). Riboflavin-induced metal-free ATRP of (meth) acrylates. Eur. Polym. J..

[300] Fairbanks B.D., Gunatillake P.A., Meagher L. (2015). Biomedical applications of polymers derived by reversible addition–fragmentation chain-transfer (RAFT). Adv. Drug Deliv. Rev..

